# Sulf1 regulates inflammation via modulation of TGF-β dependent epicardial activation

**DOI:** 10.1038/s44319-026-00910-5

**Published:** 2026-08-27

**Authors:** Gursimran Kaur Bajwa, Gülsüm Kayman Kürekçi, Rubén Marín-Juez

**Affiliations:** 1 Centre de Recherche Azrieli, Centre Hospitalier Universitaire Sainte-Justine, Montréal, QC Canada; 2 Department of Pathology and Cell Biology, Faculty of Medicine, Université de Montréal, Montréal, QC Canada

**Keywords:** Cardiovascular System, Signal Transduction, Stem Cells & Regenerative Medicine

## Abstract

Unlike adult humans, zebrafish can regenerate their hearts after injury. After cardiac injury, the epicardium is rapidly activated to contribute both signaling factors and progenitor cells that are essential to establish a pro-regenerative niche. Concurrent with epicardial activation, cardiac injury triggers a robust inflammatory cascade. While the epicardial response is crucial for cardiac regeneration, the mechanisms by which it modulates the immune landscape remain largely unknown. Sulfatases have emerged as important modulators of inflammation through regulating cytokine and growth factor signaling. Here we find that the zebrafish epicardium upregulates the endo-sulfatase Sulf1 after injury. Using newly generated gain- and loss-of-function tools, we show that Sulf1 regulates epicardial activation and regeneration via TGF-β signaling. Abrogation of *sulf1* results in cardiomyocyte regeneration defects and increased fibrosis. Moreover, disrupted TGF-β signaling in *sulf1* mutants stimulates inflammation and macrophage recruitment. scRNA-seq analyses reveal that epicardial Sulf1 regulates the recruitment of pro-inflammatory macrophage subsets. Epicardial overexpression of *sulf1* reverses TGF-β signaling and inflammation phenotypes. These data show that Sulf1 modulates TGF-β-dependent epicardial activation to regulate cardiac inflammation during heart regeneration.

## Synopsis

Sulf1 is a critical modulator of cardiac regeneration through its dual role in controlling TGF-β-dependent epicardial activation and shaping the inflammatory response. Our data establish the Sulf1-TGF-β axis as a driver of both epicardial and immune responses after injury.

- The epicardium upregulates *sulf1* in response to cardiac injury and this induction is required for TGF-β mediated epicardial activation.
- Sulf1 regulates epicardial TGF-β signaling to facilitate cardiomyocyte regeneration and limit late-stage fibrosis.
- Sulf1 mediated TGF-β signaling modulates the macrophage response after cardiac injury.
- Epicardial Sulf1 regulates the epicardial-immune crosstalk during regeneration.

## Abstract (web summary)

Sulf1 is a critical modulator of cardiac regeneration through its dual role in controlling TGF-β-dependent epicardial activation and shaping the inflammatory response. Our data establish the Sulf1-TGF-β axis as a driver of both epicardial and immune responses after injury.

## Graphical abstract

**Figure:**
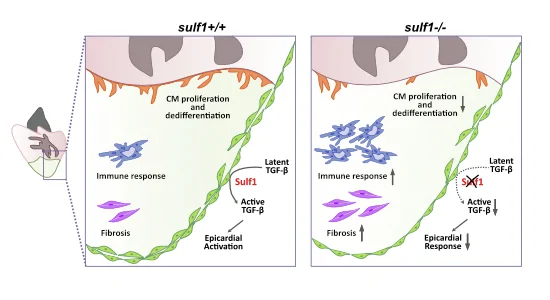

## Introduction

Myocardial infarction (MI) remains one of the leading causes of death worldwide, posing a global health challenge (Salari et al, 2023). Following MI, the adult mammalian heart is unable to replace the damaged cardiac muscle. Cardiac injury results in irreversible loss of cardiomyocytes (CMs), which are replaced by a fibrotic scar, leading to pathological cardiac remodeling and organ failure (Verjans et al, 2020; Vujic et al, 2020; Zhang et al, 2021).

Unlike adult mammals, the adult zebrafish heart possesses a remarkable regenerative capacity (González-Rosa et al, 2011; Major and Poss, 2007; Poss et al, 2002; Ross Stewart et al, 2022). Following cardiac injury in zebrafish, cellular debris is cleared from the affected region, and fibrotic tissue develops, mirroring the mammalian response. In zebrafish, however, this fibrotic tissue subsequently resolves and is replaced by regenerated myocardium (González-Rosa et al, 2011; Major and Poss, 2007; Poss et al, 2002; Ross Stewart et al, 2022). Efficient cardiac regeneration in zebrafish relies on the dynamic interplay of multiple cardiac cell populations through processes including inflammation (Bevan et al, 2020; Iribarne, 2021; Peterson et al, 2024; Wei et al, 2023), revascularization (Marín-Juez et al, 2016, 2019), CM renewal (Beisaw and Wu, 2024; Brezitski et al, 2021; Tsedeke et al, 2021), endocardial (Kikuchi et al, 2011) and epicardial activation (Kikuchi et al, 2011; Lepilina et al, 2006), and tissue remodeling (Garcia-Puig et al, 2019).

The epicardium plays a central role during both cardiac repair and regeneration (Cao and Poss, 2018; Kayman Kürekçi et al, 2026; Kikuchi et al, 2011; Lepilina et al, 2006; Masters and Riley, 2014). Normally a quiescent mesothelial layer, the epicardium becomes activated after injury to undergo epithelial-to-mesenchymal transition (EMT), giving rise to epicardium-derived cells (EPDCs)(Foglio et al, 2024). EPDCs contribute to various cardiac lineages and secrete paracrine factors that promote cardiac regeneration (Cao and Poss, 2018; González-Rosa et al, 2012; Kikuchi et al, 2011). Activation of the epicardium occurs within the first 24 h after injury (Ross Stewart et al, 2022), coinciding with the initiation of the inflammatory response and supporting the possibility of an immuno-epicardial regulatory crosstalk.

In zebrafish, tight regulation and swift resolution of the early immune response are essential to enable regeneration (Bevan et al, 2020). In contrast, the adult mammalian heart exhibits a prolonged inflammatory response that leads to fibrotic scarring (Aurora et al, 2014; Godwin et al, 2017). Understanding how epicardial signals integrate with the immune response during regeneration may reveal key mechanisms underlying cardiac regeneration.

Heparan sulfate proteoglycans (HSPGs) are essential components of the cell surface and extracellular matrix (ECM) that regulate cell-ECM attachment, cell–cell interactions, inflammation, and tissue remodeling (Hassan et al, 2021; Sarrazin et al, 2011). Their function is largely mediated by heparan sulfate (HS) chains, which bind signaling molecules through specific sulfation patterns (Hassan et al, 2021; Marques et al, 2021; Sarrazin et al, 2011). Sulf1 and Sulf2, extracellular endo-sulfatases, selectively remove 6-*O*-sulfate groups from HS chains (Hassan et al, 2021; Marques et al, 2021), thereby modulating ligand interactions and fine-tuning signaling pathways involved in development, inflammation, and regeneration (Kim et al, 2021; Korf-Klingebiel et al, 2019; Meyers et al, 2013; Okada et al, 2017; preprint: Redpath et al, 2025).

Recent preprint data indicate that epicardial *Sulf1* fine-tunes HSPG-dependent signaling to modulate key processes during heart development (preprint: Redpath et al, 2025). During cardiac regeneration, developmental programs are reactivated to enable tissue replenishment (Beisaw and Wu, 2024; Tsedeke et al, 2021), further supporting the notion that Sulf1 induction could stimulate regenerative processes.

Here, we find that *sulf1* is dynamically expressed in the regenerating epicardium, showing a strong upregulation early after cardiac injury. This early induction temporally parallels epicardial activation and inflammatory responses triggered by the injury, raising the possibility that Sulf1 regulates the coordination of these events. Using newly developed mutants, we show that Sulf1 regulates epicardial activation via TGF-β signaling. Loss of *sulf1* hinders epicardial TGF-β signaling activation upon injury and stimulates persistent fibrosis, impeding cardiac regeneration. Interestingly, we find that the inflammatory response is exacerbated in *sulf1* mutant hearts after injury. Our data indicate that this response is regulated by epicardial TGF-β signaling, revealing a link between epicardial activation and inflammation after injury. Our results identify alterations in injury-responsive macrophage subsets as the underlying cause of the inflammatory phenotype observed in *sulf1* mutants. Using tissue-specific gain-of-function approaches, we show that epicardial *sulf1* overexpression stimulates TGF-β signaling and epicardial activation. Notably, by enhancing *sulf1* expression in EPDCs, we are able to limit inflammation and reduce fibrosis in injured hearts.

Overall, these results highlight Sulf1 as an injury-induced EPDC factor that modulates epicardial TGF-β signaling to coordinate inflammation and support cardiac regeneration.

## Results

### EPDCs upregulate *sulf1* in response to cardiac injury

**Figure 1:**
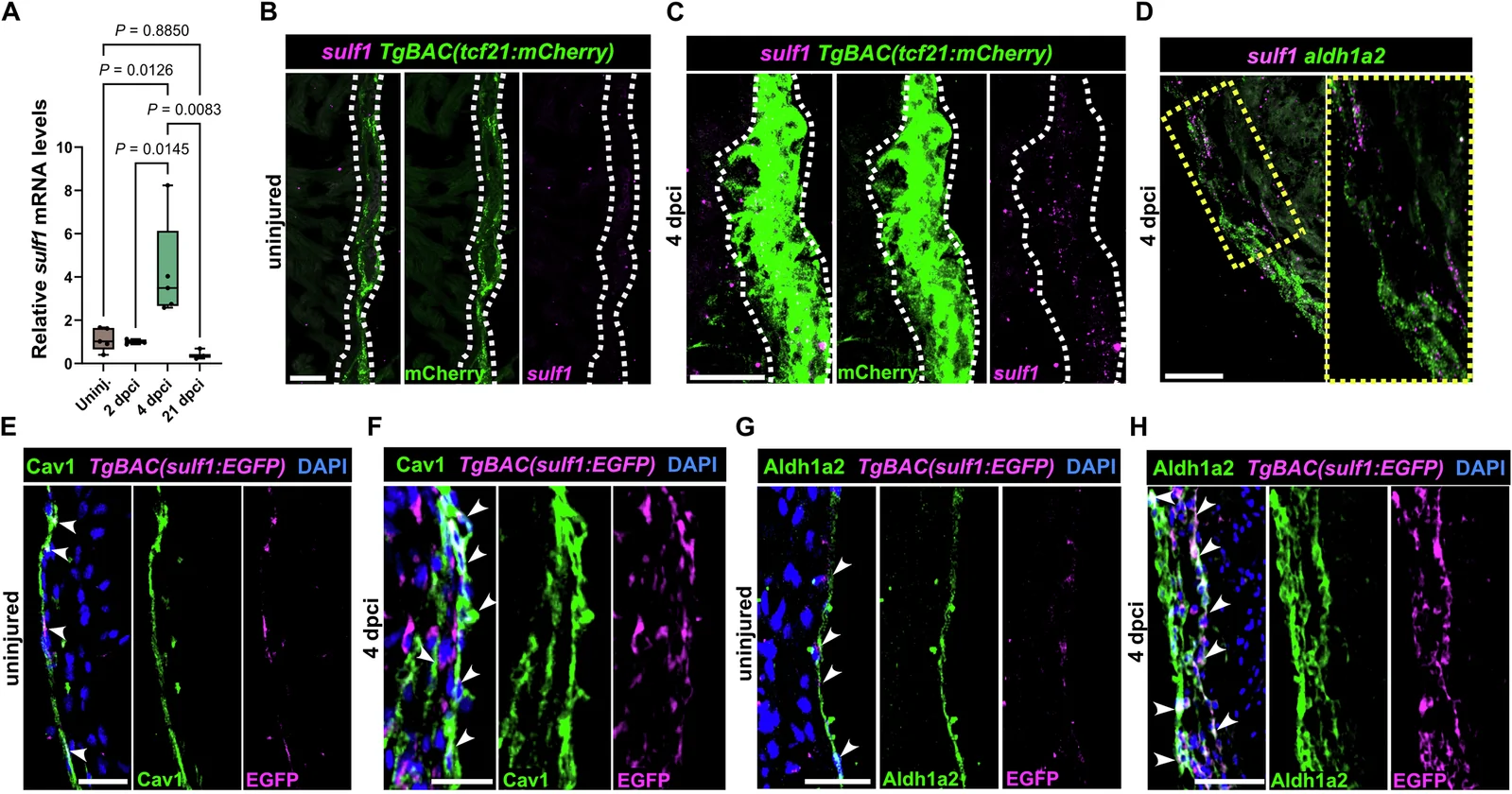
EPDCs upregulate *sulf1* in response to cardiac injury. (**A**) RT-qPCR analysis of *sulf1* mRNA levels in uninjured (*n* = 5) and 2 (*n* = 4), 4 (*n* = 5), and 21 (*n* = 3) dpci ventricles. (**B**) In situ HCR for *sulf1* (magenta) and immunostaining for mCherry (EPDCs, green) on sections of *TgBAC(tcf21:mCherry)* uninjured ventricles. (**C**) In situ HCR for *sulf1* (magenta) and immunostaining for mCherry (EPDCs, green) on sections of *TgBAC(tcf21:mCherry)* ventricles at 4 dpci. (**D**) HCR for *sulf1* (magenta) and *aldh1a2* (green) on wild-type ventricular sections at 4 dpci. The inset shows a high-magnification image of border zone EPDCs expressing *sulf1* and *aldh1a2*. (**E**, **F**) Ventricular sections of wild-type uninjured ventricles (**E**) and at 4 dpci (**F**) stained for *sulf1:*EGFP expression (magenta), Cav1 (EPDCs, green), and DAPI (DNA, blue). White arrowheads point to *sulf1:*EGFP^+^ Cav1^+^ cells. (**G**, **H**) Ventricular sections of wild-type uninjured ventricles (**G**) and at 4 dpci (**H**) stained for *sulf1:*EGFP expression (magenta), Aldh1a2 (EPDCs, green), and DAPI (DNA, blue). White arrowheads point to *sulf1:*EGFP^+^ Aldh1a2^+^ cells. Scale bars: 30 µm. Statistical test: one-way ANOVA followed by Tukey’s post hoc test (**A**). Source data are available online for this figure.

As a first step toward understanding the role of Sulf1 in cardiac regeneration, we analyzed its expression dynamics at different timepoints after ventricular cryoinjury. To this end, we performed real-time quantitative polymerase chain reaction (RT-qPCR) analyses on uninjured, 2, 4, and 21 days post-cryoinjury (dpci) zebrafish ventricles. We found that *sulf1* expression was significantly upregulated at 4 dpci and returned to basal levels by 21 dpci (Fig. 1A). To identify the cell-type(s) expressing *sulf1* during cardiac regeneration, we performed in situ hybridization chain reaction (HCR) for *sulf1* on wild-type ventricular sections. In uninjured ventricles, *sulf1* expression was barely detectable (Fig. 1B). At 4 dpci, we observed that *sulf1* was upregulated in the EPDCs around the injured area, as shown by colocalization with *tcf21*:mCherry^+^ cells (Kikuchi et al, 2011; Xia et al, 2022) (Fig. 1C) and *aldh1a2* (Fig. 1D). *sulf1* expression was persistent in EPDCs at 7 dpci (Appendix Fig. S1A) but barely detectable at 14 dpci (Appendix Fig. S1B). To gain further insight into *sulf1* expression dynamics, we developed a *TgBAC(sulf1:EGFP)* line. *sulf1:*EGFP expression was detectable as early as 5 h post-fertilization (hpf) and was subsequently observed in the embryonic head and tail regions (Appendix Fig. S1C), consistent with earlier studies (Gorsi et al, 2010). In uninjured ventricles, *sulf1:*EGFP was detectable in Caveolin1^+^ (Cav1) epicardial cells (Grivas et al, 2020) (Fig. 1E) and Aldh1a2^+^ cells (Fig. 1G). Next, we analyzed *TgBAC(sulf1:EGFP)* ventricular sections at 4 dpci, coinciding with high *sulf1* expression, and co-stained them for Cav1 and Aldh1a2. Consistent with the HCR results, *TgBAC(sulf1:EGFP)* was expressed by EPDCs in the injured area (Fig. 1F,H). Altogether, these data show that EPDCs upregulate *sulf1* expression during cardiac regeneration.

### Abrogation of *sulf1* suppresses epicardial activation after cardiac injury

**Figure 2:**
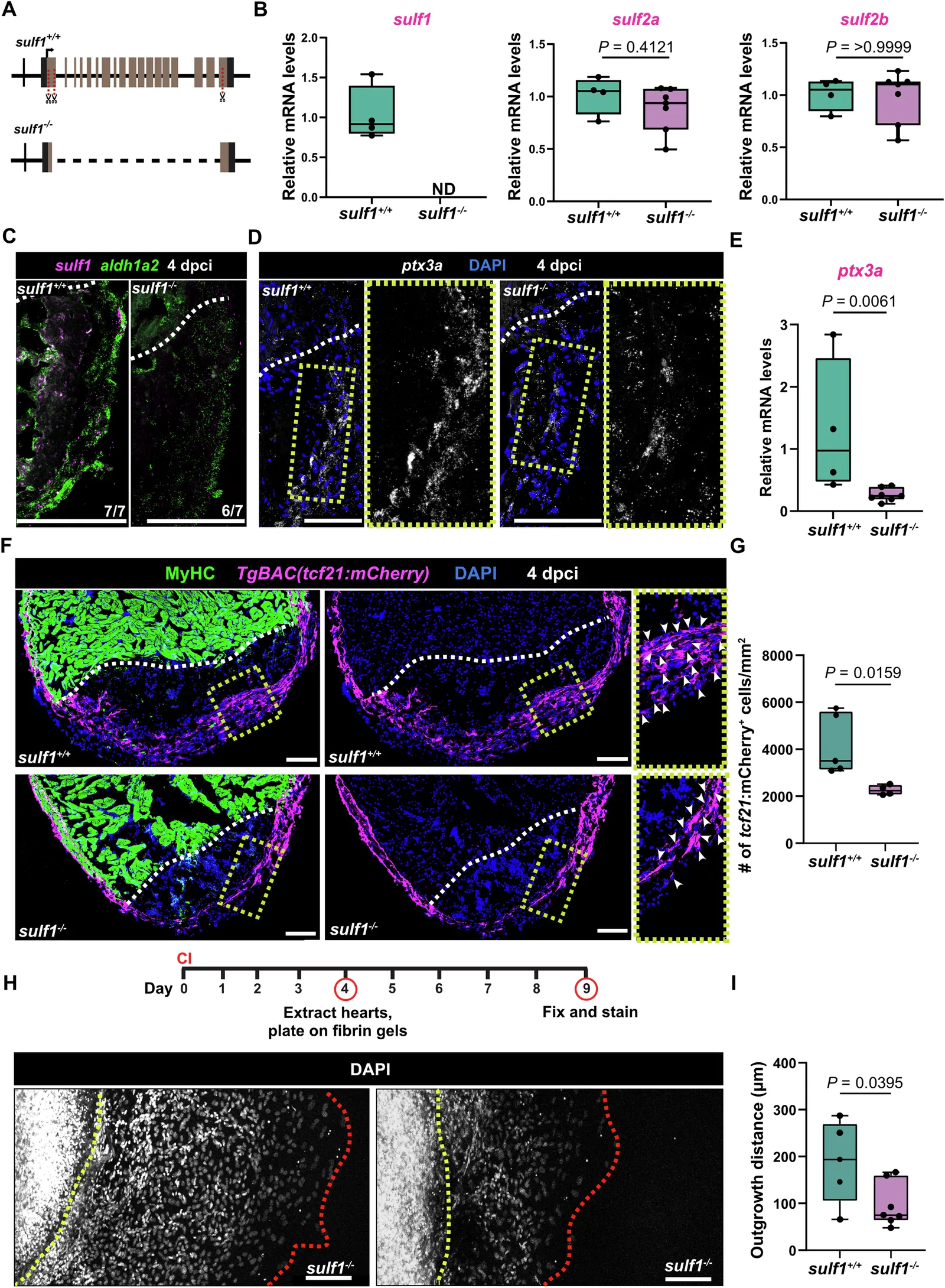
Abrogation of *sulf1* suppresses epicardial activation. (**A**) Schematic representation of the *sulf1* locus showing gRNAs (red dotted lines) targeting exons 2 and 19 to generate a full-locus deletion allele. Exons are shown as brown boxes and UTRs as black boxes. The arrow indicates the transcription start site. The black dashed line represents the deleted sequence. (**B**) RT-qPCR analysis of *sulf1*, *sulf2a* and *sulf2b* mRNA levels in *sulf1*^*+/+*^ (*n* = 4) and *sulf1*^*−/−*^ (*n* = 7) siblings at 5 dpf. (**C**) HCR for *sulf1* (magenta) and *aldh1a2* (green) on *sulf1*^*+/+*^ (*n* = 7) and *sulf1*^*−/−*^ (*n* = 7) ventricular sections at 4 dpci. (**D**) In situ HCR for *ptx3a* (white) and DAPI (DNA, blue) on *sulf1*^*+/+*^ and *sulf1*^*−/−*^ ventricular sections at 4 dpci. Insets show high-magnification images of border zone EPDCs expressing *ptx3a*. (**E**) RT-qPCR analysis of *ptx3a* mRNA levels in *sulf1*^*+/+*^ (*n* = 4) and *sulf1*^*−/−*^ (*n* = 7) cryoinjured ventricles at 4 dpci. (**F**) 4 dpci *sulf1*^*+/+*^ and *sulf1*^*−/−*^ ventricular sections stained for MyHC (CMs, green), mCherry (EPDCs, magenta), and DAPI (DNA, blue). White arrowheads point to *tcf21*:mCherry^+^ cells. White dashed lines delineate the injured area (**G**). Number of *tcf21*:mCherry^+^ cells per mm^2^ injured area in *sulf1*^*+/+*^ (*n* = 5) and *sulf1*^*−/−*^ (*n* = 4) ventricles at 4 dpci. (**H**) Schematic representation of the adult ventricular explant culture protocol following cryoinjury (CI). *sulf1*^*+/+*^ and *sulf1*^*−/−*^ cardiac explants stained for DAPI (DNA, white). Yellow dotted lines delineate plated ventricular explants, and red dotted lines outline the front of the leading edge. (**I**) Outgrowth distance for *sulf1*^*+/+*^ (*n* = 5) and *sulf1*^*−/−*^ (*n* = 7) ventricular explants. Scale bars: 100 µm. Statistical tests: Student’s *t* test (**G**, **I**) and nonparametric Mann-Whitney test (**B**, **E**). For the boxplots, the center line indicates the median; the box bounds represent the 25th and 75th percentiles; the whiskers extend to the most extreme data points; and each point represents an independent measurement. Source data are available online for this figure.

**Figure EV1:**
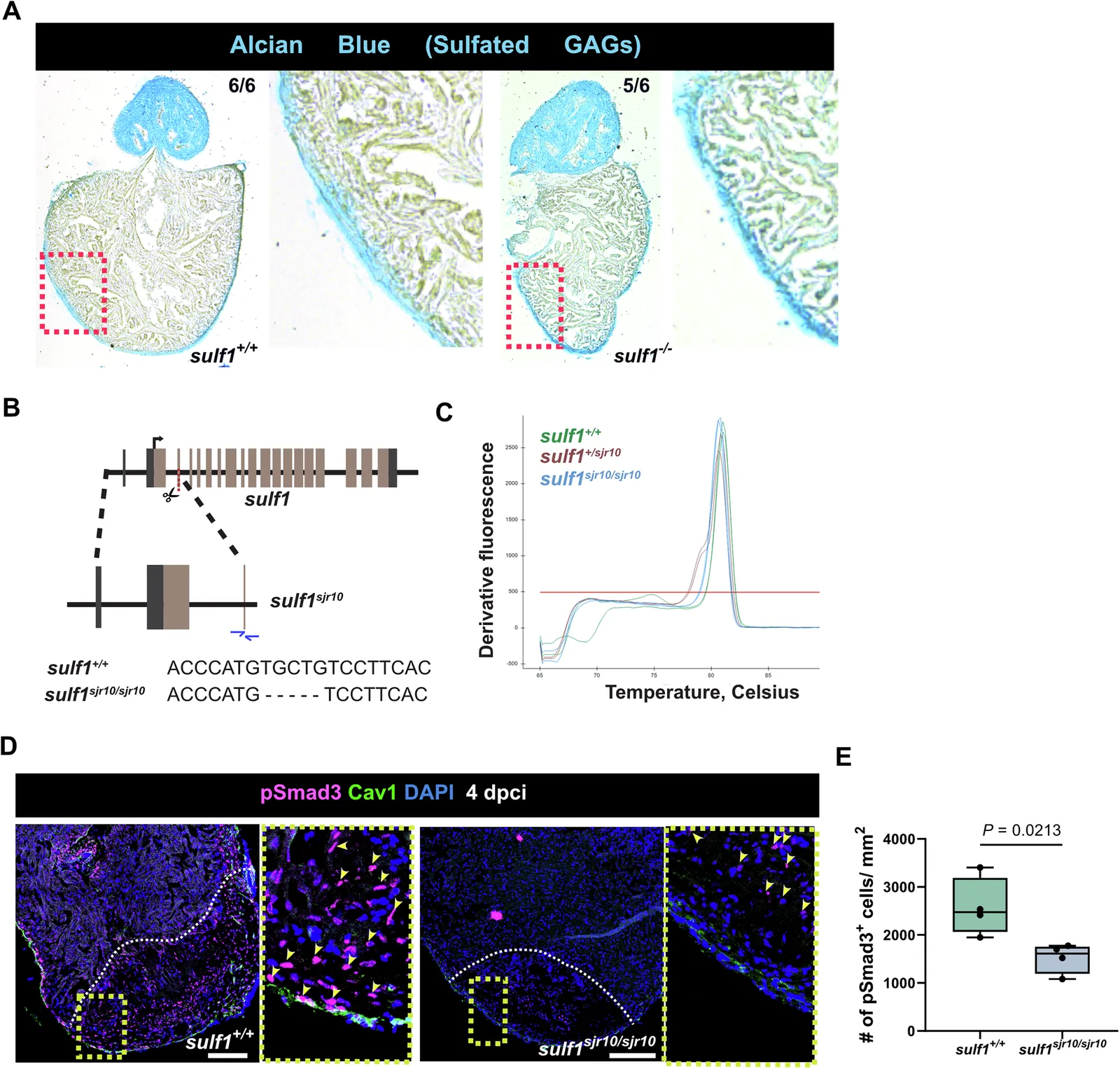
Sulfated glycosaminoglycan (GAG) assessment and pSmad3 levels in *sulf1*^*sjr10*^. (**A**) *sulf1*^*+/+*^ (*n* = 6) and *sulf1*^*−/−*^ (*n* = 6) uninjured ventricular sections stained for Alcian Blue (sulfated GAGs, blue). (**B**) Schematic representation of *sulf1* locus showing gRNA (pink line) targeting exon 3 to generate *sulf1*^*sjr10*^ allele. Exons are shown as brown boxes and UTRs as black boxes. Genotyping primer positions are represented with blue arrows. *sulf1*^*sjr10/sjr10*^ has a 5-nucleotide deletion in the region encoding the sulfatase domain, leading to a premature stop codon. (**C**) High-resolution melt analysis used for genotyping. Melting curves for 2 independent fish of each genotype are shown. (**D**) 4 dpci *sulf1*^*+/+*^ and *sulf1*^*sjr10/sjr10*^ ventricular sections stained for pSmad3 (magenta), Cav1 (EPCDs, green), and DAPI (DNA, blue). Yellow arrowheads point to pSmad3^+^ cells. White dashed lines delineate the injured area. Scale bars: 100 µm. (**E**) Number of pSmad3^+^ cells per mm^2^ injured area in *sulf1*^*+/+*^ (*n* = 4) and *sulf1*^*sjr10/sjr10*^ (*n* = 4) ventricles at 4 dpci. Statistical tests: Student’s *t* test. For the boxplot, the center line indicates the median; the box bounds represent the 25th and 75th percentiles; the whiskers extend to the most extreme data points; and each point represents an independent measurement.

To investigate the function of Sulf1 during cardiac regeneration, we generated a *sulf1* mutant allele using the CRISPR/Cas9 technology. To avoid transcriptional adaptation (El-Brolosy et al, 2019; Rossi et al, 2015) and potential genetic compensation, we generated a full locus deletion allele by deleting the *sulf1* gene body (Fig. 2A; Appendix Fig. S2A,B). *sulf1*^*−/−*^ larvae lacked detectable *sulf1* expression and showed no compensatory upregulation of the paralogs *sulf2a* and *sulf2b*, when compared with wild-type siblings (Fig. 2B). As Sulf1 removes 6-*O* sulfate groups from HS polysaccharides, we set out to test whether the levels of sulfated HS are increased in the epicardium of *sulf1* mutants. We performed Alcian Blue staining (pH ~1.0) to assess glycosaminoglycans (GAGs) sulfation (Bergeron et al, 2011; Cervino et al, 2026) and found an increase in the mutant epicardium compared to wild-type siblings (Fig. EV1A). Given the marked induction of *sulf1* in EPDCs after cardiac injury, we sought to investigate its role in epicardial regeneration. We began by analyzing the expression of *aldh1a2*, a well-established epicardial activation marker (Kikuchi et al, 2011; Lepilina et al, 2006) and found a strong reduction in *aldh1a2* expression in *sulf1*^*−/−*^ ventricles at 4 dpci compared with wild-types (Fig. 2C). In addition, *sulf1* expression was undetectable in mutant ventricles further supporting its abrogation (Fig. 2C). To further assess whether epicardial responses are impaired upon loss of *sulf1*, we analyzed *ptx3a* expression, a marker of a transiently activated epicardial progenitor cell (aEPC) subpopulation (Xia et al, 2022). We found that *ptx3a* upregulation was blunted in *sulf1* mutant ventricles after injury (Fig. 2D,E). Following cardiac injury, EPDCs expand to cover the injured area, where they support regeneration by serving as a source of signaling factors and progenitor cells (Cao and Poss, 2018; Lepilina et al, 2006). To assess alterations in EPDC abundance, we quantified *tcf21*:mCherry^+^ epicardial cells in the injured area at 4 dpci and observed that the number of *tcf21*:mCherry^+^ cells in *sulf1*^*−/−*^ was significantly lower compared with wild-type siblings (Fig. 2F,G). These data led us to hypothesize that EPDC migration is impaired in mutant hearts. To test this possibility, we analyzed epicardial outgrowth in ventricle explant cultures (Cao et al, 2017; Kim et al, 2012). After 5 days of culture, the distance covered by *sulf1* mutant EPDCs from the explants was significantly reduced compared with that of wild-type explants, indicating defective migration (Fig. 2H,I). Overall, these findings show that HSPG sulfation is increased in the mutant epicardium and that *sulf1* is required for epicardial activation and EPDC expansion in response to cardiac injury.

### Loss of *sulf1* disrupts epicardial TGF-β signaling, cardiomyocyte regeneration, and fibrosis resolution

**Figure 3:**
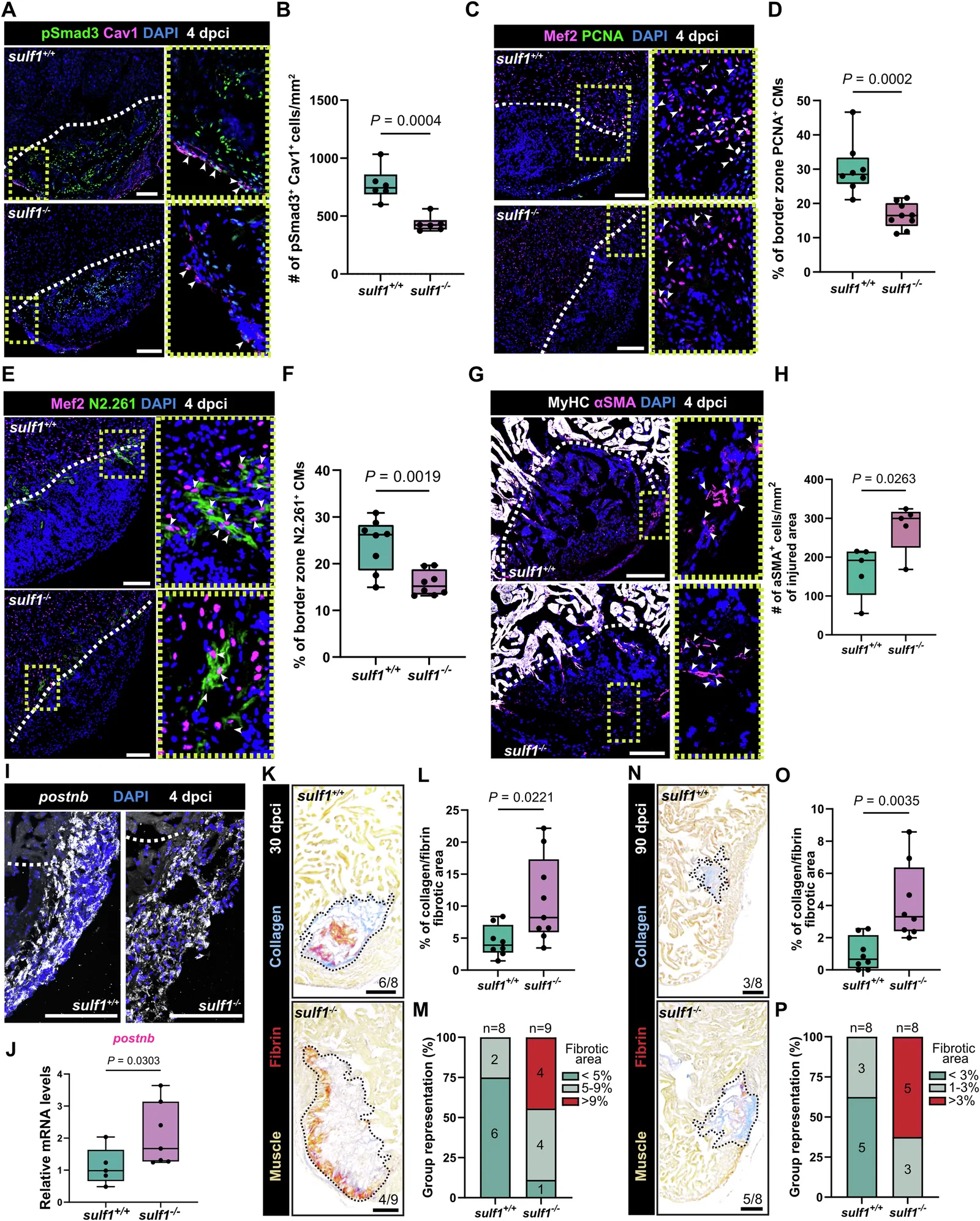
Loss of *sulf1* disrupts epicardial TGF-β signaling, cardiomyocyte regeneration, and fibrosis resolution. (**A**) 4 dpci *sulf1*^*+/+*^ and *sulf1*^*−/−*^ ventricular sections stained for pSmad3 (green), Cav1 (EPDCs, magenta), and DAPI (DNA, blue). Yellow arrowheads point to pSmad3^+^ Cav1^+^ cells, indicating epicardial pSmad3^+^ cells (**B**). Number of pSmad3^+^ epicardial cells (pSmad3^+^ Cav1^+^ cells) per mm^2^ injured area in *sulf1*^*+/+*^ (*n* = 6) and *sulf1*^*−/−*^ (*n* = 6) ventricles at 4 dpci. (**C**) 4 dpci *sulf1*^*+/+*^ and *sulf1*^*−/−*^ ventricular sections stained for Mef2 (CMs, magenta), PCNA (proliferation marker, green), and DAPI (DNA, blue). Arrowheads point to PCNA^+^ CMs. (**D**) Percentage of PCNA^+^ CMs in the injury border zone of ventricular sections of *sulf1*^*+/+*^ (*n* = 8) and *sulf1*^*−/−*^ (*n* = 9) siblings at 4 dpci. (**E**) 4 dpci *sulf1*^*+/+*^ and *sulf1*^*−/−*^ ventricular sections stained for Mef2 (CMs, magenta), N2.261 (embryonic myosin heavy chains, green), and DAPI (DNA, blue). Arrowheads point to N2.261^+^ CMs. (**F**) Percentage of N2.261^+^ CMs in the injury border zone of ventricular sections of *sulf1*^*+/+*^ (*n* = 8) and *sulf1*^*−/−*^ (*n* = 8) siblings at 4 dpci. (**G**) 4 dpci *sulf1*^*+/+*^ and *sulf1*^*−/−*^ ventricular sections stained for MyHC (CMs, white), αSMA (magenta), and DAPI (DNA, blue). Arrowheads point to αSMA^+^ cells. (**H**) Number of αSMA^+^ cells per mm^2^ injured area in *sulf1*^*+/+*^ (*n* = 5) and *sulf1*^*−/−*^ (*n* = 5) ventricular sections at 4 dpci. (**I**) In situ HCR for *postnb* (white) and DAPI (DNA, blue) on *sulf1*^*+/+*^ and *sulf1*^*−/−*^ ventricular sections at 4 dpci. (**J**) RT-qPCR analysis of *postnb* mRNA levels in *sulf1*^*+/+*^ (*n* = 5) and *sulf1*^*−/−*^ (*n* = 7) cryoinjured ventricles at 4 dpci. (**K**) 30 dpci *sulf1*^*+/+*^ and *sulf1*^*−/−*^ ventricular sections stained with AFOG with muscle (yellow), collagen (blue), and fibrin (red). (**L**) Percentage of collagen/fibrin in fibrotic area in *sulf1*^*+/+*^ (*n* = 8) and *sulf1*^*−/−*^ (*n* = 9) ventricles at 30 dpci. (**M**) Percentage representation of groups of different fibrotic area sizes at 30 dpci for cryoinjured *sulf1*^*+/+*^ (*n* = 8) and *sulf1*^*−/−*^ ventricles (*n* = 9). Black dotted lines delineate the area with collagen and fibrin deposits. (**N**) 90 dpci *sulf1*^*+/+*^ and *sulf1*^*−/−*^ ventricular sections stained with AFOG with muscle (yellow), collagen (blue), and fibrin (red). (**O**) Percentage of collagen/fibrin in fibrotic area in *sulf1*^*+/+*^ (*n* = 8) and *sulf1*^*−/−*^ (*n* = 8) ventricles at 90 dpci. (**P**) Percentage representation of groups of different fibrotic area sizes at 90 dpci for cryoinjured *sulf1*^*+/+*^ (*n* = 8) and *sulf1*^*−/−*^ ventricles (*n* = 8). Black dotted lines delineate the area with collagen and fibrin deposits. Scale bars: 100 µm. White dashed lines delineate the injured area. Statistical tests: Student’s *t* test (**B**, **D**, **F**, **H**, **L**, **O**) and nonparametric Mann–Whitney test (**J**). For the boxplots, the center line indicates the median; the box bounds represent the 25th and 75th percentiles; the whiskers extend to the most extreme data points; and each point represents an independent measurement. Source data are available online for this figure.

It has been shown that SULF1 can modulate TGF-β signaling in a context-dependent manner, influencing both developmental processes and pathological conditions such as cancer and fibrosis (Dhanasekaran et al, 2015; Fang et al, 2024; Justo et al, 2022; Meyers et al, 2013). In the context of cardiac regeneration, TGF-β signaling regulates processes essential for an effective regenerative response, including EMT of *ptx3a*^*+*^ aEPCs and their mesenchymal differentiation (Xia et al, 2022). Indeed, we found that *ptx3a* upregulation is impaired in *sulf1* mutant EPDCs after injury (Fig. 2D,E). TGF-β signaling is mediated through a signaling cascade culminating in the phosphorylation and activation of SMAD transcription factors. To determine whether this pathway is altered in injured mutant hearts, we quantified pSmad3 expression and found a significant reduction in pSmad3^+^ EPDCs in *sulf1*^*−/−*^ ventricles at 4 dpci (Fig. 3A,B). This phenotype was recapitulated in *sulf1*^*sjr10/sjr10*^, where the sulfatase domain is mutated (Fig. EV1B–E).

Previous studies have implicated TGF-β/Smad3 signaling in promoting CM regeneration by enabling an EMT-like response in epicardial cells during zebrafish heart regeneration (Chablais and Jaźwińska, 2012; Peng et al, 2021). As epicardial activation and injury coverage are disrupted in *sulf1* mutants, we sought to determine whether these defects compromise CM regeneration. After cardiac injury, CMs dedifferentiate and re-enter the cell cycle to rebuild the lost myocardium (Jopling et al, 2010; Kikuchi et al, 2010). Consistent with the disrupted epicardial response, we found that both CM proliferation and dedifferentiation are significantly reduced in *sulf1*^*−/−*^ ventricles compared with wild-type siblings at 4 dpci (Fig. 3C–F). Together, these findings suggest that loss of *sulf1* perturbs epicardial TGF-β/Smad3 signaling, leading to defective epicardial activation and reduced CM regenerative capacity after injury.

Following cardiac injury, fibroblasts activate and deposit extracellular matrix proteins, leading to the formation of a fibrotic scar. Unlike adult mammals, this fibrotic response is transient in zebrafish (Chablais et al, 2011; Sánchez-Iranzo et al, 2018). Fibrosis is largely driven by TGF-β-mediated differentiation of fibroblasts into α-smooth muscle actin (αSMA)^+^ myofibroblasts. Given that disruptions in TGF-β signaling have been linked to an impaired fibrotic response (Frangogiannis, 2020; Massagué and Sheppard, 2023; Midgley et al, 2013; Sánchez-Iranzo et al, 2018; Shi et al, 2020), we reasoned that the reduced TGF-β signaling observed in *sulf1* mutants might compromise fibrosis. To test this possibility, we quantified the number of αSMA^+^ myofibroblasts in the injured area. At 4 dpci, we found an increased abundance of αSMA^+^ cells in *sulf1*^*−/−*^ ventricles compared with wild-type siblings (Fig. 3G,H). To further assess the myofibroblast phenotype, we analyzed *postnb* expression, a marker of activated fibroblasts. We found that *postnb* expression was significantly higher in *sulf1* mutant ventricles after injury (Fig. 3I,J). Prolonged or elevated myofibroblast activity can result in excessive deposition of collagen and other extracellular matrix components, ultimately contributing to the development of fibrotic tissue. Thus, to assess fibrosis in cryoinjured *sulf1*^*-/-*^ ventricles, we performed acid fuchsin orange G (AFOG) staining, marking collagen, fibrin, and muscle. At 30 dpci, *sulf1*^*−/−*^ ventricles exhibited increased fibrosis as denoted by larger collagen/fibrin deposits compared with wild-type siblings (Fig. 3K–M). To further assess fibrosis resolution at a later timepoint, we analyzed ventricles at 90 dpci, when regeneration is largely complete in wild-type hearts, and found that mutant ventricles presented larger collagen/fibrin deposits, indicating persistent fibrosis (Fig. 3N–P).

Collectively, these results support that Sulf1 regulates TGF-β signaling-mediated epicardial activation and EPDC regeneration, both required for efficient fibrosis resolution.

### *sulf1* mutants display alterations in injury-activated epicardial subpopulations

**Figure 4:**
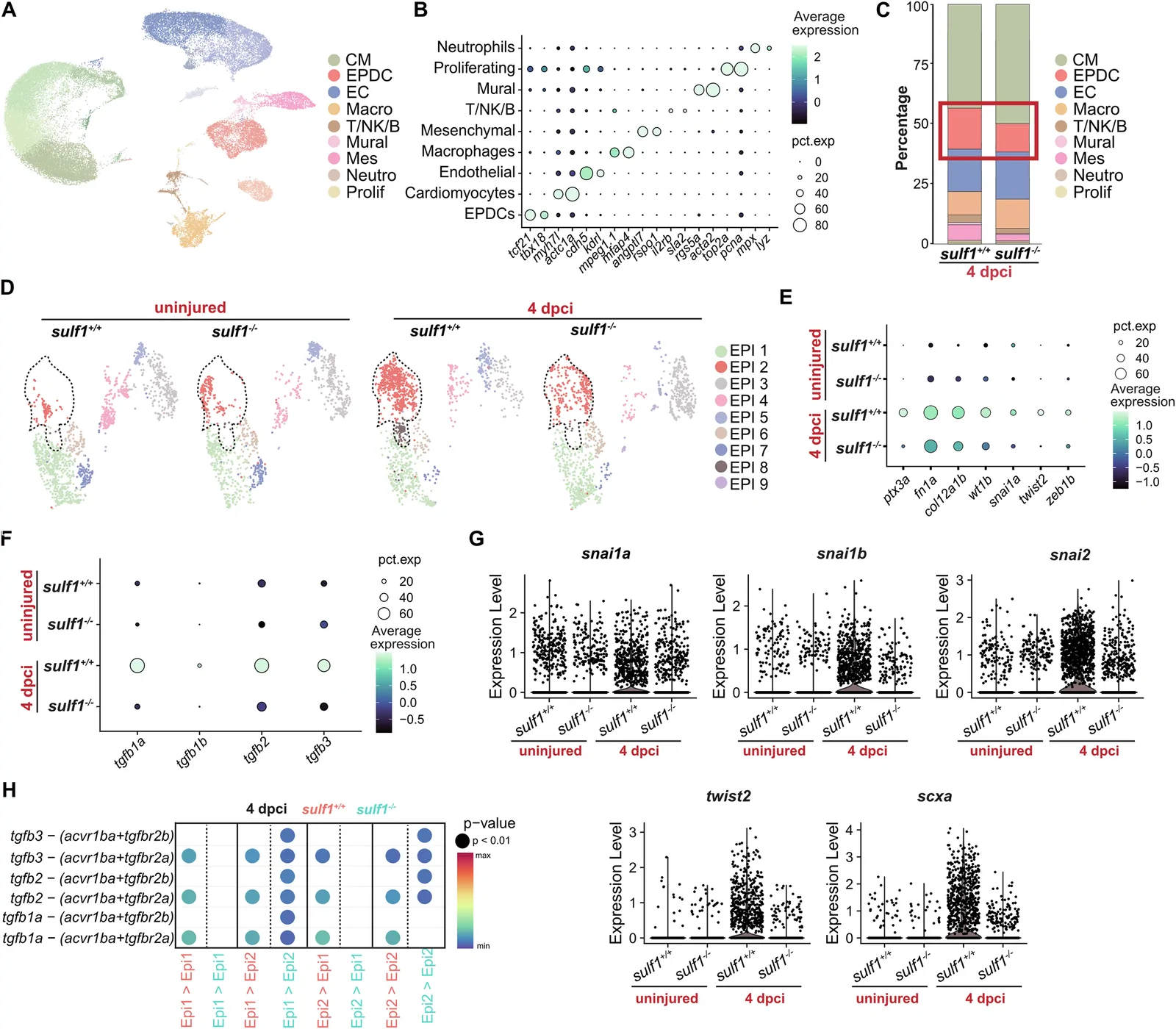
*sulf1* mutants display alterations in injury-activated epicardial subpopulations and TGF-β signaling. (**A**) UMAP representation of all samples combined and with inferred cellular identity after scRNA-seq analysis. (**B**) Dot plot depicting average expression and abundance (pct. exp) of marker genes across all cell types. (**C**) Proportion of cells per cluster within each sample at 4 dpci. The red box shows the proportion of EPDCs. (**D**) UMAP representation of EPDCs subclusters of individual groups from the scRNA-seq analysis. Black dashed lines demarcate activated EPDCs clusters. (**E**) Dot plot depicting average expression and abundance (pct. exp) of *ptx3a, fn1a, col12a1b, wt1b, snai1a, twist2*, and *zeb1b* in EPDCs of individual groups. (**F**) Dot plot depicting average expression and abundance (pct. exp) of *tgfb1a, tgfb1b, tgfb2*, and *tgfb3* in EPDCs of individual groups. (**G**) Violin plots showing expression of *snai1a, snai1b, snai2, twist2*, and *scxa* in EPDCs of individual groups. (**H**) Dot plot depicting communication patterns of TGF-β signaling in epicardial cells in *sulf1*^*+/+*^ and *sulf1*^*−/−*^ 4 dpci groups. The dot color and size represent the calculated communication probability and *P* values, respectively. Statistical test: Cell Chat (Permutation test) (Jin et al, 2021).

**Figure EV2:**
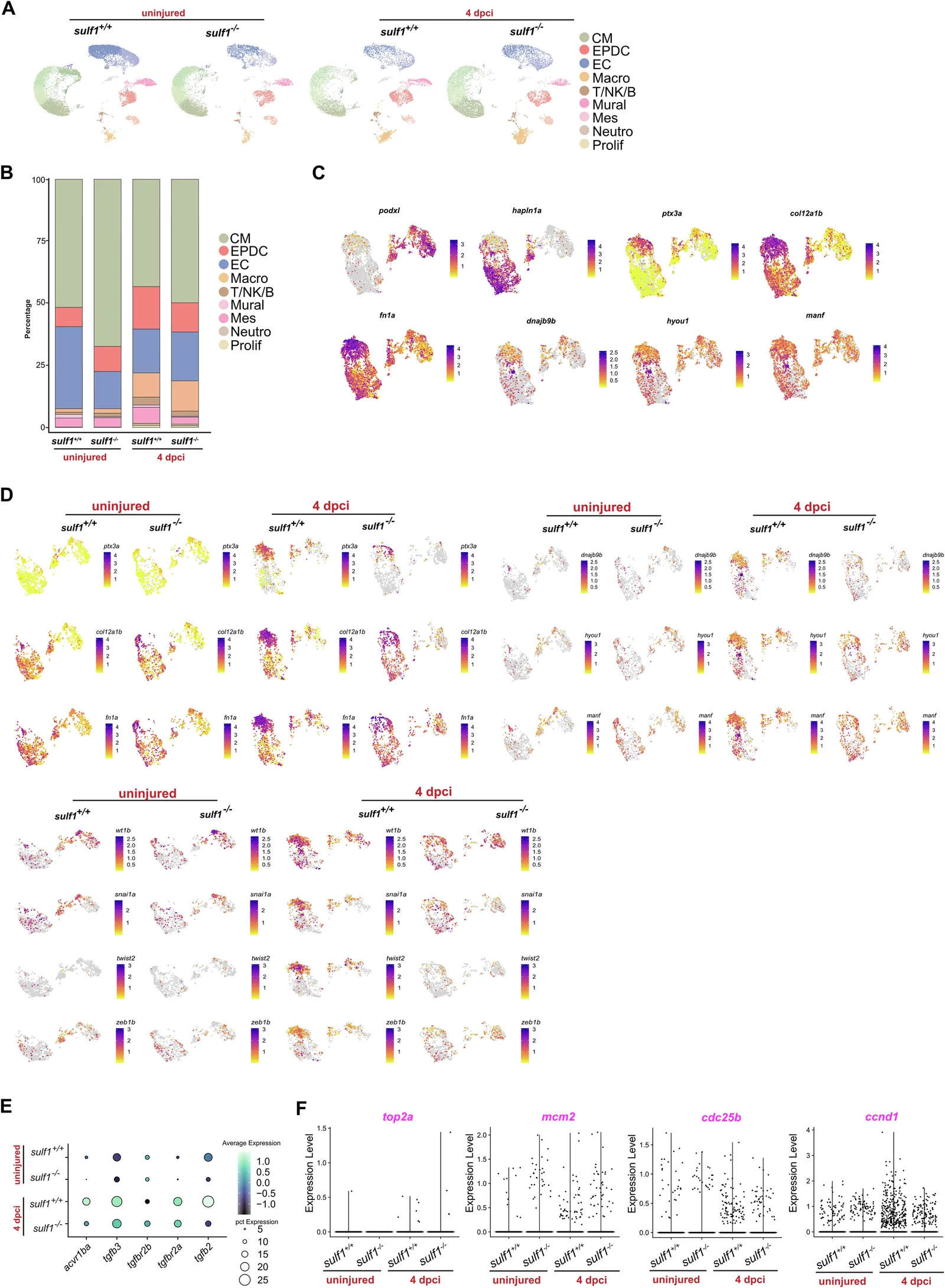
Transcriptional changes in EPDCs subclusters of *sulf1*^*−/−*^ mutants. (**A**) UMAP representation of individual groups depicting cluster changes between all groups. (**B**) Proportion of cells per cluster within each sample. (**C**) UMAPs showing normalized expression of marker genes of epithelial EPDCs (*podxl*), mesenchymal EPDCs (*hapln1a*), genes enriched in EPI 2 cluster (*ptx3a, col12a1b, fn1a),* and genes enriched in EPI 8 cluster (*dnajb9b, hyou1, manf*) in all samples combined. (**D**) UMAPs showing normalized expression of *ptx3a, col12a1b, fn1a, dnajb9b, hyou1, manf*, *wt1b, snai1a, twist2* and *zeb1b* in individual groups. (**E**) Dot plot depicting average expression and abundance (pct. exp) of *acvr1ba, tgfb3, tgfbr2b, tgfbr2a*, and *tgfb2* in EPDCs of individual groups. (**F**) Violin plots showing expression of *top2a*, *mcm2, cdc25b*, and *ccnd1* in EPDCs of individual groups.

To dissect the impact of *sulf1* abrogation on distinct cardiac cell populations during regeneration, we performed scRNA-seq on ventricles from *sulf1*^*−/−*^ and wild-type siblings. These analyses were performed at 4 dpci, when the expression of *sulf1* is high and early phenotypes are observed, providing a relevant window to capture the transcriptional changes associated with the loss of *sulf1*. Based on the expression of established marker genes, we identified nine cell populations: CMs, EPDCs, endothelial cells (ECs), macrophages, neutrophils, lymphocytes (T/NK/B cells), mural cells, and mesenchymal cells. Also, a small cluster exhibiting high expression of proliferation-related genes was identified and annotated as proliferating cells (Figs. 4A,B and  EV2A).

First, to assess injury-induced changes across different cell types, we analyzed the proportion of each cell type within each sample. Quantitative analysis of cell-type proportions revealed a significant decrease in EPDCs in *sulf1* mutants after injury (Figs. 4C and  EV2B), consistent with previous observations of impaired epicardial activation (Fig. 2C–I). To further investigate the defects in EPDCs and epicardial activation, we performed subclustering analyses of EPDCs across all samples, which resolved eight distinct subclusters (Fig. 4D). Consistent with previously reported epicardial subsets (Xia et al, 2022), our dataset also robustly captured both *podxl*⁺ epithelial EPDCs (EPI 3 and EPI 5) and *hapln1a*⁺ mesenchymal EPDCs (EPI 1, EPI 6, and EPI 7) (Fig. EV2C). Two clusters, EPI 2 and EPI 8, were found to be specifically enriched in 4 dpci samples and were accordingly annotated as injury-induced epicardial populations. EPI 2 was characterized by the expression of *ptx3a*, *col12a1b*, and *fn1a* (Fig. EV2C), all previously identified as markers of transient aEPCs (Xia et al, 2022). As recently shown (Kayman Kürekçi et al, 2026), EPI 8 showed elevated expression of *dnajb9b*, *hyou1*, and *manf*, which are typically upregulated in response to cellular stress conditions such as hypoxia and endoplasmic reticulum stress (Glembotski et al, 2012; Lee et al, 2015; Rao et al, 2021; Yu et al, 2021) (Fig. EV2C).

To assess alterations in the epicardial response to injury, we compared the injury-induced epicardial populations between *sulf1*^*−/−*^ and wild-type siblings at 4 dpci. We observed pronounced differences between mutant and wild-type populations, including a marked reduction of the EPI 2 cluster and the complete absence of the EPI 8 cluster in *sulf1*^*−/−*^ hearts after injury (Fig. 4D), further supporting that Sulf1 regulates epicardial activation (Fig. 2C–I). Impaired epicardial activation was further evidenced by reduced expression of epicardial activation markers (*ptx3a, fn1a, col12a1b*) (Xia et al, 2022) and EMT markers (*wt1b, snai1a, twist2, zeb1b*) (Ansieau et al, 2008; Duim et al, 2016; Fang et al, 2011; Gentile et al, 2021; Wagner and Wagner, 2021; Zhang et al, 2015) in mutant hearts (Figs. 4E and  EV2D). In addition, reduced expression of cell cycle regulators *cdc25b* and *ccnd1* in mutants suggests alterations in EPDC proliferation (Fig. EV2F).

Building on the observation of diminished TGF-β signaling in mutant ventricles (Fig. 3A,B), we interrogated our scRNA-seq dataset to dissect this pathway in greater detail. Expression of all TGF-β ligands (*tgfb1a, tgfb1b, tgfb2*, and *tgfb3*) was reduced in mutant EPDCs (Fig. 4F), along with the expression of downstream TGF-β target genes (*snai1a, snai1b, snai2, twist2, scxa*) (Xu et al, 2009) (Fig. 4G). To quantitatively assess and analyze intercellular communication networks among EPDCs, we performed CellChat analyses comparing mutant and wild-type EPDCs clusters in the whole dataset (Fig. 4A) (Jin et al, 2021). This analysis revealed an alteration of TGF-β signaling, including the loss of canonical TGF-β-associated interactions and the emergence of additional interactions in mutant EPDCs involving *tgfbr2b*. These interactions likely arise because *tgfbr2b* has higher expression in the mutant EPDCs, enabling ligand–receptor interaction in the mutant (Figs. 4H and  EV2E).

Together, these analyses consistently indicate disrupted epicardial activation coupled with altered TGF-β signaling in the *sulf1* mutants.

### Sulf1-dependent TGF-β signaling controls inflammation after cardiac injury

**Figure 5:**
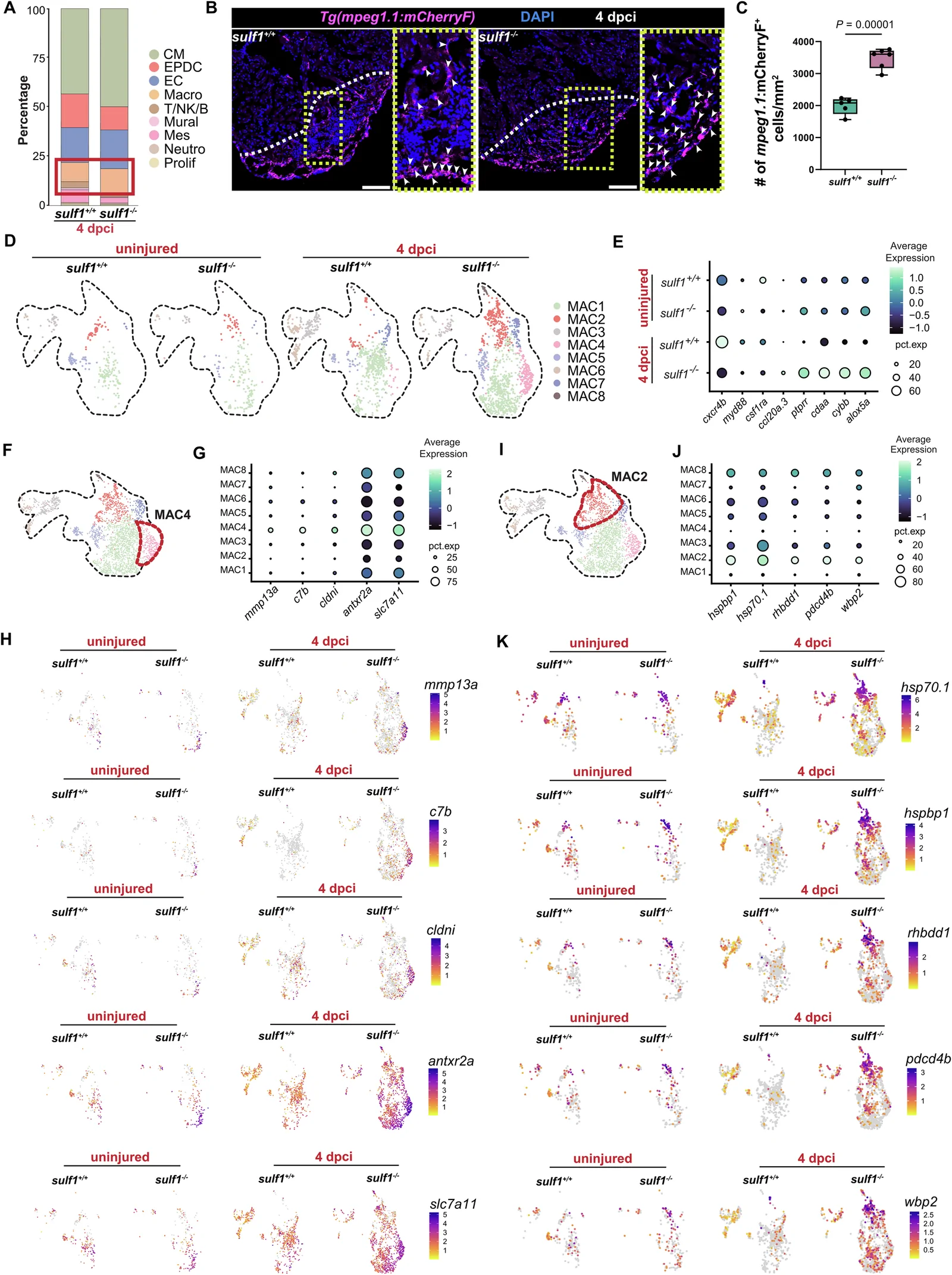
Loss of *sulf1* alters the macrophage response after cardiac injury. (**A**) Proportion of cells per cluster within each sample at 4 dpci, red box shows macrophage’s proportion. (**B**) 4 dpci *sulf1*^*+/+*^ and *sulf1*^*−/−*^ ventricular sections stained for mCherry (macrophages, magenta) and DAPI (DNA, blue). Arrowheads point to *mpeg1.1*:mCherryF^+^ cells. (**C**) Number of *mpeg1.1*:mCherryF^+^ cells per mm^2^ injured area in *sulf1*^*+/+*^ (*n* = 5) and *sulf1*^*−/−*^ (*n* = 6) ventricles at 4 dpci. White dashed lines delineate the injured area. (**D**) UMAP representation of macrophage subclusters of individual groups from the scRNA-seq analysis. (**E**) Dot plot depicting average expression and abundance (pct. exp) of *cxcr4b, myd88, csf1ra, ccl20a.3, ptprr, cdaa, cybb*, and *alox5a* in macrophages of individual groups. (**F**) UMAP representation of macrophage subclusters of all samples combined, red dotted line highlighting MAC4 cluster. (**G**) Dot plot depicting average expression and abundance (pct. exp) of *mmp13a, c7b, cldni, antxr2a*, and *slc7a11* in all macrophage subclusters. (**H**) Normalized expression of *mmp13a, c7b, cldni, antxr2a*, and *slc7a11* in macrophage UMAPs of individual groups. (**I**) UMAP representation of macrophage subclusters of all samples combined, red dotted line highlighting MAC2 cluster. (**J**) Dot plot depicting average expression and abundance (pct. exp) of *hspbp1, hsp70.1, rhbdd1, pdcd4b*, and *wbp2* in all macrophage subclusters. (**K**) Normalized expression of *hspbp1, hsp70.1, rhbdd1, pdcd4b*, and *wbp2* in macrophage UMAPs of individual groups. Scale bars: 100 µm. Statistical tests: Student’s *t* test (**C**). For the boxplot, the center line indicates the median; the box bounds represent the 25th and 75th percentiles; the whiskers extend to the most extreme data points; and each point represents an independent measurement. Source data are available online for this figure.

**Figure EV3:**
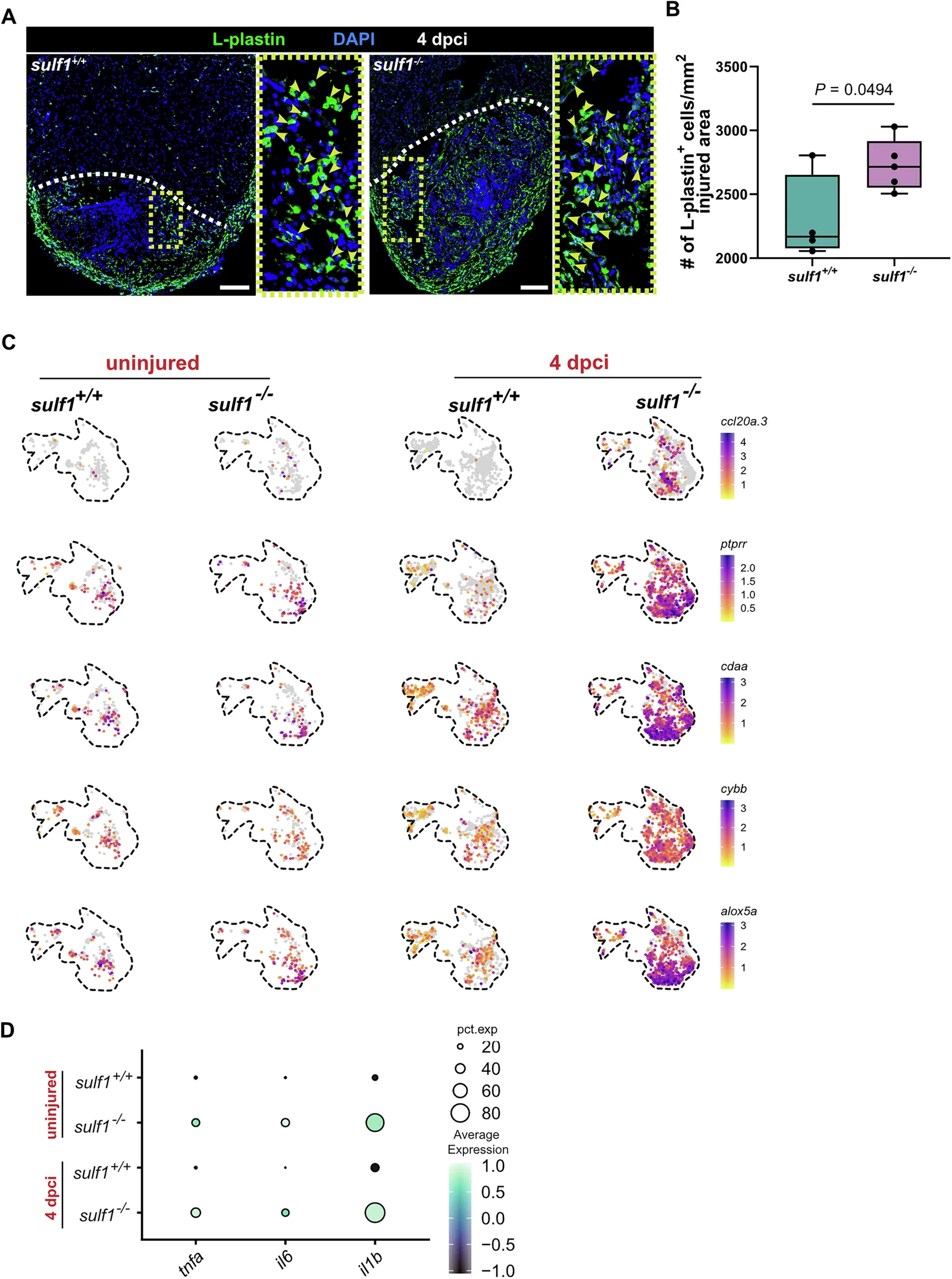
Transcriptional changes in macrophage subclusters of *sulf1*^*−/−*^ mutants. (**A**) 4 dpci *sulf1*^*+/+*^ and *sulf1*^*-/-*^ ventricular sections stained for L-plastin (leukocytes, green) and DAPI (DNA, blue). Arrowheads point to L-plastin^+^ cells. White dashed lines delineate the injured area. Scale bars: 100 µm. (**B**) Number of L-plastin^+^ cells per mm^2^ injured area in *sulf1*^*+/+*^ (*n* = 4) and *sulf1*^*−/−*^ (*n* = 5) ventricles at 4 dpci. Statistical test: Student’s *t* test. (**C**) UMAPs showing the normalized expression of *ccl20a.3, ptprr, cdaa, cybb*, and *alox5a* in macrophages of individual groups. (**D**) Dot plot depicting average expression and abundance (pct. exp) of *tnfa, il6*, and *il1b* in macrophages of individual groups.

Our transcriptomic analyses show an increased proportion of macrophages in the injured mutant hearts (Fig. 5A), prompting us to further investigate the inflammatory response. We began by analyzing macrophage numbers in the injured area of *sulf1* mutants by immunostaining for *mpeg1.1*:mCherryF^+^ macrophages at 4 dpci. *sulf1*^*−/−*^ ventricles displayed a significantly higher number of *mpeg1.1*:mCherryF^+^ macrophages compared with wild-type siblings (Fig. 5B,C). Notably, the total leukocyte infiltration in the mutant was also significantly increased (Fig. EV3A,B). Building on these observations, we used our scRNA-seq dataset to further investigate this phenotype. We performed subclustering analysis of macrophages across all samples and identified eight transcriptionally distinct populations (Fig. 5D). Comparative analyses revealed that macrophages in mutant ventricles exhibit a transcriptional program distinct from that of wild-type macrophages at 4 dpci (Fig. 5D–K), with downregulation of the wild-type injury response (Denans et al, 2022; Goumenaki et al, 2024; Hason et al, 2022) (Fig. 5E) and upregulation of inflammatory genes unique to mutant macrophages (Figs. 5E and  EV3C). Among the most upregulated genes in *sulf1*^*−/−*^ macrophages at 4 dpci were *ccl20a.3, ptprr, cdaa, cybb*, and *alox5a*, all encoding proteins involved in inflammation, either by inducing pro-inflammatory mediators such as TNF-α, modulating macrophage polarization, or regulating pathways involved in inflammation (Keller et al, 2021; Lv et al, 2023; Miura et al, 2012; Song et al, 2024; Ye et al, 2022; Zhao et al, 2014) (Figs. 5E and  EV3C).

Further analyzing the transcriptional differences observed in macrophages, we identified two injury-responsive macrophage clusters, MAC 2 and MAC 4, that were uniquely expanded in mutants (Fig. 5F–K). The top upregulated genes in the MAC 4 cluster were *mmp13a, c7b, cldni, antxr2a*, and *slc7a11*, all associated either with ECM remodeling and degradation or macrophage activity during tissue homeostasis and disease (Hu and Ecker, 2021; Huang et al, 2024; Massri et al, 2024; Yang et al, 2024) (Fig. 5F–H). In contrast, the MAC 2 cluster showed upregulation of *rhbdd1, pdcd4b*, and *wbp2* along with several genes encoding heat-shock proteins (e.g., *hsp70.1, hspbp1*). These genes are known to play roles in macrophage differentiation and alternative activation (Fagone et al, 2012; Ren et al, 2013; Smith et al, 2010; Tabatabaeian et al, 2020; Zhong et al, 2014) (Fig. 5I–K).

**Figure 6:**
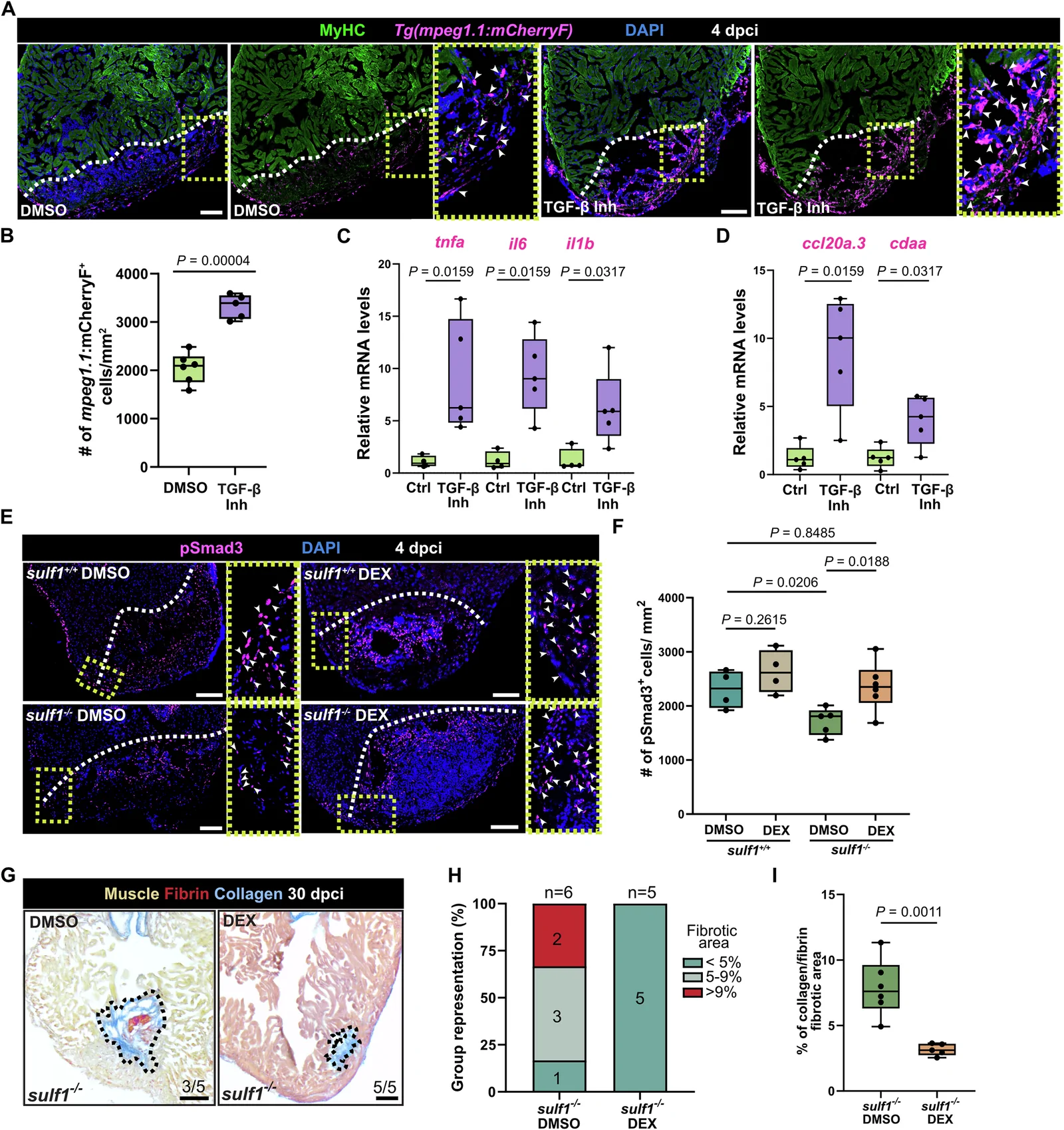
Sulf1-dependent TGF-β signaling controls inflammation after cardiac injury. (**A**) 4 dpci DMSO-treated and TGF-β inhibitor-treated ventricular sections stained for MyHC (CMs, green), mCherry (macrophages, magenta), and DAPI (DNA, blue). Arrowheads point to *mpeg1.1*:mCherryF^+^ cells. (**B**) Number of *mpeg1.1*:mCherryF^+^ cells per mm^2^ injured area in DMSO-treated (*n* = 6) and TGFβ inhibitor-treated (*n* = 5) ventricles at 4 dpci. (**C**) RT-qPCR analysis of *tnfa, il6* and *il1b* mRNA levels in control (Ctrl) (*n* = 4) and TGF-β inhibitor-treated (*n* = 5) cryoinjured ventricles at 4 dpci. (**D**) RT-qPCR analysis of *ccl20a.3* and *cdaa* mRNA levels in control (Ctrl) (*n* = 5) and TGFβ inhibitor-treated (*n* = 5) cryoinjured ventricles at 4 dpci. (**E**) 4 dpci *sulf1*^*+/+*^, *sulf1*^*+/+*^ dexamethasone-treated (DEX), *sulf1*^*−/−*^ and *sulf1*^*−/−*^ dexamethasone-treated ventricular sections stained for pSmad3 (magenta) and DAPI (DNA, blue). Arrowheads point to pSmad3^+^ cells. (**F**) Number of pSmad3^+^ cells per mm^2^ injured area in *sulf1*^*+/+*^ (*n* = 4), *sulf1*^*+/+*^ dexamethasone-treated (*n* = 4), *sulf1*^*−/−*^ (*n* = 5) and *sulf1*^*−/−*^ dexamethasone-treated (*n* = 6) ventricles at 4 dpci. (**G**) 30 dpci *sulf1*^*−/−*^ DMSO-treated and *sulf1*^*−/−*^ dexamethasone-treated ventricular sections stained with AFOG for muscle (yellow), collagen (blue), and fibrin (red). (**H**) Percentage representation of groups of different fibrotic area sizes at 30 dpci for cryoinjured *sulf1*^*−/−*^ DMSO-treated (*n* = 6) and *sulf1*^*−/−*^ dexamethasone-treated (*n* = 5) ventricles. Black dotted lines delineate the area with collagen and fibrin deposits. (**I**) Percentage of collagen/fibrin in fibrotic area in *sulf1*^*−/−*^ DMSO-treated (*n* = 6) and *sulf1*^*−/−*^ dexamethasone-treated (*n* = 5) ventricles at 30 dpci. Scale bars: 100 µm. White dashed lines delineate the injured area. Statistical tests: Nonparametric Mann–Whitney test (**C**, **D**), Student’s *t* test (**B**, **F**, **I**). For the boxplots, the center line indicates the median; the box bounds represent the 25th and 75th percentiles; the whiskers extend to the most extreme data points; and each point represents an independent measurement. Source data are available online for this figure.

Previous studies support a regulatory interplay between TGF-β signaling and macrophage polarization (Zhang et al, 2016). We therefore sought to determine whether the defects in TGF-β signaling and the increased inflammatory response observed in *sulf1* mutants are functionally linked. To investigate whether the inflammation observed in *sulf1*^*−/−*^ could be attributed to disrupted TGF-β signaling, we inhibited pharmacologically the TGF-β pathway in wild-type fish. Interestingly, inhibition of TGF-β signaling led to an accumulation of *mpeg1.1*:mCherryF^+^ macrophages prominently in the injury periphery, where EPDCs are localized (Fig. 6A,B). Next, we set out to assess whether inhibition of TGF-β signaling contributes to increased inflammation. To this end, we analyzed the expression of pro-inflammatory factors that were upregulated in mutant macrophages (*tnfa*, *il6, il1b*) and observed a marked upregulation of these genes in TGF-β-inhibited ventricles, mirroring the *sulf1*^*−/−*^ phenotype (Figs. 6C and  EV3D). Next, we assessed whether the inflammatory response elicited by TGF-β signaling blockage recapitulates the transcriptional signature observed in *sulf1* mutant ventricles. Interestingly, we found that inhibition of TGF-β signaling leads to upregulation of *ccl20a.3* and *cdaa* expression, consistent with the *sulf1* mutant phenotype (Figs. 5E and 6D). Overall, these results indicate that Sulf1 modulates the macrophage response to injury via TGF-β signaling regulation.

Next, we asked whether modulating the inflammatory response might impact TGF-β signaling. To test this possibility, we pharmacologically dampened inflammation (Ma et al, 2021) in *sulf1*^*−/−*^ and wild-type siblings and quantified pSmad3 expression in injured ventricles. We found that dexamethasone treatment in *sulf1*^*−/−*^ results in restoration of pSmad3^+^ cells to wild-type levels. In contrast, no significant difference in pSmad3^+^ cells was observed in treated wild-type ventricles (Fig. 6E,F). Building on this observation, we sought to determine whether using this approach to reduce inflammation helps restore fibrosis resolution in *sulf1*^*−/−*^. Indeed, we found that dexamethasone-treated mutant ventricles had significantly smaller collagen/fibrin deposits at 30 dpci compared with DMSO-treated mutant hearts (Fig. 6G–I).

### Epicardial overexpression of *sulf1* stimulates TGF-β signaling and attenuates inflammation

Our results support a model in which Sulf1 activity in the activated epicardium modulates cardiac inflammation by regulating TGF-β-dependent macrophage recruitment.

**Figure 7:**
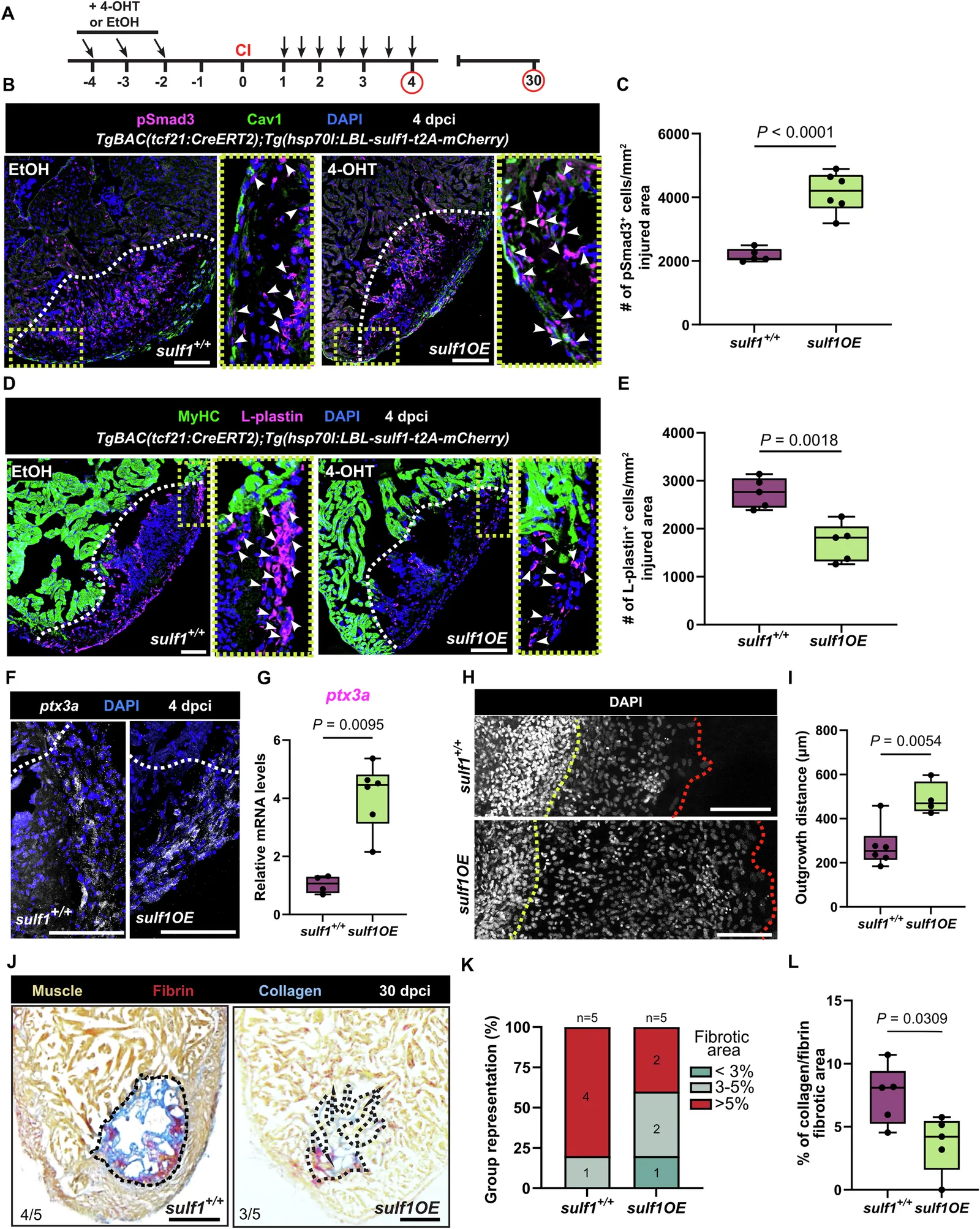
Epicardial overexpression of *sulf1* stimulates TGF-β signaling and attenuates inflammation. (**A**) Schematic representation of 4-hydroxytamoxifen (4-OHT) or ethanol (EtOH) treatments followed by cardiac cryoinjury (CI) and heat-shock treatments (arrows) in *sulf1*^*+/+*^ and *sulf1OE* ventricles. (**B**) 4 dpci *sulf1*^*+/+*^ and *sulf1OE* ventricular sections stained for pSmad3 (magenta), Cav1 (EPCDs, green), and DAPI (DNA, blue). White arrowheads point to pSmad3^+^ cells. (**C**) Number of pSmad3^+^ cells per mm^2^ injured area in *sulf1*^*+/+*^ (*n* = 5) and *sulf1OE* (*n* = 6) ventricles at 4 dpci. (**D**) 4 dpci *sulf1*^*+/+*^ and *sulf1OE* ventricular sections stained for MyHC (CMs, green), L-plastin (leukocytes, magenta), and DAPI (DNA, blue). Arrowheads point to L-plastin^+^ cells. White dashed lines delineate the injured area. (**E**) Number of L-plastin^+^ cells per mm^2^ injured area in *sulf1*^*+/+*^ (*n* = 5) and *sulf1OE* (*n* = 5) ventricles at 4 dpci. (**F**) In situ HCR for *ptx3a* (white) and DAPI (DNA, blue) on *sulf1*^*+/+*^ and *sulf1OE* ventricular sections at 4 dpci showing border zone cells expressing *ptx3a*. (**G**) RT-qPCR analysis of *ptx3a* mRNA levels in *sulf1*^*+/+*^ (*n* = 4) and *sulf1OE* (*n* = 6) cryoinjured ventricles at 4 dpci. (**H**) *sulf1*^*+/+*^ and *sulf1OE* cardiac explants stained for DAPI (DNA, white). The yellow dotted line delineates the plated ventricular explant, and the red dotted line outlines the front of the leading edge. (**I**) Outgrowth distance for *sulf1*^*+/+*^ (*n* = 6) and *sulf1OE* (*n* = 4) ventricular explants. (**J**) 30 dpci *sulf1*^*+/+*^ and *sulf1OE* ventricular sections stained with AFOG for muscle (yellow), collagen (blue), and fibrin (red). (**K**) Percentage representation of groups of different fibrotic area sizes at 30 dpci for cryoinjured *sulf1*^*+/+*^ (*n* = 5) and *sulf1OE* ventricles (*n* = 5). Black dotted lines delineate the area with collagen and fibrin deposits. (**L**) Percentage of collagen/fibrin in fibrotic area in *sulf1*^*+/+*^ (*n* = 5) and *sulf1OE* (*n* = 5) ventricles at 30 dpci. Scale bars: 100 µm. Statistical tests: Student’s *t* test (**C**, **E**, **I**, **L**) and nonparametric Mann–Whitney test (**G**). For the boxplots, the center line indicates the median; the box bounds represent the 25th and 75th percentiles; the whiskers extend to the most extreme data points; and each point represents an independent measurement. Source data are available online for this figure.

Next, we generated a *sulf1* overexpression (*sulf1OE*) line to assess the impact of increased Sulf1 on epicardial activation and inflammatory responses. To recapitulate the dynamic and tissue-specific expression of *sulf1* after cardiac injury, we employed the HOTCre system (Hesselson et al, 2009). We developed a heat-shock inducible *Tg(hsp70l:LBL-sulf1-t2A-mCherry)* overexpression line which in combination with the *TgBAC(tcf21:CreERT2)* line (Kikuchi et al, 2011) enabled temporal and spatial control of *sulf1* induction in EPDCs (Fig. 7A; Appendix Fig. S3A–D). First, we tested whether *sulf1* overexpression could modulate TGF-β signaling and inflammation. Notably, induction of *sulf1* in EPDCs led to a significant increase in pSmad3⁺ cells within the injured area at 4 dpci (Fig. 7B,C). Furthermore, EPDC-specific *sulf1* overexpression resulted in a marked reduction of L-plastin⁺ cells in the injury at 4 dpci, indicating reduced inflammation (Fig. 7D,E). Interestingly, EPDC-specific *sulf1* overexpression ventricles showed elevated *ptx3a* expression, indicating stimulation of epicardial activation and aEPCs expansion (Fig. 7F,G). In addition, *sulf1* overexpression in EPDCs stimulated epicardial outgrowth ex vivo (Fig. 7H,I). Given the observation that EPDC-specific *sulf1* overexpression enhances early pro-regenerative processes, we asked whether it might also modulate fibrosis resolution. To this end, we overexpressed *sulf1* in EPDCs early after injury and analyzed the fibrotic area at 30 dpci. Notably, *sulf1OE* fish exhibited significantly smaller collagen/fibrin deposits compared with wild-type siblings (Fig. 7J–L).

**Figure 8:**
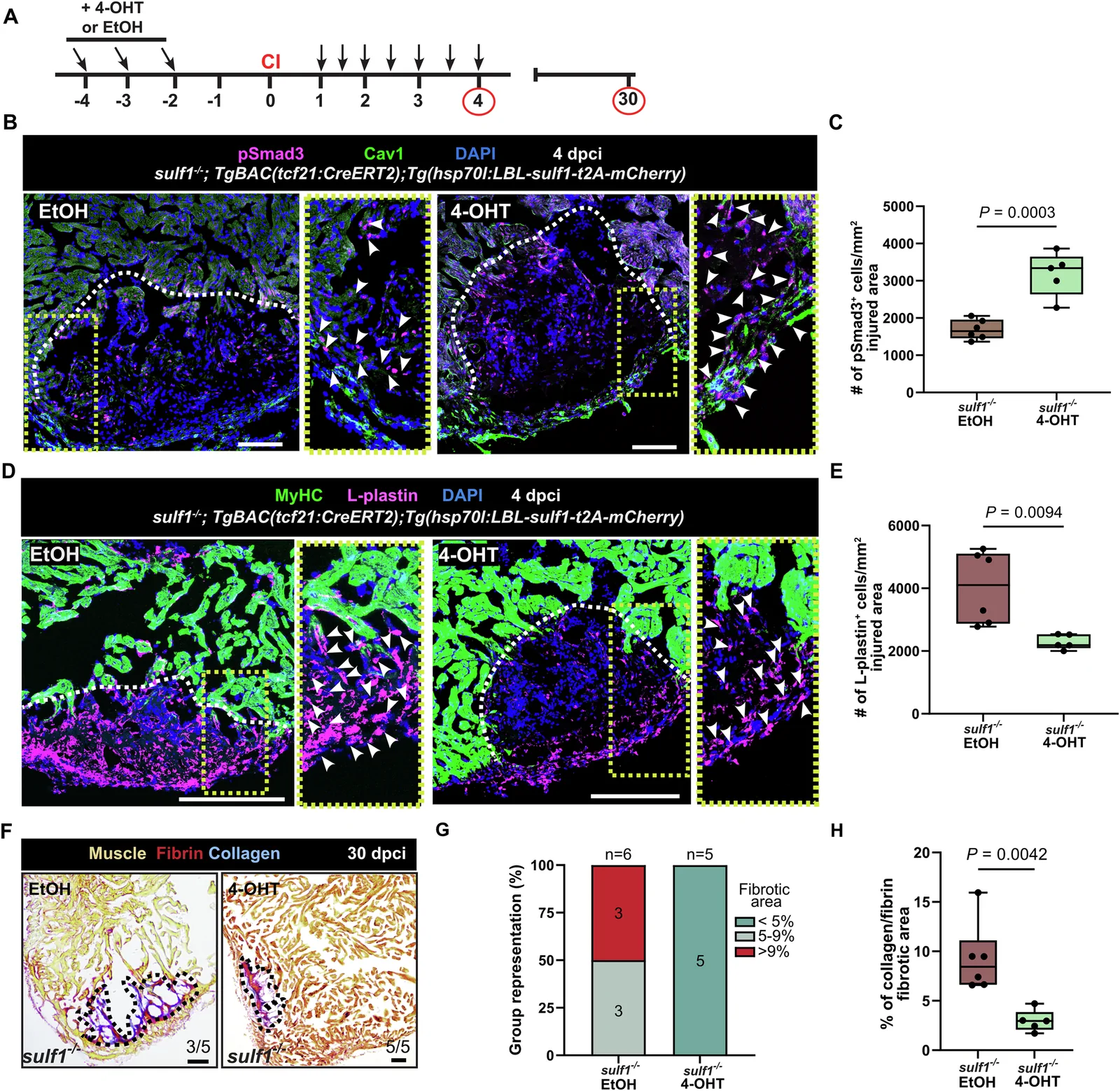
Epicardial overexpression of *sulf1* in *sulf1*^*−/−*^ rescues TGF-β signaling, attenuates inflammation, and improves fibrosis resolution. (**A**) Schematic representation of 4-hydroxytamoxifen (4-OHT) or ethanol (EtOH) treatments followed by cardiac cryoinjury (CI) and heat-shock treatments (arrows) in *sulf1*^*−/−*^ ventricles. (**B**) 4 dpci *sulf1*^*−/−*^ EtOH-treated and *sulf1*^*−/−*^ 4-OHT-treated ventricular sections stained for pSmad3 (magenta), Cav1 (EPCDs, green), and DAPI (DNA, blue). White arrowheads point to pSmad3^+^ cells. (**C**) Number of pSmad3^+^ cells per mm^2^ injured area in *sulf1*^*−/−*^ EtOH-treated (*n* = 6) and *sulf1*^*−/−*^ 4-OHT-treated (*n* = 5) ventricles at 4 dpci. (**D**) 4 dpci *sulf1*^*−/−*^ EtOH-treated and *sulf1*^*−/−*^ 4-OHT-treated ventricular sections stained for MyHC (CMs, green), L-plastin (leukocytes, magenta), and DAPI (DNA, blue). Arrowheads point to L-plastin^+^ cells. (**E**) Number of L-plastin^+^ cells per mm^2^ injured area in *sulf1*^*−/−*^ EtOH-treated (*n* = 6) and *sulf1*^*−/−*^ 4-OHT-treated (*n* = 5) ventricles at 4 dpci. (**F**) 30 dpci *sulf1*^*−/−*^ EtOH-treated and *sulf1*^*−/−*^ 4-OHT-treated ventricular sections stained with AFOG for muscle (yellow), collagen (blue), and fibrin (red). (**G**) Percentage representation of groups of different fibrotic area sizes at 30 dpci for cryoinjured *sulf1*^*−/−*^ EtOH-treated (*n* = 6) and *sulf1*^*−/−*^ 4-OHT-treated (*n* = 5) ventricles. Black dotted lines delineate the area with collagen and fibrin deposits. (**H**) Percentage of collagen/fibrin in fibrotic area in *sulf1*^*−/−*^ EtOH-treated (*n* = 6) and *sulf1*^*−/−*^ 4-OHT-treated (*n* = 5) ventricles at 30 dpci. Scale bars: 100 µm. White dashed lines delineate the injured area. Statistical tests: Student’s *t* test (**C**, **E**, **H**). For the boxplots, the center line indicates the median; the box bounds represent the 25th and 75th percentiles; the whiskers extend to the most extreme data points; and each point represents an independent measurement. Source data are available online for this figure.

Finally, we sought to determine whether *sulf1* overexpression in mutant EPDCs would rescue the mutant TGF-β signaling and inflammation phenotypes. We found that overexpression of *sulf1* in *sulf1*^*−/−*^ EPDCs leads to increased pSmad3⁺ cells within the injured at 4 dpci (Fig. 8A–C). Interestingly, the overexpression of *sulf1* in *sulf1*^*−/−*^ EPDCs leads to a reduced number of L-plastin⁺ cells at 4 dpci compared with EtOH-treated *sulf1*^*−/−*^ siblings (Fig. 8D,E). Lastly, epicardial overexpression of *sulf1* in *sulf1*^*−/−*^ significantly reduced fibrosis at 30 dpci compared with EtOH-treated *sulf1*^*−/−*^ siblings (Fig. 8F–H).

Overall, these data show that epicardial Sulf1 modulates TGF-β signaling, inflammation, and fibrosis resolution during heart regeneration.

## Discussion

Here, we show that the activated epicardium upregulates *sulf1* in response to cardiac injury and that this induction is required for TGF-β-mediated epicardial activation. Loss of *sulf1* attenuates TGF-β signaling and compromises CM regeneration, leading to increased late-stage fibrosis. We further identify that the inflammatory response is exacerbated in injured *sulf1*^*−/−*^ ventricles, as evidenced by alterations in specific macrophage subpopulations. Interestingly, our data revealed that macrophage recruitment and inflammation are regulated by TGF-β signaling. Collectively, these findings highlight the role of Sulf1 as a critical modulator of cardiac regeneration through its dual role in controlling TGF-β-dependent epicardial activation and shaping the inflammatory response.

A number of transcription factors and signaling pathways have been shown to regulate epicardial EMT (Cao and Poss, 2018; Quijada et al, 2020). Extracellular endo-sulfatases are known to fine-tune signaling pathways involved in development and disease by modulating the bioavailability of growth factors within the ECM (Korf-Klingebiel et al, 2019; Meyers et al, 2013; Okada et al, 2017; preprint: Redpath et al, 2025). This capability supports the idea that endo-sulfatases act as ECM modulators and may stimulate regenerative programs. Specifically, SULF1 has been shown to regulate TGF-β signaling (Fang et al, 2024; Justo et al, 2022), a pathway known to control epicardial EMT during cardiac regeneration(Xia et al, 2022). Our data show that *sulf1* is dynamically upregulated in the injury-induced activated epicardium and its abrogation attenuates epicardial TGF-β signaling. Consistently, we observed increased GAG sulfation in the mutant epicardium. Sulf1-dependent sulfation regulates TGF-β ligand release (Dhanasekaran et al, 2015). Therefore, these findings support the notion that the lack of Sulf1 leads to elevated HSPG sulfation levels, which may restrict TGF-β ligand availability and subsequently reduce signaling. We also observed a reduction in the expression of *aldh1a2* (Kikuchi et al, 2011; Lepilina et al, 2006) and *ptx3a* (Xia et al, 2022) in *sulf1* mutant EPDCs, indicating deficient epicardial activation. scRNA-seq analyses further revealed a loss of injury-induced epicardial populations in the mutant ventricles. Attenuation of TGF-β signaling in mutant EPDCs was confirmed by the downregulation of the TGF-β ligands (*tgfb1a, tgfb1b, tgfb2*, and *tgfb3*) and canonical downstream TGF-β target genes (*snai1a, snai1b, snai2, twist2*) (Xu et al, 2009). These findings highlight Sulf1 as a key ECM component modulating TGF-β-dependent epicardial activation, suggesting that targeting ECM-signaling interactions may enhance regenerative outcomes.

Previous studies have implicated TGF-β/Smad3 signaling in promoting CM regeneration by enabling an EMT-like response in EPDCs (Chablais and Jaźwińska, 2012; Peng et al, 2021). Consistent with this evidence, we observed that defective epicardial TGF-β signaling in *sulf1* mutant hearts reduces CM proliferation and dedifferentiation, both required for CM regeneration. In addition, disruptions in TGF-β signaling are known to impair the fibrotic response after injury (Frangogiannis, 2020; Massagué and Sheppard, 2023; Shi et al, 2020). We observed that *sulf1* mutants have increased late-stage fibrosis, being consistent with studies showing that altered TGF-β signaling can impair fibrosis resolution (Frangogiannis, 2020; Massagué and Sheppard, 2023; Shi et al, 2020). Similarly, Sulf1-deficient mice also develop larger scars after MI (Korf-Klingebiel et al, 2019). Overall, these findings show the importance of Sulf1 in regulating epicardial TGF-β signaling to facilitate the establishment of a regenerative niche and limit late-stage fibrosis.

Cardiac damage reanimates the epicardium early after injury to modulate tissue repair (Cao and Poss, 2018; Quijada et al, 2020; Ross Stewart et al, 2022). Coinciding with this early epicardial activation, an intense inflammatory cascade is initiated to remove necrotic tissue and establish a conducive environment for regeneration (Bevan et al, 2020; Iribarne, 2021; Peterson et al, 2024; Wei et al, 2023). In organisms with robust cardiac regenerative capacity, such as zebrafish, both epicardial activation and the inflammatory response are tightly regulated (Ross Stewart et al, 2022). In contrast, the adult mammalian heart is unable to efficiently regulate these responses, resulting in a failure to resolve injury and an inability to regenerate (Aurora et al, 2014; Godwin et al, 2017; Quijada et al, 2020; Zhou et al, 2011). A recent study showed that *ptx3a*^*+*^ epicardial progenitor cells associate with macrophages after cardiac injury (Sun et al, 2024). Moreover, PTX3 has been directly implicated in inflammation resolution, where it promotes macrophage polarization in idiopathic pulmonary fibrosis(Doni et al, 2021). Here, we show that epicardial EMT and activation of *ptx3a*^*+*^ epicardial progenitor cells are disrupted in *sulf1* mutants, which also present with altered inflammation. Together, our findings underscore the importance of activated epicardial cells in shaping an environment that supports cardiac regeneration.

Consistent with the role of epicardial-derived signals in influencing the immune response (Doni et al, 2021; Sun et al, 2024), our data show that Sulf1-mediated epicardial activation controls the inflammatory response after cardiac injury. We found that loss of *sulf1* leads to accumulation of macrophages in the injured area. Our transcriptomic analyses further reveal that the mutant macrophages exhibit a pro-inflammatory transcriptional signature indicative of altered macrophage polarization and activation. In line with previous studies that show that increased inflammation can alter CM regeneration after injury (Feng et al, 2025; S. Xu et al, 2019), we observed reduced CM proliferation and dedifferentiation in injured mutant ventricles.

Given that TGF-β signaling has been previously implicated in macrophage activation (FZhang et al, 2016), we hypothesized that altered TGF-β signaling in *sulf1*^*−/−*^ hearts might be the underlying cause for the increased inflammatory response. Indeed, pharmacological inhibition of TGF-β signaling in wild-type animals induced a similar phenotype, including the upregulation of inflammatory genes that recapitulated the *sulf1* mutant macrophage transcriptional profile. These findings support a model in which epicardial Sulf1 regulates macrophage responses after injury by modulating epicardial TGF-β signaling. Extending this link, we further show that a feedback mechanism operates between these two processes. Dampening inflammation in *sulf1* mutant hearts restored TGF-β signaling to wild-type levels and improved fibrosis resolution.

Finally, to further test the proposed model of Sulf1-mediated coordination of epicardial TGF-β activation and inflammation after injury, we used the HOTCre system to achieve spatiotemporal regulation of *sulf1* expression (Hesselson et al, 2009). Overexpression of *sulf1* in wild-type EPDCs enhanced TGF-β signaling and epicardial activation while limiting inflammation after injury. Induction of *sulf1* in mutant EPDCs reversed the TGF-β signaling and inflammation phenotypes. These findings underscore the role of epicardial Sulf1 in regulating the epicardial-immune crosstalk during regeneration.

Taken together, our data indicate that Sulf1 modulates epicardial TGF-β activation to regulate cardiac inflammation after injury. Epicardial and immune responses may influence the behavior of other tissues during regeneration, including the cardiac endothelium. Although we observed no obvious changes in endothelial cells under uninjured conditions (Appendix Fig. S4A), future studies are needed to elucidate the interactions between these cell types during the regenerative process. Overall, our results provide a strong foundation to further explore the Sulf1-TGF-β axis as a driver of both epicardial and immune responses after injury, potentially paving the way for future regenerative therapies.

## Methods

**Table:** Reagents and tools table

Reagent/resource	Reference or source	Identifier or catalog number
**Experimental models**	**Zebrafish (Danio rerio***,* **AB strain)**	
*TgBAC(tcf21:NTR-mCherry)* ^*pd108*^	PMID: 25938716	*pd108*
*TgBAC(cryaa:EGFP, tcf21:CreERT2)* ^*pd42*^	PMID: 21653610	*pd42*
*Tg(mpeg1.1:mCherryF)* ^*ump2*^	PMID: 24567393	*ump2*
*sulf1* ^*sjr9*^	This study	*sjr9*
*sulf1* ^*sjr10*^	This study	*sjr10*
*TgBAC(sulf1:EGFP)* ^*sjr13*^	This study	*sjr13*
*Tg(hsp70l:loxP-BFP-loxP-sulf1-t2A-mCherry)* ^*sjr15*^	This study	*sjr15*
**Recombinant DNA**		
BAC CH211-245B7	Bacpac genomics	
**Antibodies**		
Anti-Mef2	Boster Bio	DZ01398-1
Anti-embryonic myosin heavy chains	DSHB	N2.261
Anti-myosin heavy chains	DSHB	MF20
Anti-GFP	Aves Labs	GFP-1020
Anti-PCNA	Santa Cruz Biotechnology	sc-56
Anti-Caveolin 1	BD Transductions Laboratories	610407
Anti-pSmad3	Abcam	ab52903
Anti-DsRed	Takara Bio	632496
Anti- L-plastin	Genecust	
Anti- Aldh1a2	Genetex	GTX124302
Anti-aSMA	Genetex	GTX100034
Alexa Fluor 488 Goat Anti-chicken IgG (H + L)	Thermo Fisher Scientific	A-11039
Alexa Fluor 488 Goat Anti-mouse IgG (H + L)	Thermo Fisher Scientific	A-11029
Alexa Fluor 488 Goat Anti-rabbit IgG (H + L)	Thermo Fisher Scientific	A-11008
Alexa Fluor 568 Goat Anti-mouse IgG (H + L)	Thermo Fisher Scientific	A-11004
Alexa Fluor 568 Goat Anti-rabbit IgG (H + L)	Thermo Fisher Scientific	A-11036
Alexa Fluor 647 Goat Anti-rabbit IgG (H + L)	Thermo Fisher Scientific	A-21244
Alexa Fluor 647 Goat Anti-chicken IgG (H + L)	Thermo Fisher Scientific	A-21449
**Oligonucleotides and other sequence-based reagents**		
PCR primers	This study	Appendix Table S1
exon 2 gRNA 1	Integrated DNA Technologies	5’-GATGGTCCTGGCCGCCCCACTGG-3
exon 2 gRNA 2	Integrated DNA Technologies	5’-TGCTTTGGTTTCACCACCCGCGG-3’
exon 19 gRNA	Integrated DNA Technologies	5’-CCCGTATCGCCCCAACCAGAGCC-3’
exon 3 gRNA	Integrated DNA Technologies	5’-GATCGTGAAGGACAGCACATGGG-3’
**Chemicals, enzymes, and other reagents**		
Tricaine	Sigma	E10521
Cas9 protein (Alt-R^TM^ S.p. Cas9 nuclease V3)	Integrated DNA Technologies	1081058
In-Fusion Snap Assembly Master Mix	Takara	638948
4-hydroxytamoxifen	Sigma	H7904
Citrate-based antigen unmasking solution	MJS BioLynx	VECTH3300
TRIzol	Invitrogen	15596026
RevertAid H Minus First Strand cDNA kit	Thermo Scientific	K1632
iTaq Universal SYBR Green Supermix	Bio-Rad	1725122
Calcium-, magnesium-free HBSS	Sigma	H9394
Heparin	Sigma	H3393
Collagenase	Gibco	17101015
1x TrypLE Express Enzyme	Gibco	12605010
Trypan Blue	Sigma-Aldrich	T8154
Dexamethasone	Sigma-Aldrich	D1756-25MG
TGF-β inhibitor	BOC Sciences	SB431542
Chromium Next GEM Single Cell 3’ Reagent Kit v3.1	10x Genomics	PN- 1000269
Dual Index Kit TT Set A	10x Genomics	PN- 1000215
**Software**		
Seurat v4		
Cell Ranger Count v6.1.2		
GraphPad Prism v.10		
Fiji (ImageJ)		
Adobe Illustrator		
affinity by Canva		
**Other**		
Leica TCS SP8 laser scanning confocal microscope	Leica	
Nikon SMZ18 stereomicroscope	Nikon	
CFX Opus 96 Real-Time PCR System	Bio-Rad	

### Zebrafish lines and manipulations

All experimental procedures involving zebrafish (*Danio rerio*) were conducted in adherence to the Canadian Council on Animal Care and national animal welfare regulations. Protocols involving animals were approved by the *Comité Institutionnel des Bonnes Pratiques Animales en Recherche* (CIPBAR; protocol no. 2021-3308 #833 and 2025-8341). Both male and female zebrafish aged between 3 and 12 months were used in this study. Zebrafish were raised in system water with 20 fish per 3-L tank. These conditions were maintained prior to and after experimental procedures. WT and mutants were raised in the same tanks and conditions and genotyped prior to experiments. Experiments were conducted without blinding. We used the previously published lines *TgBAC(tcf21:NTR-mCherry)*^*pd108*^ (hereafter *TgBAC(tcf21:mCherry)), TgBAC(cryaa:EGFP, tcf21:CreERT2)*^*pd42*^ *(*hereafter *TgBAC(tcf21:CreERT2))* and *Tg(mpeg1.1:mCherryF)*^*ump2*^. Adult zebrafish were anesthetized in 0.016% tricaine (Sigma, Cat. no. E10521) and cryoinjuries were performed as previously described (Chablais et al, 2011; González-Rosa et al, 2011).

### Generation of zebrafish mutant and transgenic lines

The CRISPR/Cas9 technology was used to generate the *sulf1*^*sjr9*^ (full locus deletion allele) and *sulf1*^*sjr10*^ (targeting the sulfatase domain, exon 3) mutants. Guide RNAs (gRNAs) targeting *sulf1* (GRCz11, NC_007135.7) were synthesized using the cloning-free method previously described (Varshney et al, 2016). To generate the *sulf1*^*sjr9*^ mutant allele, the following oligos were used to synthesize gRNAs targeting the exon 2 and exon 19: 5’-GATGGTCCTGGCCGCCCCACTGG-3’, 5’-TGCTTTGGTTTCACCACCCGCGG-3’ and 5’-CCCGTATCGCCCCAACCAGAGCC-3’ for the full locus deletion mutant. To generate the *sulf1*^*sj10*^ mutant allele, the following oligo was used to synthesize gRNAs targeting the exon 3: 5’- GATCGTGAAGGACAGCACATGGG-3’. One-cell stage embryos were injected with 100–120 pg of gRNAs and 2.5 μM of Cas9 protein (Alt-R^TM^ S.p. Cas9 nuclease V3, Integrated DNA Technologies, Cat. no. 1081058). Fish were genotyped by High-Resolution Melting (HRM) analysis/PCR and Sanger sequencing using primer sequences that can be found in Appendix Table S1 (Appendix Fig. S2; Fig. EV1). Adult *sulf1*^*sjr9/sjr9*^ *and sulf1*^*sjr10/sjr10*^ display no gross morphological defects, survive with expected Mendelian ratios, and are fertile. No alterations in epicardial morphology and ventricular surface area were observed between adult *sulf1*^*sjr9/sjr9*^ (hereafter *sulf1*^*−/−*^) and wild-type siblings (Appendix Fig. S4). The *TgBAC(sulf1:EGFP)*^*sjr13*^ line was generated by modification of a bacterial artificial chromosome (BAC) clone: BAC CH211-245B7 (Bacpac genomics) as previously described (Bussmann and Schulte-Merker, 2011; Suster et al, 2011). To create the *Tg(hsp70l:loxP-BFP-loxP-sulf1-t2A-mCherry)*^*sjr15*^ line (hereafter *Tg(hsp70l:LBL-sulf1-t2A-mCherry)* or *sulf1OE*), the *sulf1* coding sequence was amplified by PCR and cloned under the *hsp70l* promoter into a Tol2 plasmid using In-Fusion Snap Assembly Master Mix (Takara, Cat. no. 638948). 20 pg of *hsp70l:LBL-sulf1-t2A-mCherry* plasmid along with 30 pg of *Tol2* mRNA was injected into zebrafish one-cell stage embryos. Injected embryos were heat-shocked, and embryos positive for BFP were raised to adulthood and later screened for founder identification by screening the offspring for BFP expression following three heat-shock treatments at 39 °C for 1 h each. mCherry expression after heat-shock induction was prevented by the presence of the *sulf1* stop codon, confirmed by sequencing. This stop codon did not interfere with *sulf1* expression induction (Appendix Fig. S3A–D). For the HOTCre system (Hesselson et al, 2009), *Tg(hsp70l:LBL-sulf1-t2A-mCherry)*; *TgBAC(tcf21:CreERT2)* fish were recombined in system water supplemented with 5 µM 4-hydroxytamoxifen (Sigma, Cat. no. H7904) or vehicle (ethanol) for 12 h per day in the dark, for 3 consecutive days prior to cryoinjury. For heat-shock treatments following cryoinjury, all fish were incubated in preheated system water at 39 °C for an hour every 12 h until 4 dpci (Figs. 7A and 8A).

### Histological analyses, imaging, and quantification

For histological analyses, zebrafish hearts were fixed as described previously (El-Sammak et al, 2022; Kayman Kürekçi et al, 2026). Immunostaining was performed as previously described, with an initial additional step of antigen retrieval using citrate-based antigen unmasking solution (MJS BioLynx, Cat. no. VECTH3300) at 96 °C for 7 min(Marín-Juez et al, 2016). Primary antibodies used were anti-Mef2 (Boster Bio, Cat. no. DZ01398-1, 1:200), anti-embryonic myosin heavy chains (DSHB, Cat. no. N2.261, 1:50), anti-myosin heavy chains (DSHB, Cat. no. MF20,1:20), anti-GFP (Aves Labs, Cat. no. GFP-1020, 1:200), anti-PCNA (Santa Cruz Biotechnology, Cat. no. sc-56, 1:500, anti-Caveolin 1 (BD Transductions Laboratories, Cat. no. 610407, 1:100), anti-pSmad3 (Abcam, Cat. no. ab52903, 1:100), anti-DsRed (Takara Bio, Cat. no. 632496, 1:200), anti-L-plastin antibody (Genecust, 1:100), anti- Aldh1a2 antibody (Cat. no. GTX124302, 1:100) and anti- aSMA antibody (Cat. no. GTX100034, 1:200). Secondary antibodies used were Alexa Fluor 488 Goat Anti-chicken IgG (H + L) (Thermo Fisher Scientific, Cat. no. A-11039, 1:500), Alexa Fluor 488 Goat Anti-mouse IgG (H + L) (Thermo Fisher Scientific, Cat. no. A-11029, 1:500), Alexa Fluor 488 Goat Anti-rabbit IgG (H + L) (Thermo Fisher Scientific, Cat. no. A-11008, 1:500), Alexa Fluor 568 Goat Anti-mouse IgG (H + L) (Thermo Fisher Scientific, Cat. no. A-11004, 1:500), Alexa Fluor 568 Goat Anti-rabbit IgG (H + L) (Thermo Fisher Scientific, Cat. no. A-11036, 1:500), Alexa Fluor 647 Goat Anti-rabbit IgG (H + L) (Thermo Fisher Scientific, Cat. no. A-21244, 1:500), Alexa Fluor 647 Goat Anti-chicken IgG (H + L) (Thermo Fisher Scientific, Cat. no. A-21449, 1:500). DAPI (4′,6-diamidino-2-phenylindole) was used as a nuclear counterstain.

Imaging and quantification were performed using two or three non-superficial and non-consecutive sections for each biological replicate, as previously described (El-Sammak et al, 2022). Imaging was performed on a Leica TCS SP8 laser scanning confocal microscope. Quantification for *tcf21:*mCherry^+^, L-plastin^+^, αSMA^+^, and *mpeg1.1:*mCherryF^+^ cell numbers was performed in the injured tissue and in peripheral border zone areas (100 μm). For the quantification of pSmad3^+^ cells, cells co-localizing with Cav1 and located within the injury were counted. Quantification of CM proliferation and dedifferentiation was performed by counting N2.261^+^ and PCNA^+^ CMs within the 100 μm area around the injury border zone.

For Acid Fuchsin Orange G (AFOG) staining, 8-μm heart cryosections were hydrated in distilled water for 5 min and were incubated in Bouin’s solution for 2 h at 60 °C, followed by an hour at RT. Slides were rinsed in running tap water for 30 min until the water ran clear. Slides were then incubated in 1% phosphomolybdic acid for 90 s followed by rinsing in distilled water for 5 min and incubated in AFOG staining solution (0.5% Analine Blue, 1% Orange G, 1% Acid Fuchsin, pH 1.09) for 3 min. After rinsing in distilled water twice, slides were dehydrated twice in 95% EtOH, followed by two washes in absolute EtOH for 5 min each. Slides were then washed twice in xylene for 2 min each and mounted in Entellan (Sigma, Cat. no. 1079610500). Sections from AFOG staining were imaged using a Nikon SMZ18 stereomicroscope. For fibrotic area analyses, the fibrotic area (collagen + fibrin area) was calculated as a ratio from the total ventricular surface in at least three non-consecutive sections with the biggest injuries using ImageJ. Percentages of fibrotic areas (relative to ventricular areas) were then grouped based on their size.

HCR RNA-FISH (Molecular Instruments) was performed following the manufacturer’s protocol for fixed fresh frozen tissue sections by omitting the proteinase K treatment. For immunostainings following HCR, samples were processed as previously described, beginning from the blocking step (Marín-Juez et al, 2016). Imaging of RNA-FISH was performed on a Leica TCS SP8 laser scanning confocal microscope.

pH 1.0 Alcian blue staining was performed on tissue sections as previously described (Arunachalam and Sreeja, 2025; Cervino et al, 2026). Images were acquired with a Nikon SMZ18 stereomicroscope.

### Quantitative real-time PCR

Total RNA was isolated from pools of five mechanically homogenized larvae at 5 dpf or from pools of two ventricles per biological replicate using TRIzol (Invitrogen, Cat. no. 15596026) following the manufacturer’s instructions. cDNA synthesis was performed following the manufacturer’s instructions (RevertAid H Minus First Strand cDNA kit, Thermo Scientific, Cat. no. K1632). RT-qPCR reactions were performed using iTaq Universal SYBR Green Supermix (Bio-Rad, Cat. no. 1725122) on the CFX Opus 96 Real-Time PCR System (Bio-Rad). Expression levels of genes of interest were normalized to the expression of *rpl13a*, and expression fold changes were calculated using the ΔΔCt method (Livak and Schmittgen, 2001). The primer sequences used are provided in Appendix Table S1.

### Single-cell dissociation

As previously shown (Kayman Kürekçi et al, 2026), pools of four uninjured ventricles and 4 dpci cryoinjured ventricles were extracted from *sulf1*^*−/−*^ zebrafish. Fish were anesthetized, and ventricles were extracted in calcium-, magnesium-free HBSS (Sigma, Cat. no. H9394) supplemented with 20 U/ml of heparin (Sigma, Cat. no. H3393). Ventricles were washed in fresh HBSS and digested into 1 ml of 1 mg/ml of collagenase (Gibco, Cat. no. 17101015) in HBSS for 30 min on a thermal mixer at 32 °C and 700 rpm. Ventricles were mechanically disrupted by pipetting every 10 min. Samples were centrifuged at 2000 rpm for 5 min at RT, and supernatants were discarded. Cells were digested with 1 ml of 1× TrypLE Express Enzyme (Gibco, Cat. no. 12605010) for 15 min on a thermal mixer at 32 °C, 700 rpm and pipetted every 5 min. Enzymatic digestion was stopped by adding 1 ml of 20% fetal bovine serum in HBSS to the extracts. Tissue extracts were strained through a 70 µm cell strainer and centrifuged at 2000 rpm for 5 min at RT. The supernatants were discarded, and pellets were washed with 1 ml of 20% fetal bovine serum in HBSS. After centrifugation at 2000 rpm for 5 min at RT, cells were resuspended in 80 µl of 20% fetal bovine serum in HBSS. Cells were counted, and viability (>85%) was assessed using 0.4% Trypan Blue (Sigma-Aldrich, Cat. no. T8154). Cells were kept on ice and immediately processed for scRNA-seq.

### Single-cell RNA sequencing and analysis

For the scRNA-seq experiment, as previously shown (Kayman Kürekçi et al, 2026), 14,000 cells were used for each sample, and the Chromium Next GEM Single Cell 3’ Reagent Kit v3.1 (10x Genomics, Cat. no. PN- 1000269) was used following the manufacturer’s protocol. Each sample was indexed individually using the Dual Index Kit TT Set A (10x Genomics, Cat. no. PN- 1000215). After quality control, libraries were run on a NovaSeq 6000 system (Illumina) at a depth of ~300 M reads/sample. FASTQ files were processed individually in 10x Genomics Cloud Analysis using the Cell Ranger Count v6.1.2 pipeline, and reads were aligned to GRCz11 v4.3.2 reference genome (Lawson et al, 2020). Filtered gene expression matrices were imported and further processed in Seurat v4 (Hao et al, 2021). Samples were integrated to create a single integrated Seurat object, and low-quality cells containing fewer than 200 unique features were removed. Batch effect correction was performed using the data integration and ScTransform functions in Seurat. To increase the retrieval of CMs characterized by high mitochondrial RNA content, an initial filtering was applied to remove cells with more than 40% mitochondrial RNA. After initial clustering and annotation of CMs, non-CM cells with greater than 12% mitochondrial RNA were excluded from further analysis. The remaining cells were then re-clustered and annotated based on the expression of marker genes. Following normalization, data visualization was done in Seurat, incorporating previously published R code with modifications (Carey et al, 2024).

### Drug treatment

In total, 10 μl Dexamethasone (Cat. no. D1756-25MG, Sigma-Aldrich) at a concentration of 0.5 mg/ml in sterile 0.68% saline or PBS (Ma et al, 2021) was injected intraperitoneally at 1 day before cryoinjury, then once daily until 4 dpci. For the TGF-β inhibitor experiment, fish were incubated in system water supplemented with TGF-β inhibitor (Cat. no. SB431542, 50 mg BOC Sciences) or DMSO at a concentration of 10 µM per fish starting 1 day prior to cryoinjuries until 4 dpci when the hearts were extracted. The inhibitor was refreshed every day.

### Epicardial explant culture

Epicardial explants were cultured as previously described (Kim et al, 2012) with a few modifications. The culture medium was refreshed on day 3, and the explants were cultured for 5 days. Immunostaining was performed as previously described (Cao et al, 2017). Imaging was performed on a Leica TCS SP8 laser scanning confocal microscope. Outgrowth distance was quantified as previously described (Kim et al, 2012).

### Statistical analysis

Statistical analyses and graphics were performed using GraphPad Prism v.10. For each dataset, normality was assessed using the Shapiro–Wilks normality test (alpha value of 0.05). When two groups were compared, comparative statistics were performed using Student’s *t* test for a parametric test or Mann–Whitney *U* test for a nonparametric test, as stated in the figure legends. One-way ANOVA followed by Tukey’s post hoc tests was used when more than two groups were compared, as stated in the figure legends. Error bars represent mean ± SD, and bars represent median values. The exact *P* values are indicated in the figures. Significance level was set at <0.05 for all tests.

## Supplementary information

Appendix

Peer Review File

Source data Fig. 1

Source data Fig. 2

Source data Fig. 3

Source data Fig. 5

Source data Fig. 6

Source data Fig. 8

Source data Fig. 7

Expanded View Figures

## Data Availability

Raw sequencing files are available in the following databases: RNA-Seq data: Gene Expression Omnibus GSM8898315 (wild-type uninjured dataset). RNA-Seq data: Gene Expression Omnibus GSM8898316 (wild-type 4 dpci dataset). RNA-Seq data: Gene Expression Omnibus GSM9255046 (*sulf1*^*−/−*^ uninjured dataset). RNA-Seq data: Gene Expression Omnibus GSM9255047 (*sulf1*^*−/−*^ 4 dpci dataset). The source data of this paper are collected in the following database record: biostudies:S-SCDT-10_1038-S44319-026-00910-5.
