# Supplementary material for: Sulf1 regulates inflammation via modulation of TGF-β dependent epicardial activation
Source: EMBO Rep. 2026 Aug 27;27(19):5694–719. doi: 10.1038/s44319-026-00910-5 (PMC13649710; doi:10.1038/s44319-026-00910-5)
Supplement: Supplementary file 1 [file 44319_2026_910_MOESM10_ESM.pdf]

Expanded View Figures

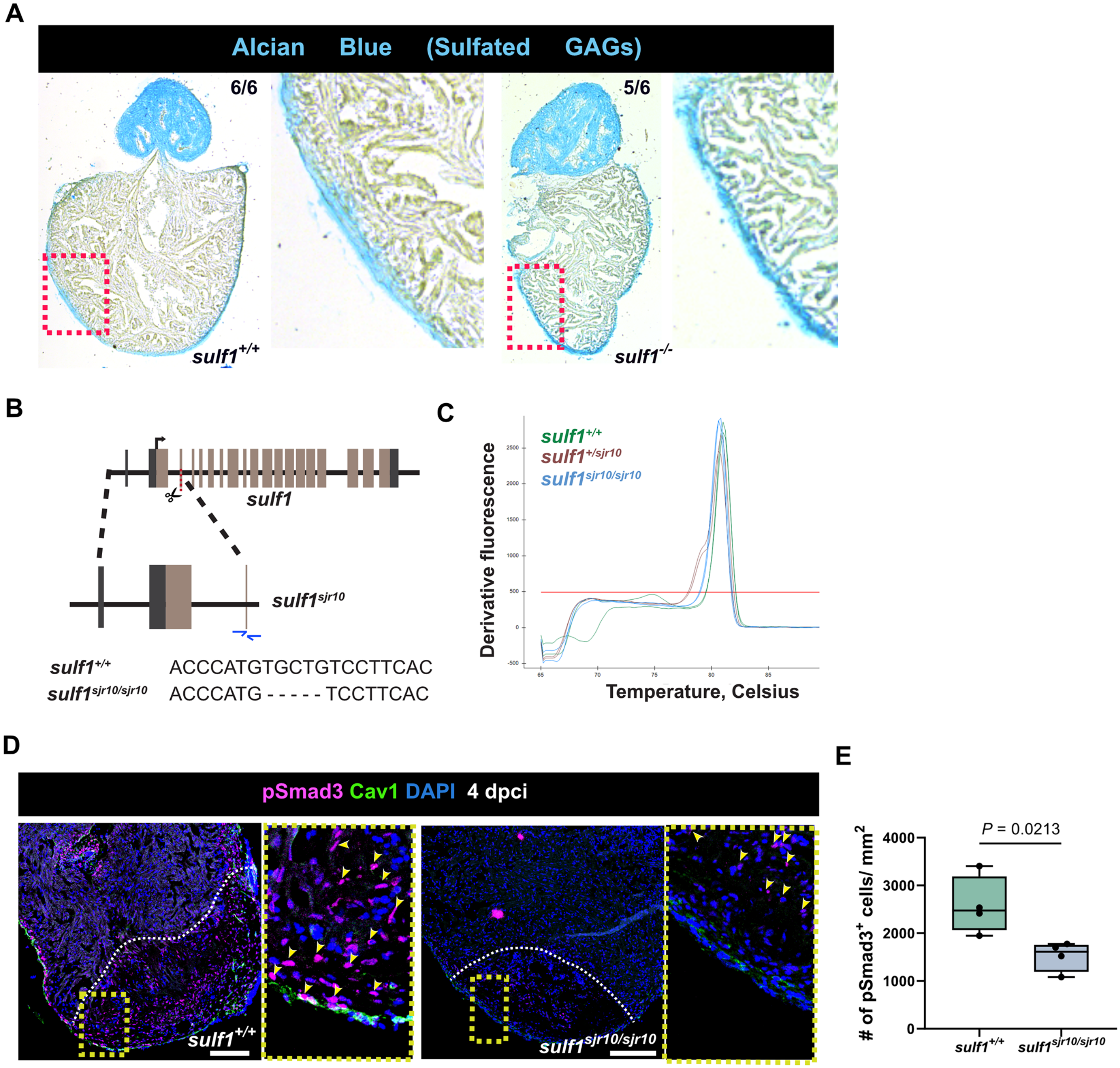

Figure EV1. Sulfated glycosaminoglycan (GAG) assessment and pSmad3 levels in *sulf1*^{sjr10}.

(**A**) *sulf1*$^{+/+}$ ($n = 6$) and *sulf1*$^{-/-}$ ($n = 6$) uninjured ventricular sections stained for Alcian Blue (sulfated GAGs, blue). (**B**) Schematic representation of *sulf1* locus showing gRNA (pink line) targeting exon 3 to generate *sulf1*^{sjr10} allele. Exons are shown as brown boxes and UTRs as black boxes. Genotyping primer positions are represented with blue arrows. *sulf1*$^{sjr10/sjr10}$ has a 5-nucleotide deletion in the region encoding the sulfatase domain, leading to a premature stop codon. (**C**) High-resolution melt analysis used for genotyping. Melting curves for 2 independent fish of each genotype are shown. (**D**) 4 dpci *sulf1*$^{+/+}$ and *sulf1*$^{sjr10/sjr10}$ ventricular sections stained for pSmad3 (magenta), Cav1 (EPCDs, green), and DAPI (DNA, blue). Yellow arrowheads point to pSmad3^{+} cells. White dashed lines delineate the injured area. Scale bars: 100 µm. (**E**) Number of pSmad3^{+} cells per mm^2 injured area in *sulf1*$^{+/+}$ ($n = 4$) and *sulf1*$^{sjr10/sjr10}$ ($n = 4$) ventricles at 4 dpci. Statistical tests: Student's *t* test. For the boxplot, the center line indicates the median; the box bounds represent the 25th and 75th percentiles; the whiskers extend to the most extreme data points; and each point represents an independent measurement.

 © The Author(s)

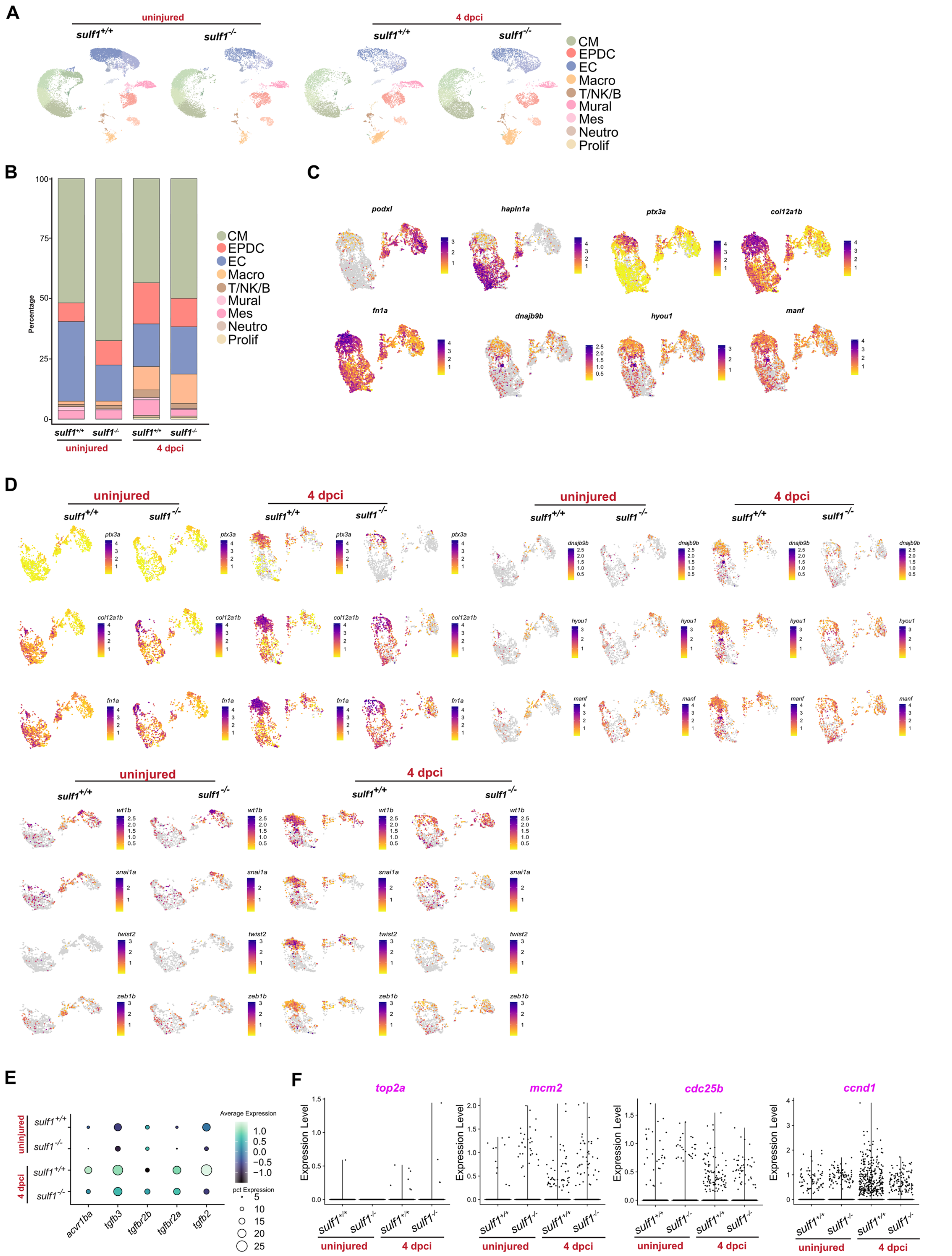
A
uninjured
4 dpci
sulf1+/+
sulf1-/-
CM
EPDC
EC
Macro
T/NK/B
Mural
Mes
Neutro
Prolif
B
Percentage
uninjured
4 dpci
C
podxl
hapln1a
ptx3a
col12a1b
fn1a
dnajb9b
hyou1
manf
D
wt1b
snai1a
twist2
zeb1b
E
Average Expression
pct Expression
acvr1ba
tgfb3
tgfbr2b
tgfbr2a
tgfb2
F
top2a
mcm2
cdc25b
ccnd1
Expression Level

◀ **Figure EV2. Transcriptional changes in EPDCs subclusters of *sulf1*$^{-/-}$ mutants.**

(**A**) UMAP representation of individual groups depicting cluster changes between all groups. (**B**) Proportion of cells per cluster within each sample. (**C**) UMAPs showing normalized expression of marker genes of epithelial EPDCs (*podxl*), mesenchymal EPDCs (*hapln1a*), genes enriched in EPI 2 cluster (*ptx3a, col12a1b, fn1a),* and genes enriched in EPI 8 cluster (*dnajb9b, hyou1, manf*) in all samples combined. (**D**) UMAPs showing normalized expression of *ptx3a, col12a1b, fn1a, dnajb9b, hyou1, manf*, *wt1b, snai1a, twist2* and *zeb1b* in individual groups. (**E**) Dot plot depicting average expression and abundance (pct. exp) of *acvr1ba, tgfb3, tgfbr2b, tgfbr2a*, and *tgfb2* in EPDCs of individual groups. (**F**) Violin plots showing expression of *top2a*, *mcm2, cdc25b*, and *ccnd1* in EPDCs of individual groups.

 © The Author(s)

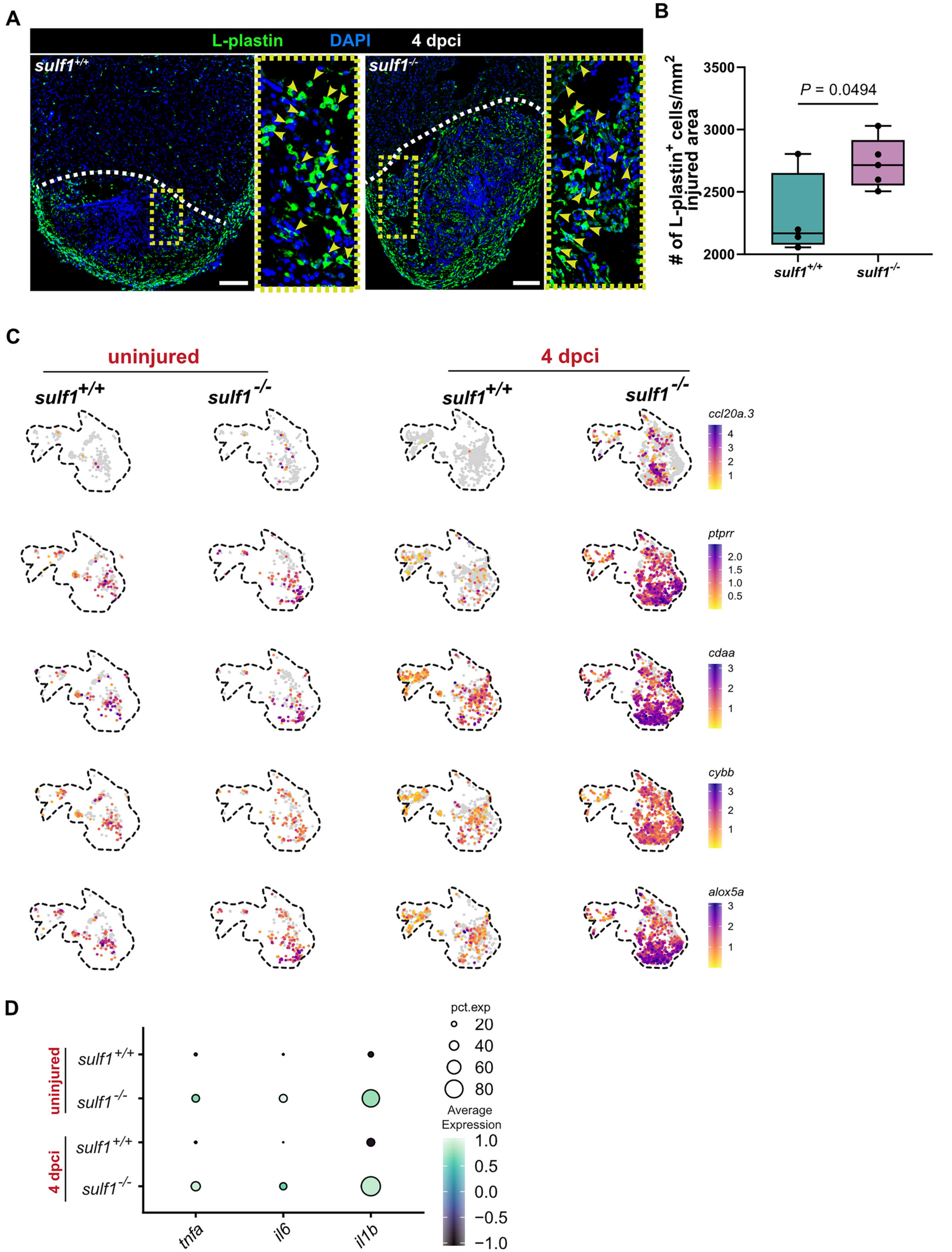
A
L-plastin DAPI 4 dpci
sulf1+/+
sulf1-/-
B
P = 0.0494
# of L-plastin+ cells/mm2 injured area
3500
3000
2500
2000
sulf1+/+
sulf1-/-
C
uninjured
4 dpci
sulf1+/+
sulf1-/-
sulf1+/+
sulf1-/-
ccl20a.3
ptprr
cdaa
cybb
alox5a
D
uninjured
4 dpci
sulf1+/+
sulf1-/-
sulf1+/+
sulf1-/-
tnfa
il6
il1b
pct.exp
20
40
60
80
Average Expression
1.0
0.5
0.0
−0.5
−1.0

Figure EV3. Transcriptional changes in macrophage subclusters of *sulf1*$^{-/-}$ mutants.

(**A**) 4 dpci *sulf1*$^{+/+}$ and *sulf1*$^{-/-}$ ventricular sections stained for L-plastin (leukocytes, green) and DAPI (DNA, blue). Arrowheads point to L-plastin^{+} cells. White dashed lines delineate the injured area. Scale bars: 100 µm. (**B**) Number of L-plastin^{+} cells per mm^2 injured area in *sulf1*$^{+/+}$ ($n = 4$) and *sulf1*$^{-/-}$ ($n = 5$) ventricles at 4 dpci. Statistical test: Student's *t* test. (**C**) UMAPs showing the normalized expression of *ccl20a.3, ptprr, cdaa, cybb*, and *alox5a* in macrophages of individual groups. (**D**) Dot plot depicting average expression and abundance (pct. exp) of *tnfa, il6*, and *il1b* in macrophages of individual groups.

 © The Author(s)
