# Supplementary material for: Sulf1 regulates inflammation via modulation of TGF-β dependent epicardial activation
Source: EMBO Rep. 2026 Aug 27;27(19):5694–719. doi: 10.1038/s44319-026-00910-5 (PMC13649710; doi:10.1038/s44319-026-00910-5)
Supplement: Supplementary file 2 [file 44319_2026_910_MOESM1_ESM.pdf]

Sulf1 regulates inflammation via modulation of TGF-β dependent epicardial activation

Gursimran Kaur Bajwa[1,2], Gülsüm Kayman-Kürekçi[1,2], Rubén Marín-Juez[1,2]*

Table of Contents Appendix Figures and Tables:

Appendix Figures:

Appendix Figure S1……………………………………………………………………2

Appendix Figure S2……………………………………………………………………3

Appendix Figure S3……………………………………………………………………4

Appendix Figure S4……………………………………………………………………5

Appendix Tables:

Appendix Table S1……………………………………………………………………6

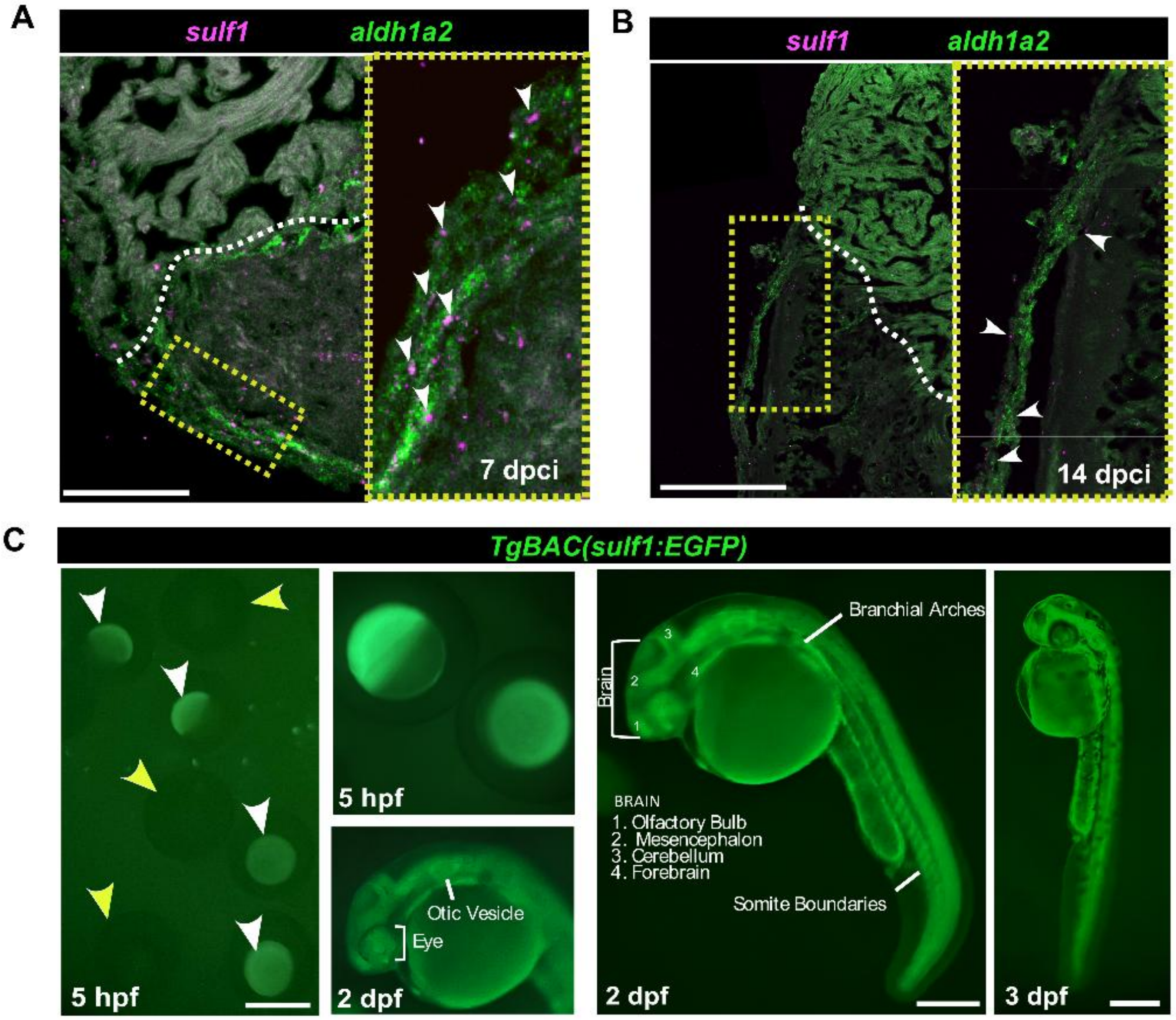

Appendix Figure S1: *sulf1* expression after cryoinjury and during early developmental stages.

A. HCR for *sulf1* (magenta) and *aldh1a2* (green) on wild-type ventricular sections at 7 dpci. Inset shows a high-magnification image of EPDCs expressing *sulf1*. Arrowheads point to *sulf1*$^+$ cells. White dotted lines delineate the injured area. Scale bar: 100 µm. **B.** HCR for *sulf1* (magenta) and *aldh1a2* (green) on wild-type ventricular sections at 14 dpci. Inset shows a high-magnification image of EPDCs expressing *sulf1*. Arrowheads point to *sulf1*$^+$ cells. White dotted lines delineate the injured area. Scale bar: 100 µm. **C.** Wholemount images of *TgBAC(sulf1:EGFP)* at 5 hpf, 2 and 3 dpf. White and yellow arrowheads point to *sulf1:*EGFP$^+$ and *sulf1:*EGFP$^-$ embryos, respectively. Scale bar: 100 µm.

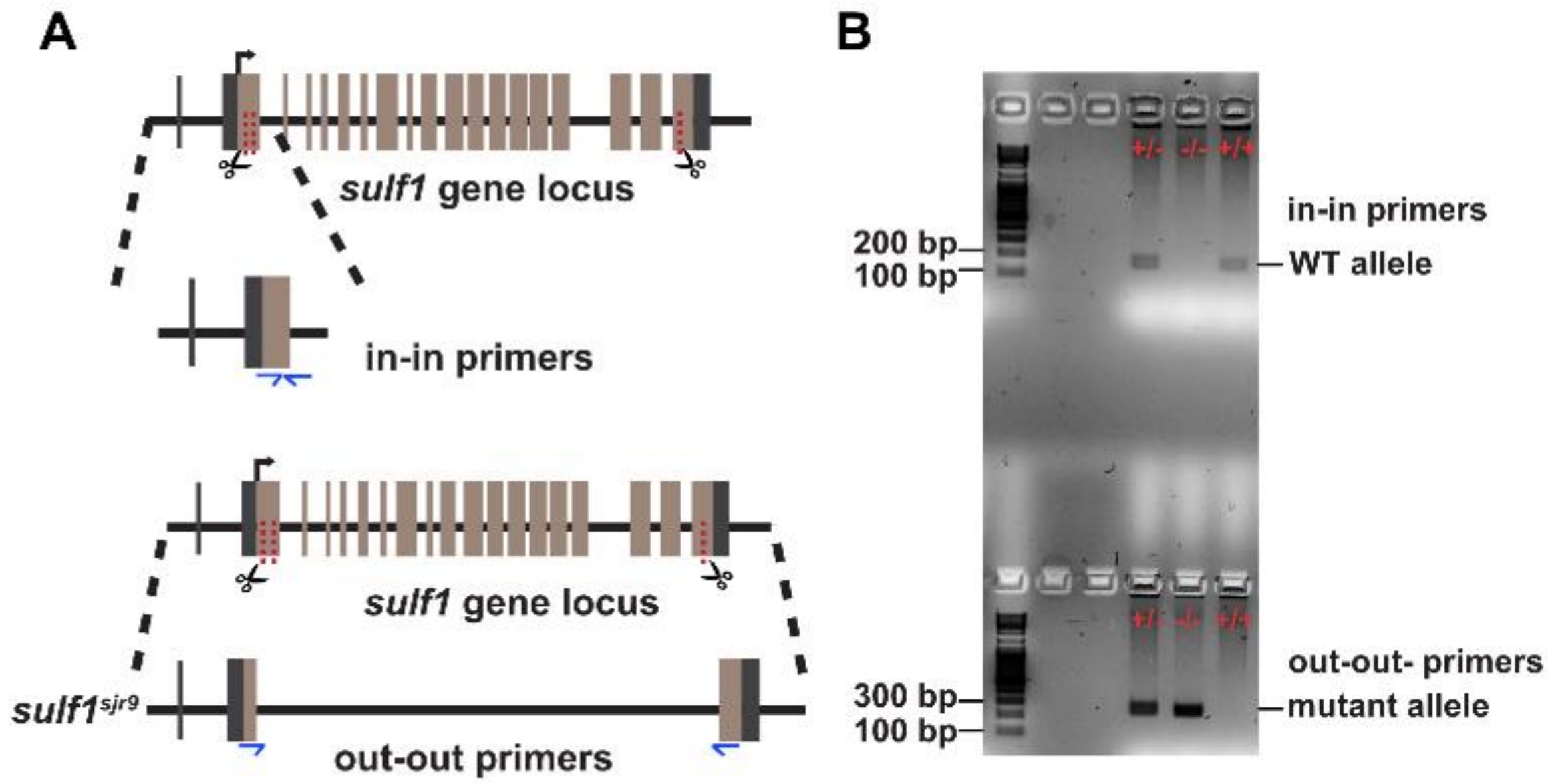

Appendix Figure S2: *sulf1* full locus deletion allele generation strategy.

A. Schematic representation of *sulf1* locus showing gRNAs (pink lines) targeting exon 2 and exon 19 to generate a full-locus deletion allele (*sulf1*^{sjr9}). Exons are shown as brown boxes and UTRs as black boxes. Genotyping primer position is represented in blue. **B.** DNA gel electrophoresis analysis of PCR products amplified from DNA extracted from fin-clips. Inner primers are located at the junction of the deleted region and amplify the wild-type allele; outer primers span the genomic deletion and amplify the mutant allele.

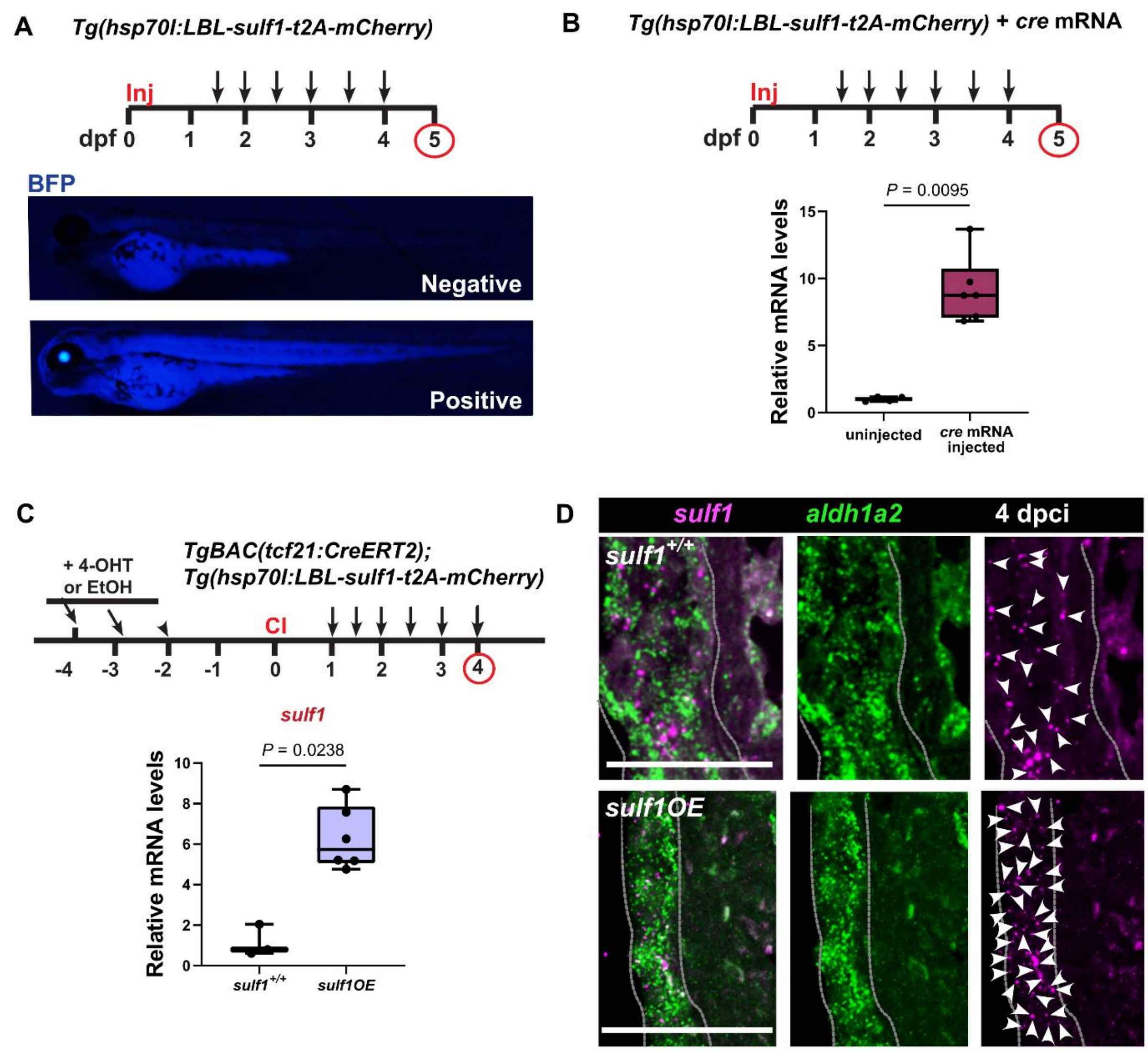

Appendix Figure S3: Generation of the *sulf1* overexpression line.

A. Schematic representation of the heat-shock treatments applied to *Tg(hsp70l:LBL-sulf1-t2A-mCherry*) fish. 5 dpf larvae showing global BFP expression after the heat-shock treatment. **B.** Schematic representation of the heat-shock regimen applied *Tg(hsp70l:LBL-sulf1-t2A-mCherry*) fish injected with *cre* mRNA at the one-cell stage. RT-qPCR analysis of *sulf1* mRNA levels in 5 dpf *Tg(hsp70l:LBL-sulf1-t2A-mCherry*) uninjected (n=6) and *cre* mRNA injected (n=6) larvae subjected to heat-shock. **C.** Schematic representation of 4-hydroxytamoxifen (4-OHT) or ethanol (EtOH) treatments followed by cardiac cryoinjury (CI) and heat-shock treatment (arrows) in *sulf1*$^{+/+}$ and *TgBAC(tcf21:CreERT2)*;*Tg(hsp70l:LBL-sulf1-t2A-mCherry*) (*sulf1OE*) ventricles. RT-qPCR analysis of *sulf1* mRNA levels in *sulf1*$^{+/+}$ (n=3) and *sulf1OE* (n=6) ventricles subjected to heat-shock **D.** HCR for *sulf1* (magenta) and *aldh1a2* (EPDCs, green) on *sulf1*$^{+/+}$ and *sulf1OE*

ventricular sections at 4 dpci. Arrowheads point to *sulf1*$^{+}$ cells. Scale bars: 100 µm. Statistical test: Mann-Whitney test. For the boxplots, the center line indicates the median; the box bounds represent the 25th and 75th percentiles; the whiskers extend to the most extreme data points; and each point represents an independent measurement.

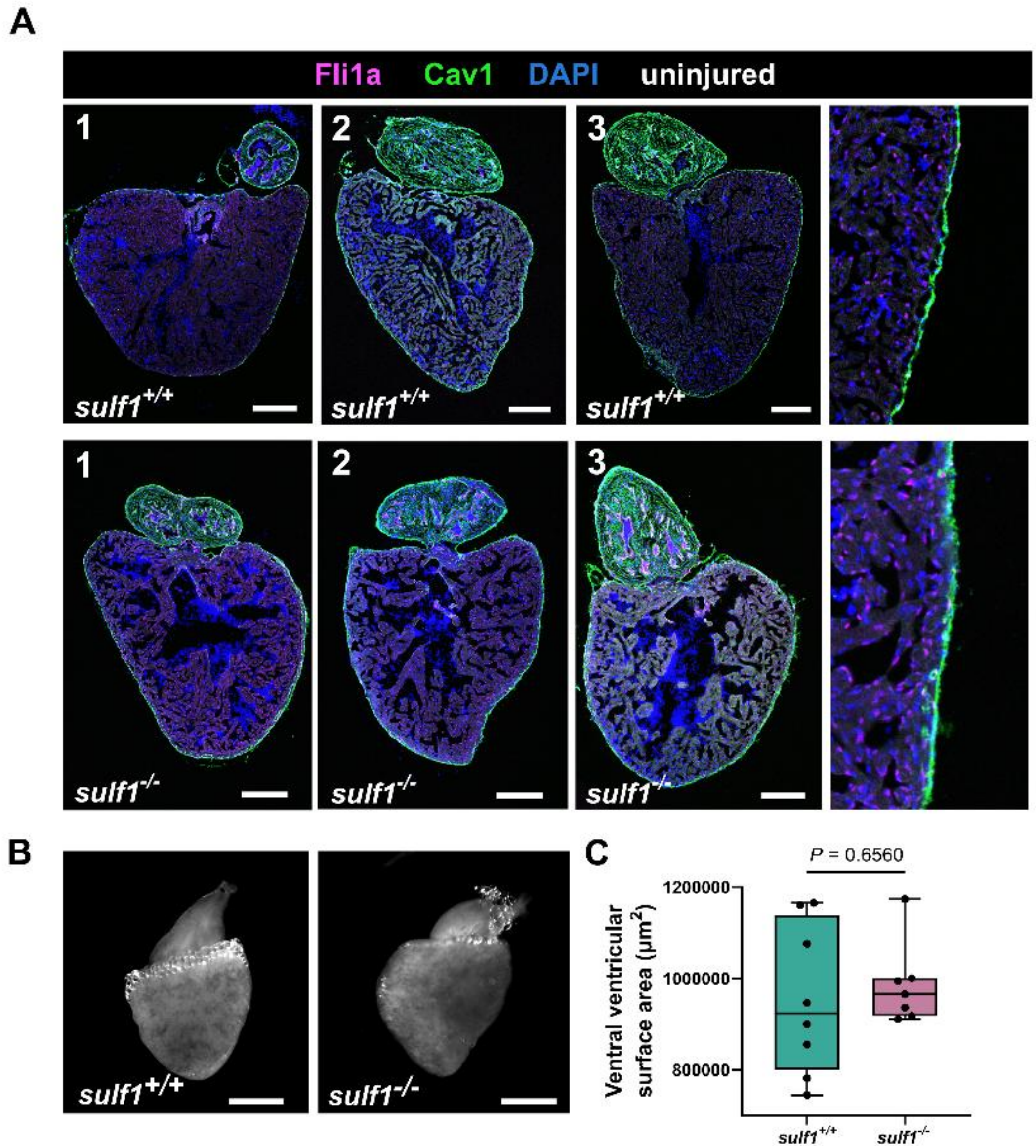

Appendix Figure S4: Epicardial morphology and ventricular surface area in *sulf1*$^{-/-}$.

A. *sulf1*$^{+/+}$ and *sulf1*$^{-/-}$ uninjured ventricular sections from three different hearts stained for Fli1a (ECs, magenta), Cav1 (epicardium, green) and DAPI (DNA, blue). Insets show high-

magnification images of the ventricular wall. Scale bars: 100 µm. **B.** Whole-mount *sulf1*$^{+/+}$ and *sulf1*$^{-/-}$ ventricles from 25-mm -long zebrafish. Scale bars: 500 µm. **C.** Surface area of ventricular surface ventrally oriented in 25-mm-long *sulf1*$^{+/+}$ (n=8) and *sulf1*$^{-/-}$ (n=7) zebrafish. Statistical tests: Student's t-test. For the boxplot, the center line indicates the median; the box bounds represent the 25th and 75th percentiles; the whiskers extend to the most extreme data points; and each point represents an independent measurement.

Appendix Table S1: Primer sequences

**Gene**	**Forward primer**	**Reverse primer**
	**PCR genotyping**	
sulf1-FLD-out primers	CCAACCTTCTGATTCGCGTTC	GTCTTGAATGTCCCTGGGCT
sulf1-FLD-in primers	CCAACCTTCTGATTCGCGTTC	ATGTTCCTCCGGTCACCTTG
sulf1-Exon3- primers	AGGTGATGAACAAAACCCGCA	TGGACGTACTTCCCCGTCA
	**qPCR**	
rpl13a	TCTGGAGGACTGTAAGAGGTATG	AGACGCACAATCTTGAGAGCAG
sulf1	AGGTGATGAACAAAACCCGCA	TGGACGTACTTCCCCGTCA
sulf2a	GCTGCCAACAGAGGACAGAT	TTGAGGTTGAGGGGACGGTA
sulf2b	CTGCCAGCCCAAGAGTTACA	ACTTTGATGGAGGAGGGCAC
ptx3a	GATCGGTACGAGCAGCATGA	TAAAGGCTCAGCTGTTCCCG
postnb	CTTCTCGAAGCGGAGGTGAG	TGAGGAGACGCTTGTTGACC
tnfa	TTCACGCTCCATAAGACCCA	CCGTAGGATTCAGAAAAGCG
il6	CGTAAAGAGTCTCCTTGGCG	GCCGTCATGTTCACCATCTC
il1b	GCACATCAAACCCCAATCCA	GCACTGAATCCACCACGTTC
ccl20a.3	AATGCTGTCATATTCCACACAAAC	CCATCACCCAGCCTTTGGAC
cdaa	GCATGTTACACCGCTGGATTG	GATCTTCAAGGTCACTGGCGA
