# Supplementary material for: Sulf1 regulates inflammation via modulation of TGF-β dependent epicardial activation
Source: EMBO Rep. 2026 Aug 27;27(19):5694–719. doi: 10.1038/s44319-026-00910-5 (PMC13649710; doi:10.1038/s44319-026-00910-5)
Supplement: Supplementary file 3 [file 44319_2026_910_MOESM2_ESM.pdf]

EMBO reports

Sulf1 regulates inflammation via modulation of TGF-β dependent epicardial activation

Gursimran Kaur Bajwa, Gülsüm Kayman Kürekçi, and Rubén Marín-Juez

Corresponding author(s): Rubén Marín-Juez (ruben.marin.juez.hsj@ssss.gouv.qc.ca)

Review Timeline:

Submission Date:	21st Oct 25
Editorial Decision:	19th Dec 25
Revision Received:	19th May 26
Editorial Decision:	6th Jul 26
Revision Received:	10th Jul 26
Accepted:	3rd Aug 26

Editor: Kurt Weir

Transaction Report:

(Note: With the exception of the correction of typographical or spelling errors that could be a source of ambiguity, letters and reports are not edited. Depending on transfer agreements, referee reports obtained elsewhere may or may not be included in this compilation. Referee reports are anonymous unless the Referee chooses to sign their reports.)

1st Editorial Decision **19th Dec 2025**

Dear Dr. Marín-Juez

Thank you for the submission of your research manuscript to our journal. We have now received the full set of referee reports that is copied below.

As you have seen, the referees acknowledge that the findings are interesting and that the conclusions are overall supported by the data presented but they also raise a number of concerns and have suggestions how to further strengthen the data, particularly greater characterization of uninjured Sulf1 mutant hearts and more direct evidence of Sulf1 regulation of TGFb signalling.

Given these constructive comments, we would like to invite you to revise your manuscript with the understanding that the referee concerns (as detailed above and in their reports) must be fully addressed and their suggestions taken on board. Please address all referee concerns in a complete point-by-point response. Acceptance of the manuscript will depend on a positive outcome of a second round of review. It is EMBO Reports policy to allow a single round of revision only and acceptance or rejection of the manuscript will therefore depend on the completeness of your responses included in the next, final version of the manuscript.

We realize that it is difficult to revise to a specific deadline. In the interest of protecting the conceptual advance provided by the work, we recommend a revision within 3 months (March 19th). Please discuss the revision progress ahead of this time with the editor if you require more time to complete the revisions.

I am also happy to discuss the revision further via e-mail or a video call, if you wish.

You can either publish the study as a short report or as a full article. For short reports, the revised manuscript should not exceed 27,000 characters (including spaces but excluding materials & methods and references) and 5 main plus 5 expanded view figures. The results and discussion sections must further be combined, which will help to shorten the manuscript text by eliminating some redundancy that is inevitable when discussing the same experiments twice. For a normal article there are no length limitations, but it should have more than 5 main figures and the results and discussion sections must be separate. In both cases, the entire materials and methods must be included in the main manuscript file.

================
IMPORTANT NOTE:

We perform an initial quality control of all revised manuscripts before re-review. Your manuscript will FAIL this control and the handling will be delayed IN CASE the following APPLIES:

1) A data availability section providing access to data deposited in public databases is missing. If you have not deposited any data, please add a sentence to the data availability section that explains that.

2) Your manuscript contains statistics and error bars based on n=2. Please use scatter blots in these cases. No statistics should be calculated if n=2.

When submitting your revised manuscript, please carefully review the instructions that follow below. Failure to include requested items will delay the evaluation of your revision.

================

When submitting your revised manuscript, we will require:

1) a .docx formatted version of the manuscript text (including legends for main figures, EV figures and tables). Please make sure that the changes are highlighted to be clearly visible.

2) individual production quality figure files as .eps, .tif, .jpg (one file per figure).
Please download our Figure Preparation Guidelines (figure preparation pdf) from our Author Guidelines pages https://www.embopress.org/page/journal/14693178/authorguide for more info on how to prepare your figures.

3) a .docx formatted letter INCLUDING the reviewers' reports and your detailed point-by-point responses to their comments. As part of the EMBO Press transparent editorial process, the point-by-point response is part of the Review Process File (RPF), which will be published alongside your paper.

4) a complete author checklist, which you can download from our author guidelines

(<https://www.embopress.org/page/journal/14693178/authorguide>). Please insert information in the checklist that is also reflected in the manuscript. The completed author checklist will also be part of the RPF.

5) Please note that all corresponding authors are required to supply an ORCID ID for their name upon submission of a revised manuscript (<https://orcid.org/>). Please find instructions on how to link your ORCID ID to your account in our manuscript tracking system in our Author guidelines
(<https://www.embopress.org/page/journal/14693178/authorguide#authorshipguidelines>)

6) We replaced Supplementary Information with Expanded View (EV) Figures and Tables that are collapsible/expandable online. A maximum of 5 EV Figures can be typeset. EV Figures should be cited as 'Figure EV1, Figure EV2" etc... in the text and their respective legends should be included in the main text after the legends of regular figures.

- For the figures that you do NOT wish to display as Expanded View figures, they should be bundled together with their legends in a single PDF file called *Appendix*, which should start with a short Table of Content. Appendix figures should be referred to in the main text as: "Appendix Figure S1, Appendix Figure S2" etc. See detailed instructions regarding expanded view here:
<https://www.embopress.org/page/journal/14693178/authorguide#expandedview>

- Additional Tables/Datasets should be labeled and referred to as Table EV1, Dataset EV1, etc. Legends have to be provided in a separate tab in case of .xls files. Alternatively, the legend can be supplied as a separate text file (README) and zipped together with the Table/Dataset file.

7) Before submitting your revision, primary datasets (and computer code, where appropriate) produced in this study need to be deposited in an appropriate public database (see <
https://www.embopress.org/page/journal/14693178/authorguide#dataavailability>).

Please remember to provide a reviewer password if the datasets are not yet public.

The accession numbers and database should be listed in a formal "Data Availability " section (placed after Materials & Method) that follows the model below (see also < https://www.embopress.org/page/journal/14693178/authorguide#dataavailability>). Please note that the Data Availability Section is restricted to new primary data that are part of this study.

Data availability

The datasets (and computer code) produced in this study are available in the following databases:

- RNA-Seq data: Gene Expression Omnibus GSE46843 (https://www.ncbi.nlm.nih.gov/geo/query/acc.cgi?acc=GSE46843)
- [data type]: [name of the resource] [accession number/identifier/doi] ([URL or identifiers.org/DATABASE:ACCESSION])

*** Note - All links should resolve to a page where the data can be accessed. ***

8) At EMBO Press we ask authors to provide source data for the main manuscript figures. You will receive a separate email with instructions for providing source data with your revised manuscript, including how to upload and organize the files.

Additional information on source data and instruction on how to label the files are available
<https://www.embopress.org/page/journal/14693178/authorguide#sourcedata>

9) The journal requires a statement specifying whether or not authors have competing interests (defined as all potential or actual interests that could be perceived to influence the presentation or interpretation of an article). In case of competing interests, this must be specified in your disclosure statement. Further information: https://www.embopress.org/competing-interests

10) Figure legends and data quantification:
The following points must be specified in each figure legend:

- the name of the statistical test used to generate error bars and P values,
- the EXACT p-values,
- the number (n) of independent experiments (please specify technical or biological replicates) underlying each data point,
- the nature of the bars and error bars (s.d., s.e.m.)

- If the data are obtained from n {less than or equal to} 5, show the individual data points in addition to the SD or SEM.
- If the data are obtained from n {less than or equal to} 2, use scatter blots showing the individual data points.

Discussion of statistical methodology can be reported in the materials and methods section, but figure legends should contain a basic description of n, P and the test applied.

See also the guidelines for figure legend preparation:
https://www.embopress.org/page/journal/14693178/authorguide#figureformat

- Please also include scale bars in all microscopy images and define their size in the legend, not the image.

11) Our journal encourages inclusion of *data citations in the reference list* to directly cite datasets that were re-used and obtained from public databases. Data citations in the article text are distinct from normal bibliographical citations and should directly link to the database records from which the data can be accessed. In the main text, data citations are formatted as follows: "Data ref: Smith et al, 2001" or "Data ref: NCBI Sequence Read Archive PRJNA342805, 2017". In the Reference list, data citations must be labeled with "[DATASET]". A data reference must provide the database name, accession number/identifiers and a resolvable link to the landing page from which the data can be accessed at the end of the reference. Further instructions are available at <https://www.embopress.org/page/journal/14693178/authorguide#referencesformat>.

12) All Materials and Methods need to be described in the main text using our 'Structured Methods' format. According to this format, the Methods section includes a Reagents and Tools Table (listing key reagents, experimental models, software and relevant equipment and including their sources and relevant identifiers) followed by a Methods and Protocols section describing the methods, ideally using a step-by-step protocol format. The aim is to facilitate adoption of the methodologies across labs. Please download and fill our Reagents and Tools Table template (.docx), which you can find in our author guidelines: https://www.embopress.org/page/journal/14693178/authorguide#structuredmethods.
When submitting your revised manuscript, please do not include the Reagents and Tools Table in the Methods section of the manuscript but upload it as a separate file choosing the file type "Reagent Table".
An example of a Method paper with Structured Methods can be found here:
https://www.embopress.org/doi/10.15252/msb.20178071.

13) As part of the EMBO publication's Transparent Editorial Process, EMBO Reports publishes online a Review Process File to accompany accepted manuscripts. This File will be published in conjunction with your paper and will include the referee reports, your point-by-point response and all pertinent correspondence relating to the manuscript.

You are able to opt out of this by letting the editorial office know (emboreports@embo.org). If you do opt out, the Review Process File link will point to the following statement: "No Review Process File is available with this article, as the authors have chosen not to make the review process public in this case."

We would also welcome the submission of cover suggestions, or motifs to be used by our Graphics Illustrator in designing a cover.

I look forward to seeing a revised form of your manuscript when it is ready and please let me know if you have questions or comments regarding the revision.

You can use this link to submit your revision: https://embor.msubmit.net/cgi-bin/main.plex

Yours sincerely,

Kurt Weir
Editor
EMBO Reports

Referee #1:

In this study, Bajwa et al. report that cardiac injury leads to increased endo-sulfatase Sulf1 expression in epicardial cells during zebrafish heart regeneration. Using genetic loss-of-function and tissue-specific gain-of-function approaches, the authors show that Sulf1 contributes to regenerative outcomes by promoting epicardial activation and modulating inflammatory responses, in part through TGFβ signaling within epicardial cells.

While the study is of interest and suggests a potential new regenerative mechanism, the authors should address the following points thoroughly before the manuscript can be considered further for publication.

Major points:
1) The primary weakness of the study is the lack of a clear mechanistic understanding of Sulf1 function in the epicardium. The authors propose that Sulf1 regulates TGFb signaling in epicardial cells, and this appears to be associated, at least in part, with reduced transcription of tgfb ligands (Fig. 4F). However, this proposed mechanism is not readily reconciled with the known role of Sulf1 in fine-tuning HSPG-dependent signaling. Further mechanistic investigation would be needed to delineate how Sulf1

regulates TGFb signaling in the epicardium.

2) The histological evidence for epicardial migration defects appears weak (Fig. 2F, G), as the observations may vary depending on the sections selected for analysis. A more direct evaluation of epicardial migration could be achieved using an explant culture approach. In addition, the authors should include proliferation analyses to more comprehensively characterize epicardial activation in Fig. 2.

3) CM dedifferentiation and proliferation were reduced in Sulf1-KO ventricles (Fig. 3). The underlying mechanism for this defect would need to be described.

4) Based on their CellChat analysis, the authors conclude that Sulf1 deficiency rewires ligand-receptor interactions within the TGFb signaling pathway in the epicardium (Fig. 4H), resulting in the emergence of the following combinations in the mutant: tgfb3 - (acvr1ba + tgfbr2b), tgfb2 - (acvr1ba + tgfbr2b), and tgfb1a - (acvr1ba + tgfbr2b). However, these inferred interactions seem difficult to reconcile with the finding that the expression of all three ligand genes (tgfb3, tgfb2, tgfb1a) is reduced in Sulf1-KO hearts (Fig. 4F). Clarification of this discrepancy would be appreciated.

5) The gain-of-function tool (sulf1 OE) should be validated in the adult context rather than in embryos (Fig. S5). In particular, the authors should provide evidence demonstrating Sulf1 expression in the adult epicardium of the transgenic hearts.

6) Demonstrating enhanced migration and increased tgfb ligand expression in the Sulf1-OE epicardium would further strengthen and substantiate the authors' conclusions.

Minor points:
1) Fig. 1C: The DAPI signal is shown in white, which visually overlaps with the double-positive (magenta + green) cells. The authors should consider using a different color for DAPI to avoid this ambiguity.

2) Fig. 2C: aldh1a2 expression also appears reduced in the KO ventricle. If this represents a bona fide phenotype, the authors should describe and discuss it.

3) Fig. 4C: The colors used for the group quantification columns do not appear to correspond to those assigned to the cell populations on the right (there seem to be additional colors present in the columns). Clarification of this mismatch would be helpful.

4) Fig. 6E: Please verify whether this figure is cited in the text.

Referee #2:

This is a nice study providing novel mechanistic insight into zebrafish heart regeneration, in particular into the role of the epicardium. It combines genetic manipulations and single cell sequencing and generally provides data of high quality (with the exceptions listed below). The phenotypes found in the sulf1 mutant are quite stunning, and the hypothesis put forward by the authors that epicardial TGFb signaling, which depends on sulf1 is a major regulator of many aspects of heart regeneration, very interesting. However, I find some of the conclusions not sufficiently backed by data. Yet, if the authors can address the issues detailed below and further support their conclusions, I think the work will be of great interest to the readership of EMBO Rep.

Major:

1) Images are generally very pixelated in the PDF, which limits the reader's ability to truly assess author's claims about expression patterns.

2) Reference 34: I recommend treating non-peer review data with caution and to not use language like "recent studies have shown" when referring to preprints.

3) Figure 1B: both the Aldh and the sulf1 signals are hardly visible, thus the claim that sulf1 is expressed in aldh1a+ cells needs better supporting data.

4) Figure1C: While the signals in the insets are convincing, it appears that a lot of green signal is present in the myocardium. Please comment, since the results text indicates that expression is specific to the epicardium.

5) Figure 1 D, E: figure legends indicate that white vs yellow arrowheads should point to different cell populations, but they appear to point to the same locations. Also, some arrows do not point to any obvious signals. Please clarify. More troubling, in 1E it looks as if the GFP and Aldh1a2 signals are largely non-overlapping. Authors should back their claim that there is co-expression with better data. I recommend showing the overlap of green and magenta channels.

6) sulf1 mutants: obviously these are not embryonic lethal; yet, authors need to provide information about whether the homozygotes have any other detectable / obvious phenotypes. Do they survive at expected Mendelian ratios? Are they fertile? More importantly, do their hearts develop normally (authors cite the preprint claiming that sulf1 is required for heart development). Are adult hearts of normal size and morphology and is the epicardium normal (Cav1 expression)?

7) control wild-type siblings: methods do not state whether wild-type siblings of mutants were subjected to same conditions as mutants as much as possible. Heart regeneration can heavily be influenced by factors like fish nutrition, size, or density they are kept at during regeneration. Ideally, all experiments should have been performed on fish prior to genotyping (agnostic to genotype). If that was not possible, wild-types and mutants should have been raised together and only be separated shortly prior to heart injury. After injury and prior to heart harvesting, the fish should be kept at the same density. Have the authors applied these principles?

8) Fig 2C: statements about differences like this must be backed by quantitative data or at least some measure of reproducibility. That is, Aldh1 signals should be quantified.

9) Fig. 3A: presumably also the overall number of Cav1+ cells is reduced in the mutant? Authors should report the full panel of data that is number of Cav1+ Smad3- cells as well. Image also indicates that Smad3 in other cells in the wound is reduced in the mutants. Is this true?

10) Repetition of experiments: While some phenotypes are shown by different means (e.g. the reduction of epicardial activation in Figure 2), where the data for aldh2, ptx3 and the tcf1 transgene hopefully (at least partially) are derived from different fish, such that the combined n number is relatively high, other experiments appear to have been performed only once. With n=5 or 6 this is critical. I strongly recommend repeating all experiments in Figure 3 with another set of fish. Same is true for Fig. 6A/B, where n=3 is way too low; also Fig. 7B (n=4), and Fig. 7G-H, which also should be truly quantified.

11) Figure 3G, J: why do authors show pseudo-quantified data and not truly quantified measures of scar sizes?

12) Figure 7F: not convincing. Needs to be backed by quantification.

13) As is, experimental evidence for the crucial function of TGFb signaling downstream of sulf1 is sparse. A very nice addition would be to test whether activation of TGFb signaling can rescue the sulf1 mutant phenotypes. I am aware that this might require a transgenic line for TGFb overexpression, and if the authors have to create it this takes a long time. Yet, maybe they have already thought about this and can add such data?

Minor:

1) sulf1 gene cartoons in Fig. 2 and S2: why is there an exon depicted 5' of the box that is labelled black (5' UTR)? If it's a sulf1 exon, it also must be part of the 5' UTR...

2) Fig. 2G: y-axis label must be wrong, since it does not make sense to show % of > 100

3) Line 166: "did not interfere with"?

Referee #3:

Here, the authors investigate the role of Sulf1, a gene encoding a sulfatase that affects heperan sulphate proteoglycans, during zebrafish heart regeneration. The authors describe that sulf1 is expressed in epicardial cells upon injury and generated a loss-of-function mutant which is affected in its regeneration response. The authors show that sulf1 affects TGF-beta signaling, leading to an alteration in the immune response after injury, which can explain the observed reduction in CM proliferation and impaired scar regression.

Overall, this is a very important study as it shows the complexity of cardiac regeneration in which various cell types interact to restore the injured tissue. The experiments are well executed, and the authors have generated state-of-the-art genetic tools to study the role of sulf1. The data is presented in a very clear manner, and the manuscript is well written. There are some issues related to the sulf1 mutant hearts pre-injury and validation of the scRNAseq data that need to be addressed by the authors.

Major issues

1. Mouse study that is published as a preprint shows that sulf1 is expressed in the epicardium and that this is essential for cardiac development, as sulf1 mutants have defects in formation of the epicardium. Here the authors describe a defect in the response of the epicardial cells to a cardiac injury. What is not addressed here is whether the sulf1 mutant hearts already have a defect in epicardium formation before the injury is applied. The authors therefore should address carefully whether epicardium formation is affected or not in sulf1 mutants in the absence of an injury. This can be done by including some stainings with

epicardial markers on uninjured hearts as well as a careful examination of the single-cell transcriptome data from uninjured wt and sulf1 mutant hearts. Some of these data are presented in Fig. S3, but these data are not described in the manuscript.
2. Linked to the previous comment, the scRNAseq data in S3 shows a strong reduction in ECs in uninjured sulf1 mutant hearts. The authors do not elaborate on this as far as I can see. The authors should discuss the implementation of this reduction in ECs on regeneration outcome in sulf1 mutants.
3. For their conclusions about changes in gene expression in sulf1 mutants the authors rely on the scRNAseq data. Based on the M&M the scRNAseq experiment was performed once without an independent replicate experiment. Therefore the authors should replicate and confirm some of their major findings and conclusions about altered gene expression using another technique such as by qPCR or ISH.
4. Authors write in line 306: 'Collectively, these findings indicate that Sulf1 regulates TGF-β signaling-mediated epicardial activation and EPDC regeneration which in turn is required for efficient fibrosis resolution.'. The conclusion that TGF-beta signaling is responsible for the pertained scar is a stretch as many studies have shown that reducing cardiomyocyte proliferation also affects the resolution of the temporal fibrosis. Therefore the observed effects on fibrosis could well be very indirect. The authors should be careful when drawing conclusions about fibrosis without further investigating the underlying mechanisms.

Minor issues
5. Authors should also cite original research papers when referring to published work rather than only citing literature reviews. For example in line 290: 'After cardiac injury, CMs dedifferentiate and re-enter the cell cycle to rebuild the lost myocardium64,65.' Authors should include pmid 20336144 and 20336145. There are more examples of this throughout the manuscript.

Dr. Kurt Weir
Editor, *EMBO Reports*

May 19, 2026

Dear Dr. Weir,

Thank you for your help with our manuscript entitled "**Sulf1 regulates inflammation via modulation of TGF-β dependent epicardial activation**".

We also want to thank the reviewers for their encouraging and positive remarks as well as for their thoughtful and helpful suggestions to improve our study. We have now addressed all reviewers' comments and successfully carried out the reviewers' suggestions for additional experiments, which have helped make this paper stronger.

The detailed responses to the reviewers' queries are listed below.

With best regards,

Rubén Marín-Juez

Response to reviewers' comments:

Referee #1:
In this study, Bajwa et al. report that cardiac injury leads to increased endo-sulfatase Sulf1 expression in epicardial cells during zebrafish heart regeneration. Using genetic loss-of-function and tissue-specific gain-of-function approaches, the authors show that Sulf1 contributes to regenerative outcomes by promoting epicardial activation and modulating inflammatory responses, in part through TGFβ signaling within epicardial cells.

While the study is of interest and suggests a potential new regenerative mechanism, the authors should address the following points thoroughly before the manuscript can be considered further for publication.

We thank the reviewer for their supportive and constructive comments.

Major points:
1) The primary weakness of the study is the lack of a clear mechanistic understanding of Sulf1 function in the epicardium. The authors propose that Sulf1 regulates TGFb signaling in epicardial cells, and this appears to be associated, at least in part, with reduced transcription of tgfb ligands (Fig. 4F). However, this proposed mechanism is not readily reconciled with the known role of Sulf1 in fine-tuning HSPG-dependent signaling. Further mechanistic investigation would be needed to delineate how Sulf1 regulates TGFb signaling in the epicardium.

As rightly indicated by the reviewer, our data show that the expression of different TGF-β ligands is reduced in *sulf1* mutant EPDCs. This phenotype could be due to reduced TGF-β signaling itself since TGF-β signaling maintains a positive feedback mechanism wherein active TGF-β signaling induces transcription of its ligands (PMID: 39311474, 29228156). Since TGF-β signaling is attenuated in the mutant, we believe this reduction is in turn leading to reduced expression of TGF-β ligands in *sulf1*$^{-/-}$.

Sulf1 selectively removes 6-*O*-sulfate groups from HS chains thereby modulating ligand interactions and fine-tuning signaling pathways. Currently, it is technically not possible to demonstrate how lack of Sulf1 limits TGF-β ligand availability. To further address this mechanism, we have now utilised Alcian Blue staining at low pH (pH~1.0) to assess changes in glycosaminoglycan (GAG) sulfation (PMID: 41917030, 21056656). Indeed, we find that GAG sulfation increased in the mutant epicardium (Figure EV1A). These data point towards altered HSPG sulfation in absence of *sulf1* which in turn has been shown to limit TGF-β ligand availability (PMID: 25503294, 35409127).

We have now also added data using another newly generated *sulf1* allele (*sjr10)* where we mutated the highly conserved sulfatase domain, aiming to alter its enzymatic activity (Figure EV1B,C). Consistent with our previous findings, in this new mutant we also observe reduced pSmad3+ cells compared to their wild-type siblings at 4 dpci (Figure EV1D,E). These new data further support that epicardial TGF-β signaling is reduced in the absence of Sulf1.

In view of these new and previous findings, we believe that it is reasonable to hypothesize that *sulf1* mutants have impaired HSPG sulfation levels that might affect TGF-β ligand availability, in turn leading to reduced signaling.

These limitations as well as these new data are now included in the revised manuscript.

2) The histological evidence for epicardial migration defects appears weak (Fig. 2F, G), as the observations may vary depending on the sections selected for analysis. A more direct evaluation of epicardial migration could be achieved using an explant culture approach. In addition, the authors should include proliferation analyses to more comprehensively characterize epicardial activation in Fig. 2.

We thank the reviewer for pointing this out. Indeed, we intentionally refrained from referring to “migration” in the manuscript as we recognized that the lack of live imaging data precludes a definitive conclusion regarding migratory defects. Consequently, we characterized the observed phenotype as 'EPDC expansion defects' to more accurately reflect our data. Additionally, to ensure reproducibility and minimize bias, all analyses were restricted to injuries of comparable sizes (approximately 20% of the ventricle). For quantification, at least three non-superficial, non-consecutive sections per heart were analyzed.

As suggested by the reviewer, we have now added data using epicardial explant cultures from wild-type and *sulf1* mutant hearts (Figure 2H,I). The distance covered by *sulf1*

mutant EPDCs was significantly reduced compared with that of wild-type explants, indicating defective migration.

In addition, we have now analyzed potential proliferation defects in mutant EPDCs. We have interrogated our scRNA-seq dataset and find no obvious differences in the expression levels of *top2a* and *mcm2*, both canonical proliferation markers (Figure EV2F). Further analyses of cell cycle regulators such as *cdc25b* and *ccnd1* showed reduced expression in mutants (Figure EV2F). These data suggest that mutant epicardial cells might experience alterations in cell cycle progression. These new data are now included in the revised manuscript.

3) CM dedifferentiation and proliferation were reduced in Sulf1-KO ventricles (Fig. 3). The underlying mechanism for this defect would need to be described.

Previous studies have shown that TGF-β/Smad3 signaling regulates CM regeneration by enabling an EMT-like response in epicardial cells (PMID: 22513374, 34055811). Similarly, there is increasing evidence showing that exacerbated inflammation can hinder CM regeneration (PMID: 40554763, 30928664). Therefore, it is possible that the observed reduction in CM proliferation and dedifferentiation occurs as a consequence of the defects in epicardial TGF-β signaling and inflammation in the mutant heart. We have now discussed this point in the revised manuscript (lines 360-361 and 391-393).

4) Based on their CellChat analysis, the authors conclude that Sulf1 deficiency rewires ligand-receptor interactions within the TGFb signaling pathway in the epicardium (Fig. 4H), resulting in the emergence of the following combinations in the mutant: tgfb3 - (acvr1ba + tgfbr2b), tgfb2 - (acvr1ba + tgfbr2b), and tgfb1a - (acvr1ba + tgfbr2b). However, these inferred interactions seem difficult to reconcile with the finding that the expression of all three ligand genes (tgfb3, tgfb2, tgfb1a) is reduced in Sulf1-KO hearts (Fig. 4F). Clarification of this discrepancy would be appreciated.

We apologise for the lack of clarity. This result is likely explained by the fact that the expression of *tgfbr2b* is slightly elevated in *sulf1* mutant EPDCs. The CellChat analysis predicts ligand-receptor interactions based on combined expression levels of different ligands and receptors. Therefore, the predicted interactions might be a result of this slight elevation in *tgfbr2b* expression in mutants (Figure EV2E). This possibility is now discussed in the revised manuscript.

5) The gain-of-function tool (sulf1 OE) should be validated in the adult context rather than in embryos (Fig. S5). In particular, the authors should provide evidence demonstrating Sulf1 expression in the adult epicardium of the transgenic hearts.

We have now included qPCR data for *sulf1* in *sulf1OE* hearts at 4 dpci, confirming upregulation of *sulf1* expression (Appendix Figure S3C). In addition, we have now included HCR stainings showing increased expression of *sulf1* in the regenerating EPDCs (Appendix Figure S3D).

6) Demonstrating enhanced migration and increased tgfb ligand expression in the Sulf1-OE epicardium would further strengthen and substantiate the authors' conclusions.

We have now added epicardial explant data for *sulf1*OE hearts and find that overexpression of *sulf1* in EPDCs stimulates epicardial outgrowth in the explants (Figure 7H,I). In *sulf1* mutants, we observe expression changes across multiple TGF-β ligands as well as receptors, suggesting that the pathway is broadly affected. Therefore, for our analyses we chose to assess pSmad3 levels as a readout for TGF-β signaling activation. Our data show that induction of *sulf1* in EPDCs increases the number of pSmad3+ cells, indicating increased TGF-β signaling.

Minor points:
1) Fig. 1C: The DAPI signal is shown in white, which visually overlaps with the double-positive (magenta + green) cells. The authors should consider using a different color for DAPI to avoid this ambiguity.

We have now updated the figure to avoid the observed ambiguity.

2) Fig. 2C: aldh1a2 expression also appears reduced in the KO ventricle. If this represents a bona fide phenotype, the authors should describe and discuss it.

The data are described and discussed in the manuscript (lines 131-131 and 348-350).

3) Fig. 4C: The colors used for the group quantification columns do not appear to correspond to those assigned to the cell populations on the right (there seem to be additional colors present in the columns). Clarification of this mismatch would be helpful.

We have now replaced the graph with a more clearly defined color scheme for clarity.

4) Fig. 6E: Please verify whether this figure is cited in the text.

Thanks for pointing this out. We have now added this information in the revised manuscript.

Referee #2:

This is a nice study providing novel mechanistic insight into zebrafish heart regeneration, in particular into the role of the epicardium. It combines genetic manipulations and single cell sequencing and generally provides data of high quality (with the exceptions listed below). The phenotypes found in the sulf1 mutant are quite stunning, and the hypothesis put forward by the authors that epicardial TGFb signaling, which depends on sulf1 is a major regulator of many aspects of heart regeneration, very interesting. However, I find some of the conclusions not sufficiently backed by data. Yet, if the authors can address the issues detailed below and further support their conclusions, I think the work will be of great interest to the readership of EMBO Rep.

We thank the reviewer for their supportive and constructive comments.

Major:

1) Images are generally very pixelated in the PDF, which limits the reader's ability to truly assess author's claims about expression patterns.

We apologize for the poor image quality. It is unfortunately the constraint of the file size that can be uploaded to the portal. We have now revised all our figures and provided higher resolution and/or more clear images to help the assessment of our data.

2) Reference 34: I recommend treating non-peer review data with caution and to not use language like "recent studies have shown" when referring to preprints.

We have now revised the text accordingly when referring to preprints.

3) Figure 1B: both the Aldh and the sulf1 signals are hardly visible, thus the claim that sulf1 is expressed in aldh1a+ cells needs better supporting data.

We have now updated the panel with figures with improved resolution (Figure 1D,G and H).

4) Figure1C: While the signals in the insets are convincing, it appears that a lot of green signal is present in the myocardium. Please comment, since the results text indicates that expression is specific to the epicardium.

As rightly pointed out by the reviewer, the zebrafish myocardium has strong background signal which possess a significant limitation when performing histological analyses. HCR specific signal appears as distinct bright puncta labelling individual RNA molecules while background signal looks like low intensity diffused signal. Based on this feature, we can confidently identify epicardial specific *sulf1* expression. Following the reviewer's suggestion, we have now substituted the panel with a better and clearer representative figure (Figure 1C).

5) Figure 1 D, E: figure legends indicate that white vs yellow arrowheads should point to different cell populations, but they appear to point to the same locations. Also, some arrows do not point to any obvious signals. Please clarify. More troubling, in 1E it looks as if the GFP and Aldh1a2 signals are largely non-overlapping. Authors should back their claim that there is co-expression with better data. I recommend showing the overlap of green and magenta channels.

We apologize for the confusion. We now updated the EGFP and Cav1 co-staining (Figure 1E,F) and the EGFP and Aldh1a2 co-staining (Figure 1G,H). We have also added separate green and magenta channels along with the overlap for better co-localisation. We have now used single color arrowheads to indicate cells that are EGFP+ Cav1+ (Figure 1E,F) and EGFP+ Aldh1a2+ (Figure 1G,H).

6) sulf1 mutants: obviously these are not embryonic lethal; yet, authors need to provide

information about whether the homozygotes have any other detectable / obvious phenotypes. Do they survive at expected Mendelian ratios? Are they fertile? More importantly, do their hearts develop normally (authors cite the preprint claiming that sulf1 is required for heart development). Are adult hearts of normal size and morphology and is the epicardium normal (Cav1 expression)?

Adult homozygous mutants display no gross morphological defects; they survive with expected Mendelian ratios and are fertile. We have now quantified area of ventral ventricular surface of adult *sulf1* mutant hearts compared to their wild-type siblings, which showed no significant difference between the two groups (Appendix Figure S4B,C). In addition, we have now also analyzed potential changes in epicardial morphology by Cav1 staining on sections of uninjured *sulf1* mutant and wild-type sibling hearts. We did not observe any gross morphological defects (Appendix Figure S4A). We have now added this information in the revised manuscript.

7) control wild-type siblings: methods do not state whether wild-type siblings of mutants were subjected to same conditions as mutants as much as possible. Heart regeneration can heavily be influenced by factors like fish nutrition, size, or density they are kept at during regeneration. Ideally, all experiments should have been performed on fish prior to genotyping (agnostic to genotype). If that was not possible, wild-types and mutants should have been raised together and only be separated shortly prior to heart injury. After injury and prior to heart harvesting, the fish should be kept at the same density. Have the authors applied these principles?

Indeed, these principles were applied. We have now added this information in the revised manuscript.

8) Fig 2C: statements about differences like this must be backed by quantitative data or at least some measure of reproducibility. That is, Aldh1 signals should be quantified.

We have now increased the number of samples used for HCR analysis to n = 7. We have quantified the proportion of samples displaying the phenotype as an indicator of consistency across biological replicates. In 6 out of 7 biological replicates, we observed reduced *aldh1a2* expression in the mutant epicardium. These data have been included in the revised manuscript.

Additionally, we have included here the expression data from scRNA-seq showing reduced expression of *aldh1a2* in injury-induced epicardial clusters (Figure A).

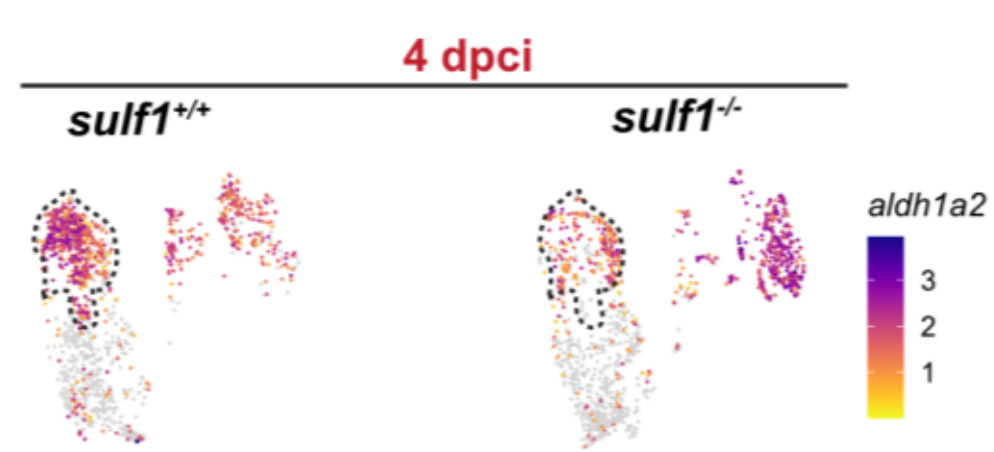

Figure A. UMAPs showing normalized expression of *aldh1a2* in wild-type and mutant EPDCs. Dotted line delineates injury-induced epicardial populations.

9) Fig. 3A: presumably also the overall number of Cav1+ cells is reduced in the mutant? Authors should report the full panel of data that is number of Cav1+ Smad3- cells as well. Image also indicates that Smad3 in other cells in the wound is reduced in the mutants. Is this true?

We thank the reviewer for this observation. We did not separately quantify the number of Cav1$^+$ cells, as this would be largely redundant with the *tcf21*:mCherry$^+$ epicardial cells quantification shown in Figure 2F,G, with both readouts reflecting the EPDC expansion phenotype.

To show reduced Cav1 expression in the mutants, we have now included expression data from our scRNA-seq showing reduced Cav1 expression in injury activated EPDCs in mutants (Figure B).

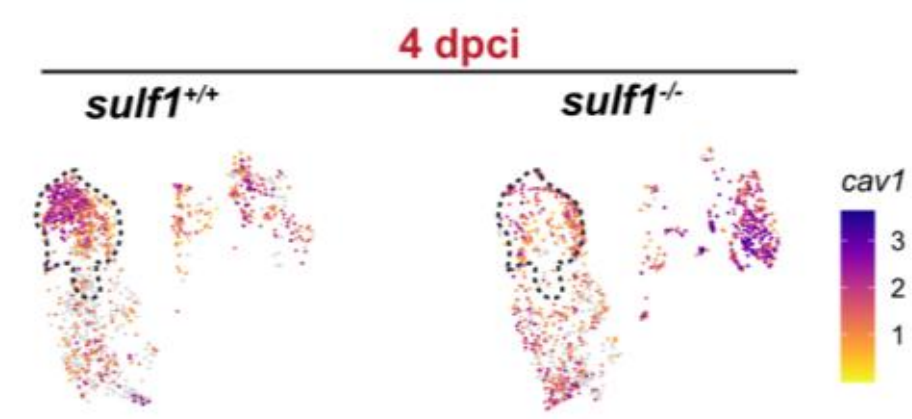

Figure B. UMAPs showing normalized expression of *cav1* in wild-type and mutant EPDCs. Dotted line delineates injury-induced epicardial populations.

We have also quantified pSmad3$^+$ cells in the intraventricular space and found that they are reduced in *sulf1* mutants at 4 dpci (Figure C).

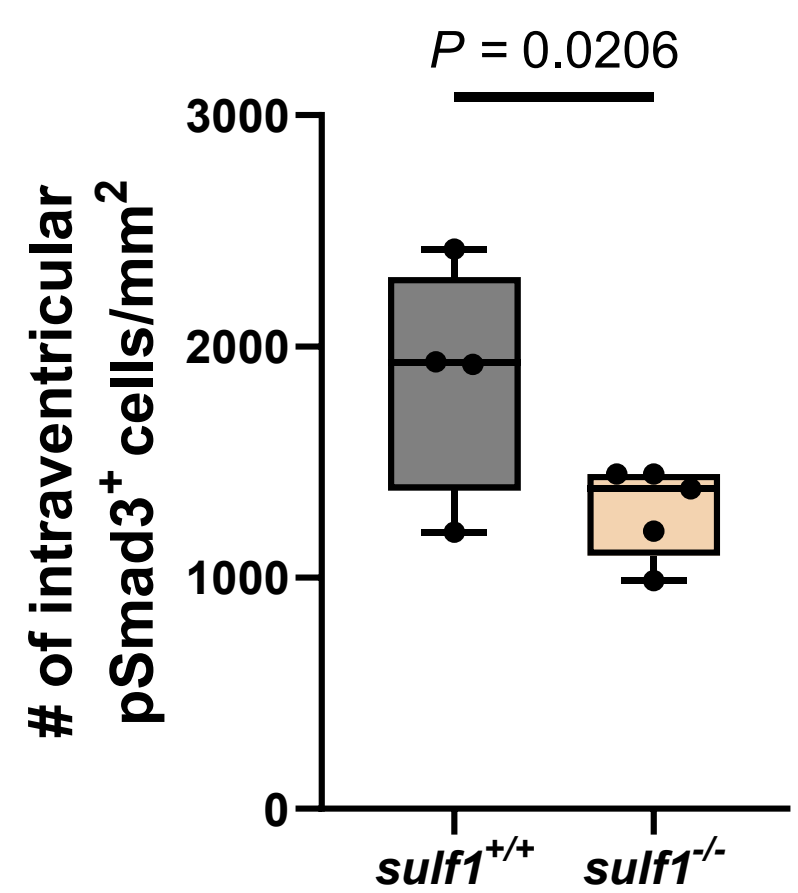

Figure C. Number of intraventricular pSmad3$^+$ cells per mm^2 injured area in *sulf1*$^{+/+}$ (n=4) and *sulf1*$^{-/-}$ (n=5) ventricles at 4 dpci

Importantly, the reduction in pSmad3$^+$ cells was more pronounced in the epicardium (Figure 3A,B).

10) Repetition of experiments: While some phenotypes are shown by different means (e.g. the reduction of epicardial activation in Figure 2), where the data for aldh2, ptx3 and the tcf1 transgene hopefully (at least partially) are derived from different fish, such that the combined n number is relatively high, other experiments appear to have been performed only once. With n=5 or 6 this is critical. I strongly recommend repeating all experiments in Figure 3 with another set of fish. Same is true for Fig. 6A/B, where n=3 is way too low; also Fig. 7B (n=4), and Fig. 7G-H, which also should be truly quantified.

We have now repeated these experiments and increased the number of animals/samples for requested figures. Additionally, we have now quantified data in Figures 3K, 3N and 7J.

11) Figure 3G, J: why do authors show pseudo-quantified data and not truly quantified measures of scar sizes?

Based on our experience, categorizing fibrotic areas in different groups is a more consistent way to reflect phenotype variability. This method of data representation has been extensively used in the field (PMID: 41068075, 41360282, 41776059, 39271818). Following the reviewer's suggestion, we have now also quantified fibrotic areas and included these quantifications in the revised figures (Figure 3L, 3O, 6I, 7L and 8H).

12) Figure 7F: not convincing. Needs to be backed by quantification.

We have now included qPCR data showing increased *ptx3a* expression in *sulf1OE* hearts at 4 dpci (Figure 7G).

13) As is, experimental evidence for the crucial function of TGFb signaling downstream of sulf1 is sparse. A very nice addition would be to test whether activation of TGFb signaling can rescue the sulf1 mutant phenotypes. I am aware that this might require a transgenic line for TGFb overexpression, and if the authors have to create it this takes a long time. Yet, maybe they have already thought about this and can add such data?

As the reviewer indicates, activation of TGF-β signalling to rescue *sulf1* mutant phenotypes would be a nice addition. Lines that would allow global or epicardial overexpression of specific TGF-β ligands in adult hearts are not available to us. These constraints limited our capacity to perform such experiments.

Minor:

1) sulf1 gene cartoons in Fig. 2 and S2: why is there an exon depicted 5' of the box that is labelled black (5' UTR)? If it's a sulf1 exon, it also must be part of the 5' UTR...

We thank the reviewer for bringing this to our attention. We have now made corrections in Figure 2, Figure EV1B and Appendix Figure S2A.

2) Fig. 2G: y-axis label must be wrong, since it does not make sense to show % of > 100
We apologize for this mistake. It has been corrected in the revised version of the manuscript.

3) Line 166: "did not interfere with"?
We have corrected the text in the revised manuscript.

Referee #3:

Here, the authors investigate the role of Sulf1, a gene encoding a sulfatase that affects heperan sulphate proteoglycans, during zebrafish heart regeneration. The authors describe that sulf1 is expressed in epicardial cells upon injury and generated a loss-of-function mutant which is affected in its regeneration response. The authors show that sulf1 affects TGF-beta signaling, leading to an alteration in the immune response after injury, which can explain the observed reduction in CM proliferation and impaired scar regression.

Overall, this is a very important study as it shows the complexity of cardiac regeneration in which various cell types interact to restore the injured tissue. The experiments are well executed, and the authors have generated state-of-the-art genetic tools to study the role of sulf1. The data is presented in a very clear manner, and the manuscript is well written. There are some issues related to the sulf1 mutant hearts pre-injury and validation of the scRNAseq data that need to be addressed by the authors.

We thank the reviewer for their supportive and constructive comments.

Major issues
1. Mouse study that is published as a preprint shows that sulf1 is expressed in the epicardium and that this is essential for cardiac development, as sulf1 mutants have defects in formation of the epicardium. Here the authors describe a defect in the response of the epicardial cells to a cardiac injury. What is not addressed here is whether the sulf1 mutant hearts already have a defect in epicardium formation before the injury is applied. The authors therefore should address carefully whether epicardium formation is affected or not in sulf1 mutants in the absence of an injury. This can be done by including some stainings with epicardial markers on uninjured hearts as well as a careful examination of the single-cell transcriptome data from uninjured wt and sulf1 mutant hearts. Some of these data are presented in Fig. S3, but these data are not described in the manuscript.

We have now added Cav1 staining data on sections from uninjured *sulf1* mutant and wild-type sibling hearts. We did not observe gross morphological defects on epicardial structure or ventricular size (Appendix Figure S4A).

To investigate the intact epicardium at the transcriptional level, we have analysed our scRNAseq dataset by subclustering EPDCs from uninjured wild-type and mutant hearts. Based on previously characterised markers, we identified two main epicardial populations: *podxl*$^+$ epithelial EPDCs (EPI 3,5) and *hapln1a*$^+$ mesenchymal EPDCs (EPI

1,2,6 and 7) in the uninjured hearts. Epi 4 cluster expressed both *podxl* and *hapln1a* (Figure Da-b). Although we could appreciate a trend suggesting reduced *podxl* expression in some mutant clusters, we did not observe clear differences in the expression levels of either gene (Figure Db). We have also analysed other epicardial markers. Similarly, we observed a trend towards increased *tcf21* expression in the mutant epicardium with no obvious changes in *aldh1a2* and *wt1b* (Figure Db).

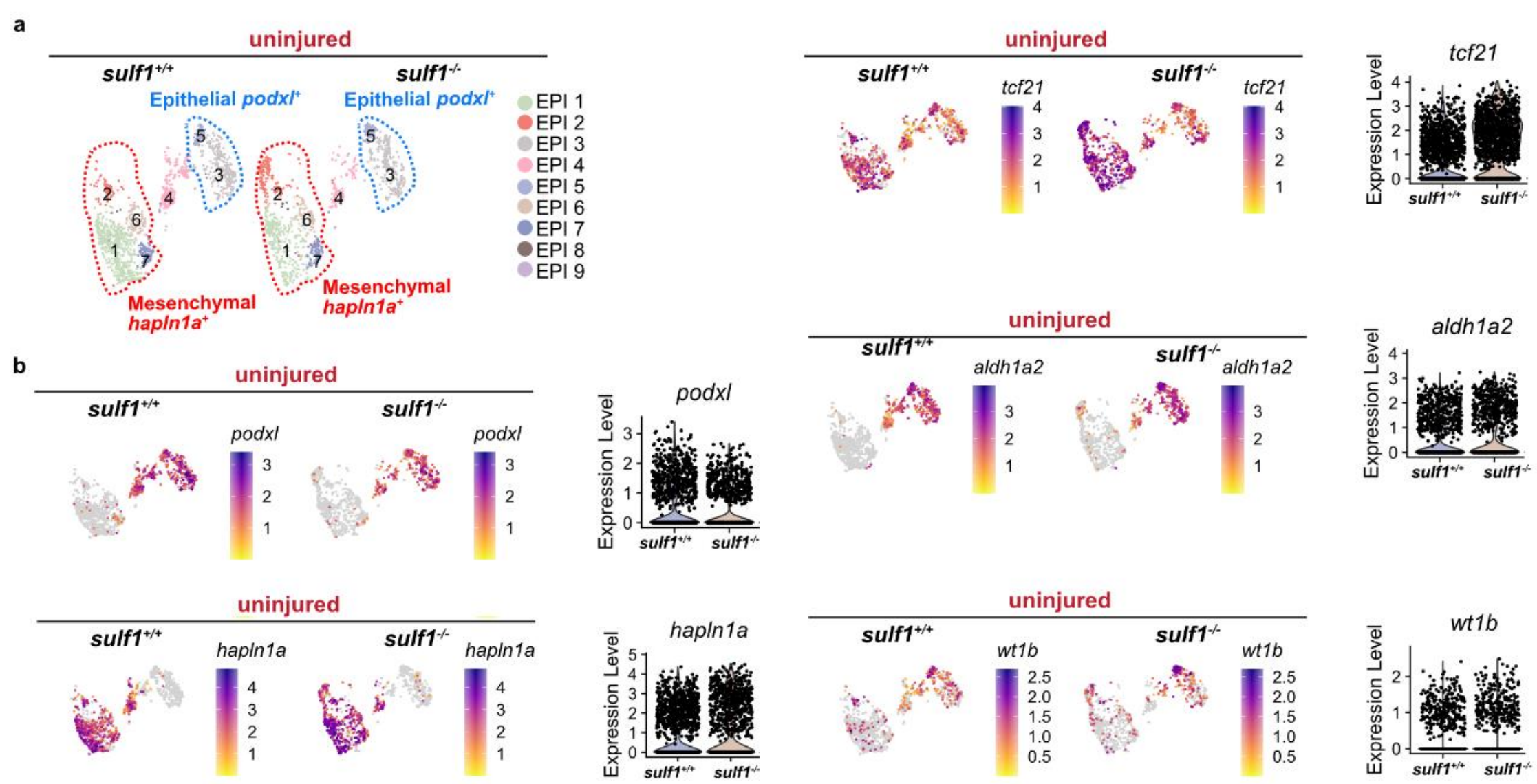

Figure D. a. UMAP representation of EPDC subclusters. b. UMAPs and violin plots showing expression of *podxl*, *hapln1a, aldh1a2, tcf21* and *wt1b* in wild-type and mutant EPDCs.

In their preprint, Redpath et al. report that while WT1 KOs were embryonically lethal, Sulf1 iKO phenotypes were transient and that the embryos were viable and showed similar composition of major cardiac cell types compared to controls. It is possible that similar phenotypes might be transiently present during epicardial development in *sulf1* mutant fish. Although the observed transcriptional changes appear subtle, we analysed adult ventricles and therefore cannot rule out this possibility. Future detailed developmental studies would be required to address this question.

2. Linked to the previous comment, the scRNAseq data in S3 shows a strong reduction in ECs in uninjured sulf1 mutant hearts. The authors do not elaborate on this as far as I can see. The authors should discuss the implementation of this reduction in ECs on regeneration outcome in sulf1 mutants.

We have now performed stainings on uninjured mutant hearts using the endothelial marker Fli1a (Appendix Figure S4A) and did not observe obvious differences in endothelial cell number or distribution compared to controls. Nevertheless, for this manuscript, we focused on the epicardial and immune phenotypes as these defects were the most prominent. The potential contribution of the cardiac endothelium to the

regenerative phenotypes will require further investigation. As requested, we have now discussed this point.

3. For their conclusions about changes in gene expression in sulf1 mutants the authors rely on the scRNAseq data. Based on the M&M the scRNAseq experiment was performed once without an independent replicate experiment. Therefore the authors should replicate and confirm some of their major findings and conclusions about altered gene expression using another technique such as by qPCR or ISH.

Findings from our scRNA-seq data have been validated and/or supported throughout the manuscript with previous and new data:

<u>Injury-induced epicardial activation phenotype</u>
We have performed HCR staining for epicardial markers *aldh1a2* and *ptx3a.* We performed qPCR analysis to complement our data showing *ptx3a* alterations and increased the n=7 as a measure of reproducibility for *aldh1a2* phenotype. (Figure 2C-E). We have also quantified EPDC expansion by quantifying *tcf21*$^{+}$ cells in the injured area (Figure 2F,G). We have now added epicardial explant data for the mutants (Figure 2H,I).

<u>Altered epicardial TGF-β signaling</u>
We have quantified pSmad3^{+} Cav1^{+} cells in the mutants as a readout of epicardial TGF-β signaling (Figure 3A,B).

<u>Increased macrophage number and inflammation in the mutant</u>
We have now quantified the number of *mpeg1.1*:mCherryF^{+} macrophages and L-plastin+ cells in *sulf1* mutants and wild-type siblings after cardiac injury (Figure 5B,C and Figure EV3A,B). We also performed TGF-β inhibitor experiments and showed by qPCR recapitulation of the transcriptional changes observed in *sulf1* mutant ventricles by scRNAseq. These data further support the increased inflammation phenotype observed in mutants (Figure 6C,D).

4. Authors write in line 306: 'Collectively, these findings indicate that Sulf1 regulates TGF-β signaling-mediated epicardial activation and EPDC regeneration which in turn is required for efficient fibrosis resolution.'. The conclusion that TGF-beta signaling is responsible for the pertained scar is a stretch as many studies have shown that reducing cardiomyocyte proliferation also affects the resolution of the temporal fibrosis. Therefore the observed effects on fibrosis could well be very indirect. The authors should be careful when drawing conclusions about fibrosis without further investigating the underlying mechanisms.

Thanks for raising this point. We have now added data showing increased number of aSMA^{+} myofibroblasts in *sulf1* mutant ventricles at 4 dpci (Figure 3G,H). We have also added HCR staining and qPCR analysis for *postnb*, a marker for activated fibroblasts (Figure 3I,J). These new data support that altered TGF-β signalling in *sulf1* mutants leads

to increased myofibroblast numbers which ultimately might contribute to the augmented fibrosis observed in mutants. Still, we agree with the reviewer that we should remain cautious when drawing conclusions of this type and have carefully revised the manuscript to avoid overstatements. These new data have also now been included in the revised manuscript.

Minor issues

5. Authors should also cite original research papers when referring to published work rather than only citing literature reviews. For example in line 290: 'After cardiac injury, CMs dedifferentiate and re-enter the cell cycle to rebuild the lost myocardium64,65.' Authors should include pmid 20336144 and 20336145. There are more examples of this throughout the manuscript.

We thank the reviewer for pointing this out. We have now carefully reassessed the citations and made necessary changes in the revised manuscript.

1st Revision - Editorial Decision 6th Jul 2026

Dear Dr. Marín-Juez

Thank you for the submission of your revised manuscript to EMBO reports. I apologize for the delay in handling your manuscript due to the large volume of recent submissions. We have now received the full set of referee reports that is copied below.

As you will see, all referees are very positive about the study and request only minor changes to clarify text and figures.

From the editorial side, there are also a few things that we need before we can proceed with the official acceptance of your study:

- Please put the manuscript sections in the following order: Title page - Abstract - Introduction - Results - Discussion - Methods - Data Availability - Acknowledgments - Disclosure Statement & Competing Interests - References - Figure Legends - (Main Tables with legends if applicable) - Expanded View Figure Legends.

- Please remove the following sections from p22-23: AUTHOR INFORMATION, ETHICS DECLARATION, APPENDIX

- Please rename the ETHICS DECLARATION/Disclosures statement to Disclosure and Competing Interests Statement.

- CRediT has replaced the traditional author contributions section because it offers a systematic machine readable author contributions format that allows for more effective research assessment. Please remove the Author Contributions section from the manuscript and use the free text boxes beneath each contributing author's name in our online systems to add specific details on the author's contribution. More information is available in our guide to authors.

- Please adjust the format of the reference list and of the in-text citations according to EMBO Press format. For instance, up to 10 author names in the reference list before et al and DOIs should only be used for preprints and datasets that have not been published yet. Please refer to our Guide to Authors for additional information on EMBO Press reference format.

- Please remove the separate Funding title and incorporate the information into the Acknowledgements section.

- Please update callouts for Table S1 to Appendix Table S1.

- Please remove the Reagents and Tools Table from the manuscript file.

- Please include species name for Experimental Models in the Reagents and Tools Table.

- Please remove the separate Appendix Figure files from the submission.

- Please upload the Appendix file in a clean PDF (without highlighting, track changes, etc.)

- Please note that EMBO press papers are accompanied online by:
A) a short (2 sentences) summary of the findings and their significance,
B) 2-5 short bullet points highlighting the key results, and
C) a synopsis image in .jpg or .png format that is exactly 550 pixels wide and 300 pixels high. Please note that the text needs to be legible at the final size. Please upload this information along with your revised manuscript (the text for A and B should be provided in one separate Word file uploaded as Cover Art/Synopsis Image).

- The Methods section "Single cell dissociation" bears high similarity to a prior publication. Please incorporate a citation for this section of the Methods.

- Our production/data editors have asked you to clarify several points in the figure legends (see below). Please incorporate these changes in the manuscript and return the revised file with tracked changes with your final manuscript submission.
- Please note that the exact p values are not provided in the legends of 5C; 6B.
- Please indicate the statistical test used for data analysis in the legends of figure 4H.
- Please note that the box plots need to be defined in terms of minima, maxima, centre, bounds of box and whiskers, and percentile in the legends of figures 2B,E,G,I; 3B,D,F,H,J,L,O; 5C; 6B,C,D,F,I; 7C,E,G,I,L; 8C,E,H; EV-1E; Appendix Figure S2-B,C; Appendix Figure S4-C.
- Please note that information related to n is missing in the legends 4G; EV-2F.
- Please note that the scale bar needs to be defined for figures EV-1D; EV-3A; Appendix Figure S1-A,C; Appendix Figure S4-B.
- Please note that the white dotted line is not defined in the legend of Appendix Figure S1-A. This needs to be rectified.
- Please note that the white arrowheads are not defined in the legend of Appendix Figure S1-A This needs to be rectified.

We look forward to seeing a final version of your manuscript as soon as possible.

With kind regards,

Kurt Weir
Editor
EMBO Reports

Referee #1:

The authors made a substantial effort to improve the manuscript, and nearly all of my comments have been addressed clearly, except for an issue concerning sulf1 expression in Appendix Figure S3D. In the provided images, the sulf1 HCR signals (magenta dots) appear to be more abundant in the sulf1 +/+ section than in the OE section. This trend appears to be the opposite of what would be expected and seems inconsistent with the corresponding data presented in Appendix Figure S3C. Clarification on this point would be appreciated.

Referee #2:

The authors have in a satisfactory manner addressed the issues I had raised (with the minor exceptions below), and significantly strengthened their conclusions with more convincing data. I can now recommend publication, if the authors can clarify these two remaining points:

6) Appendix Figure S4C: what is the "ventral" ventricular surface area? Since zebrafish have only one ventricle, this should be clarified.

7) fine, but where is this information? I couldn't find it. Please provide the altered text in the response to the reviewer.

Referee #3:

The authors have adequately addressed my previous concerns.

All editorial and formatting issues were resolved by the authors.

Dr. Rubén Marín-Juez
Centre Hospitalier Universitaire Sainte-Justine Research Centre
Montreal, Quebec H3T 1C5
Canada

Dear Dr. Marín-Juez,

I am pleased to inform you that your manuscript has been accepted for publication in EMBO reports. Your manuscript will be processed for publication by EMBO Press. It will be copy edited and you will receive page proofs prior to publication. Please note that you will be contacted by Springer Nature Author Services to complete licensing and payment information.

You may qualify for financial assistance for your publication charges - either via a Springer Nature fully open access agreement or an EMBO initiative. Check your eligibility: https://link.springer.com/journal/44319/how-to-publish-with-us

Should you be planning a Press Release on your article, please notify the production team via this form in order to coordinate publication and release dates: https://forms.cloud.microsoft/e/eH4A8JVP6g

If you have any questions, please do not hesitate to contact the Editorial Office. Thank you for your contribution to EMBO Reports.

Yours sincerely,

Kurt Weir
Editor
EMBO Reports

>>> Please note that it is EMBO Reports policy for the transcript of the editorial process (containing referee reports and your response letter) to be published as an online supplement to each paper. If you do NOT want this, you will need to inform the Editorial Office via email immediately. More information is available here: https://link.springer.com/partners/embo-press/editorial-policies#Peer%20review

EMBO Press Author Checklist

Corresponding Author Name: Rubén Marín-Juez
Journal Submitted to: EMBO reports
Manuscript Number: EMBOR-2025-63009V2

USEFUL LINKS FOR COMPLETING THIS FORM
The EMBO Journal - Author Guidelines
EMBO Reports - Author Guidelines
Molecular Systems Biology - Author Guidelines
EMBO Molecular Medicine - Author Guidelines

Reporting Checklist for Life Science Articles (updated January
This checklist is adapted from Materials Design Analysis Reporting (MDAR) Checklist for Authors. MDAR establishes a minimum set of requirements in transparent reporting in the life sciences (see Statement of Task: 10.31222/osf.io/9sm4x). Please follow the journal's guidelines in preparing your
Please note that a copy of this checklist will be published alongside your article.

Abridged guidelines for figures
1. Data
The data shown in figures should satisfy the following conditions:
- ➞ the data were obtained and processed according to the field's best practice and are presented to reflect the results of the experiments in an accurate and unbiased manner.
- ➞ ideally, figure panels should include only measurements that are directly comparable to each other and obtained with the same assay.
- ➞ plots include clearly labeled error bars for independent experiments and sample sizes. Unless justified, error bars should not be shown for technical
- ➞ if n<5, the individual data points from each experiment should be plotted. Any statistical test employed should be justified.
- ➞ Source Data should be included to report the data underlying figures according to the guidelines set out in the authorship guidelines on Data

2. Captions
Each figure caption should contain the following information, for each panel where they are relevant:
- ➞ a specification of the experimental system investigated (eg cell line, species name).
- ➞ the assay(s) and method(s) used to carry out the reported observations and measurements.
- ➞ an explicit mention of the biological and chemical entity(ies) that are being measured.
- ➞ an explicit mention of the biological and chemical entity(ies) that are altered/varied/perturbed in a controlled manner.
- ➞ the exact sample size (n) for each experimental group/condition, given as a number, not a range;
- ➞ a description of the sample collection allowing the reader to understand whether the samples represent technical or biological replicates (including how many animals, litters, cultures, etc.).
- ➞ a statement of how many times the experiment shown was independently replicated in the laboratory.
- ➞ definitions of statistical methods and measures:
  - common tests, such as t-test (please specify whether paired vs. unpaired), simple $\chi 2$ tests, Wilcoxon and Mann-Whitney tests, can be unambiguously identified by name only, but more complex techniques should be described in the methods section;
  - are tests one-sided or two-sided?
  - are there adjustments for multiple comparisons?
  - exact statistical test results, e.g., P values = x but not P values < x;
  - definition of 'center values' as median or average;
  - definition of error bars as s.d. or s.e.m.

Please complete ALL of the questions below.
Select "Not Applicable" only when the requested information is not relevant for your study.

Materials

Newly Created Materials	Information included in the manuscript?	In which section is the information available? (Reagents and Tools Table, Materials and Methods, Figures, Data Availability Section)
New materials and reagents need to be available; do any restrictions apply?	Not Applicable	Methods

Antibodies	Information included in the manuscript?	In which section is the information available? (Reagents and Tools Table, Materials and Methods, Figures, Data Availability Section)
For **antibodies** provide the following information:<br>- Commercial antibodies: RRID (if possible) or supplier name, catalogue number and or/clone number<br>- Non-commercial: RRID or citation	Yes	Reagents and Tools Table, Methods

DNA and RNA sequences	Information included in the manuscript?	In which section is the information available? (Reagents and Tools Table, Materials and Methods, Figures, Data Availability Section)
**Short novel DNA or RNA including primers, probes**: provide the sequences.	Yes	Reagents and Tools Table, Methods

Cell materials	Information included in the manuscript?	In which section is the information available? (Reagents and Tools Table, Materials and Methods, Figures, Data Availability Section)
**Cell lines:** Provide species information, strain. Provide accession number in repository **OR** supplier name, catalog number, clone number, and/**OR** RRID.	Not Applicable	
**Primary cultures:** Provide species, strain, sex of origin, genetic modification status.	Not Applicable	
Report if the cell lines were recently **authenticated** (e.g., by STR profiling) and tested for mycoplasma contamination.	Not Applicable	

Experimental animals	Information included in the manuscript?	In which section is the information available? (Reagents and Tools Table, Materials and Methods, Figures, Data Availability Section)
**Laboratory animals or Model organisms:** Provide species, strain, sex, age, genetic modification status. Provide accession number in repository **OR** supplier name, catalog number, clone number, **OR** RRID.	Yes	Reagents and Tools Table, Methods
**Animal observed in or captured from the field:** Provide species, sex, and age where possible.	Not Applicable	
Please detail **housing and husbandry conditions**.	Yes	Methods

Plants and microbes	Information included in the manuscript?	In which section is the information available? (Reagents and Tools Table, Materials and Methods, Figures, Data Availability Section)
**Plants:** provide species and strain, ecotype and cultivar where relevant, unique accession number if available, and source (including location for collected wild specimens).	Not Applicable	
**Microbes:** provide species and strain, unique accession number if available, and source.	Not Applicable	

Human research participants	Information included in the manuscript?	In which section is the information available? (Reagents and Tools Table, Materials and Methods, Figures, Data Availability Section)
If collected and within the bounds of privacy constraints report on age, sex and gender or ethnicity for all study participants.	Not Applicable	

Core facilities	Information included in the manuscript?	In which section is the information available? (Reagents and Tools Table, Materials and Methods, Figures, Data Availability Section)
If your work benefited from core facilities, was their service mentioned in the acknowledgments section?	Yes	Acknowledgements

Design

Study protocol	Information included in the manuscript?	In which section is the information available? (Reagents and Tools Table, Materials and Methods, Figures, Data Availability Section)
If study protocol has been **pre-registered, provide DOI in the manuscript**. For clinical trials, provide the trial registration number **OR** cite DOI.	Not Applicable	
Report the **clinical trial registration number** (at ClinicalTrials.gov or equivalent), where applicable.	Not Applicable	

Laboratory protocol	Information included in the manuscript?	In which section is the information available? (Reagents and Tools Table, Materials and Methods, Figures, Data Availability Section)
Provide DOI OR other citation details if **external detailed step-by-step protocols** are available.	Yes	Methods

Experimental study design and statistics	Information included in the manuscript?	In which section is the information available? (Reagents and Tools Table, Materials and Methods, Figures, Data Availability Section)
Include a statement about **sample size** estimate even if no statistical methods were used.	Yes	Figure legends
Were any steps taken to minimize the effects of subjective bias when allocating animals/samples to treatment (e.g. **randomization procedure**)? If yes, have they been described?	Yes	Methods
Include a statement about **blinding** even if no blinding was done.	Yes	Methods
Describe **inclusion/exclusion criteria** if samples or animals were excluded from the analysis. Were the criteria pre-established?<br><br>If sample or data points were omitted from analysis, report if this was due to attrition or intentional exclusion and provide justification.	Not Applicable	
For every figure, are **statistical tests** justified as appropriate? Do the data meet the assumptions of the tests (e.g., normal distribution)? Describe any methods used to assess it. Is there an estimate of variation within each group of data? Is the variance similar between the groups that are being statistically compared?	Yes	Figure legends and Methods

Sample definition and in-laboratory replication	Information included in the manuscript?	In which section is the information available? (Reagents and Tools Table, Materials and Methods, Figures, Data Availability Section)
In the figure legends: state number of times the experiment was **replicated** in laboratory.	Yes	Figure legends
In the figure legends: define whether data describe **technical or biological replicates**.	Yes	Figure legends

Ethics

Ethics	Information included in the manuscript?	In which section is the information available? (Reagents and Tools Table, Materials and Methods, Figures, Data Availability Section)
Studies involving **human participants**: State details of **authority granting ethics approval** (IRB or equivalent committee(s), provide reference number for approval.	Not Applicable	
Studies involving **human participants**: Include a statement confirming that **informed consent** was obtained from all subjects and that the experiments conformed to the principles set out in the WMA Declaration of Helsinki and the Department of Health and Human Services Belmont Report.	Not Applicable	
Studies involving **human participants:** For publication of **patient photos**, include a statement confirming that consent to publish was obtained.	Not Applicable	
Studies involving experimental **animals**: State details of **authority granting ethics approval** (IRB or equivalent committee(s), provide reference number for approval. Include a statement of compliance with ethical regulations.	Yes	Methods
Studies involving **specimen and field samples:** State if relevant **permits** obtained, provide details of authority approving study; if none were required, explain why.	Not Applicable	

Dual Use Research of Concern (DURC)	Information included in the manuscript?	In which section is the information available? (Reagents and Tools Table, Materials and Methods, Figures, Data Availability Section)
Could your study fall under dual use research restrictions? Please check biosecurity documents and list of **select agents and toxins** (CDC): https://www.selectagents.gov/sat/list.htm	Not Applicable	
If you used a select agent, is the security level of the lab appropriate and reported in the manuscript?	Not Applicable	
If a study is subject to dual use research of concern regulations, is the name of the **authority granting approval and reference number** for the regulatory approval provided in the manuscript?	Not Applicable	

Reporting

The MDAR framework recommends adoption of discipline-specific guidelines, established and endorsed through community initiatives. Journals have their own policy about requiring specific guidelines and recommendations to complement MDAR.

Adherence to community standards	Information included in the manuscript?	In which section is the information available? (Reagents and Tools Table, Materials and Methods, Figures, Data Availability Section)
State if relevant guidelines or checklists (e.g., **ICMJE, MIBBI, ARRIVE, PRISMA**) have been followed or provided.	Not Applicable	
For **tumor marker prognostic studies,** we recommend that you follow the **REMARK** reporting guidelines (see link list at top right). See author guidelines, under 'Reporting Guidelines'. Please confirm you have followed these guidelines.	Not Applicable	
For **phase II and III randomized controlled trials**, please refer to the **CONSORT** flow diagram (see link list at top right) and submit the CONSORT checklist (see link list at top right) with your submission. See author guidelines, under 'Reporting Guidelines'. Please confirm you have submitted this list.	Not Applicable	

Data Availability

Data availability	Information included in the manuscript?	In which section is the information available? (Reagents and Tools Table, Materials and Methods, Figures, Data Availability Section)
Have **primary datasets** been deposited according to the journal's guidelines (see 'Data Deposition' section) and the respective accession numbers provided in the Data Availability Section?	Yes	Data Availability
Were **human clinical and genomic datasets** deposited in a public access-controlled repository in accordance to ethical obligations to the patients and to the applicable consent agreement?	Not Applicable	
Are **computational models** that are central and integral to a study available without restrictions in a machine-readable form? Were the relevant accession numbers or links provided?	Not Applicable	
If publicly available data were reused, provide the respective **data citations in the reference list**.	Not Applicable	
